# A humanized mouse that mounts mature class-switched, hypermutated and neutralizing antibody responses

**DOI:** 10.1038/s41590-024-01880-3

**Published:** 2024-06-25

**Authors:** Daniel P. Chupp, Carlos E. Rivera, Yulai Zhou, Yijiang Xu, Patrick S. Ramsey, Zhenming Xu, Hong Zan, Paolo Casali

**Affiliations:** 1 The Antibody Laboratory, Department of Microbiology, Immunology & Molecular Genetics, The University of Texas Long School of Medicine, San Antonio, TX USA; 2 Department of Obstetrics & Gynecology, The University of Texas Long School of Medicine, San Antonio, TX USA; 3 Department of Medicine, The University of Texas Long School of Medicine, San Antonio, TX USA; 4 Present Address: Invivyd, Waltham, MA USA; 5 Present Address: Prellis Biologics, Berkeley, CA USA

**Keywords:** Antibodies, Class switch recombination, Somatic hypermutation, Vaccines, Autoimmunity

## Abstract

Humanized mice are limited in terms of modeling human immunity, particularly with regards to antibody responses. Here we constructed a humanized (THX) mouse by grafting non-γ-irradiated, genetically myeloablated *Kit*^W-41J^ mutant immunodeficient pups with human cord blood CD34^+^ cells, followed by 17β-estradiol conditioning to promote immune cell differentiation. THX mice reconstitute a human lymphoid and myeloid immune system, including marginal zone B cells, germinal center B cells, follicular helper T cells and neutrophils, and develop well-formed lymph nodes and intestinal lymphoid tissue, including Peyer’s patches, and human thymic epithelial cells. These mice have diverse human B cell and T cell antigen receptor repertoires and can mount mature T cell-dependent and T cell-independent antibody responses, entailing somatic hypermutation, class-switch recombination, and plasma cell and memory B cell differentiation. Upon flagellin or a Pfizer-BioNTech coronavirus disease 2019 (COVID-19) mRNA vaccination, THX mice mount neutralizing antibody responses to *Salmonella* or severe acute respiratory syndrome coronavirus 2 Spike S1 receptor-binding domain, with blood incretion of human cytokines, including APRIL, BAFF, TGF-β, IL-4 and IFN-γ, all at physiological levels. These mice can also develop lupus autoimmunity after pristane injection. By leveraging estrogen activity to support human immune cell differentiation and maturation of antibody responses, THX mice provide a platform to study the human immune system and to develop human vaccines and therapeutics.

## Abstract (web summary)

Humanized mice have been a valuable tool for modeling human immunology but are limited in their ability to model human antibody responses. Here the authors present their THX humanized mouse that does model human antibody responses and test its suitability for vaccination and autoimmunity studies.

## Main

Many of the more than the 1,600 immune response mouse genes are incongruent with their human equivalents, resulting in divergencies or deficiencies of mice as predictors of human immune responses^1^, making availability of a ‘humanized’ mouse model that faithfully reproduces human immune responses a high priority. The first humanized immune system mice were constructed by injecting human peripheral blood lymphocytes or (CD34^+^) human hematopoietic stem cells (huHSCs; hu prefix for human or humanized is used throughout) into severe combined immunodeficiency *Prkdc*^scid^ (SCID) mice or *Rag1/Rag2* knockout (KO) mice^2–6^. Subsequently, huHSC grafting of immunodeficient nonobese diabetic NOD.Cg-*Prkdc*^scid^ *Il2rg*^tm1Wjl^*/Sz* or NOD.Cg-*Prkdc*^scid^ *Il2rg*^null^ (NSG) mice^7,8^, in which *Il2rg* deletion results in defective cytokine signaling in multiple immune cell receptors, furthered the scope of humanized mice^8^. In huNSG mice, the NOD phagocytic cell SIRPα receptor variant cross-reacts with human CD47 to induce a ‘don’t eat me’ signal, thereby limiting human cell phagocytosis^5,9^. NSG mice, however, allow for poor huHSC accessibility to the bone marrow (BM) hematopoietic niche^5–8^, a limitation only partially obviated by mouse myeloablation through γ-radiation, which, however, increases risk of wasting, infection and mortality^5^. In addition, huNSG mice remain poor immune responders. Attempts to make them better responders have included knock-in or transgenic insertion of cytokine genes, generally resulting, however, in abnormal supraphysiological cytokine expression^2–6^.

Although mutated IgG to ovalbumin have been detected in γ-irradiated knock-in hu*IL6 Rag2*^−/−^*Il2rg*^−/−^*SIRP*α^h/m^ mice (RG SKI interleukin (IL)-6)^10^, a humanized mouse capable of mounting fully mature antibody responses has yet to be established. Maturation of the antibody response entails B cell somatic hypermutation (SHM), class-switch DNA recombination (CSR), differentiation of plasma cells (PCs) making high-affinity antibodies and generation of specific memory B cells (MBCs). The National Institute of Allergy and Infectious Diseases has emphasized the need for a novel and more advanced human immune system mouse model^2^, a recommendation that has gone essentially unheeded. Generation of homozygous *Kit*^W-41J^ mutant NSG mice has yielded genetically myeloablated NSGW41 (NOD.Cg-*Kit*^W-41J^*Prkdc*^scid^*Il2rg*^tm1Wjl^/WaskJ) and NBSGW (NOD.Cg-*Kit*^W-41J^*Tyr*^+^*Prkdc*^scid^*Il2rg*^tm1Wjl^/ThomJ) mice, supporting huHSC engraftment without γ-radiation^11,12^. Mutated *Kit*^W-41J^ hampers mouse (mo)HSCs docking onto BM stromal cells and opens up an ample niche for huHSCs docking through binding of mouse stem cell factor^11,12^, which is engaged by huHSC c-Kit. Adult NBSGW and NSGW41 mice grafted intravenously with cord blood huCD34^+^ cells supported greater huCD45^+^ lymphoid and myeloid cell reconstitution than γ-irradiated NSG mice^5,11,12^. Despite their obvious potential, however, NBSGW and NSGW41 mice have not been leveraged to construct an advanced humanized mouse that faithfully replicates human immune responses^2–6^.

We created a humanized (THX) mouse by grafting NBSGW^12^ and NSGW41 (ref. ^11^) neonates with cord blood huCD34^+^ cells through intracardiac injection, followed by conditioning with 17β-estradiol (E2), the most potent and physiologically abundant estrogen. E2 supports differentiation of HSCs^13–15^, lymphoid and myeloid immune cells, including marginal zone (MZ) B cells, follicular helper T (T_FH_) cells, germinal center (GC) B cells, MBCs and granulocytes, all expressing estrogen receptors ERα and ERβ^13–26^. E2 also boosts B cell AID and BLIMP-1 expression, enabling SHM/CSR and PC differentiation^27–30^. THX mice reconstitute a human immune system, including peripheral lymph nodes (LNs), Peyer’s patches and human thymic epithelial cells (huTECs). They mount mature neutralizing antibody responses to *Salmonella* (*S*.) Typhimurium and severe acute respiratory syndrome coronavirus 2 (SARS-CoV-2) Spike S1 receptor-binding domain (RBD), together with B cell-related cytokines. Finally, THX mice are amenable to develop systemic lupus autoantibodies and immunopathology.

## Results

### THX mice support full and sustained development of human immune cells

**Fig. 1:**
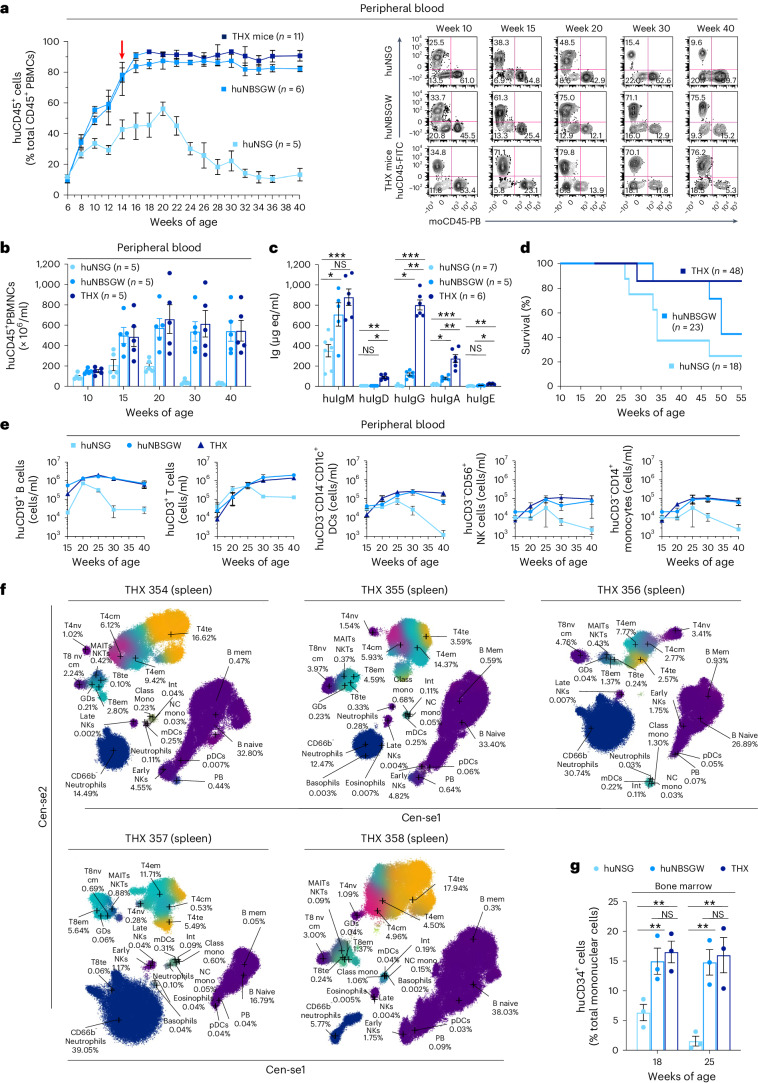
THX mice support full and sustained development of human immune cells. **a**, Left, huCD45^+^ PBMCs reconstitution at indicated time points in THX (*n* = 11), huNBSGW (*n* = 6) and huNSG (*n* = 5) mice grafted with cord blood huCD34^+^ cells. Engraftment levels depicted as percentage of total (human plus mouse) CD45^+^ PBMCs. Arrow denotes the beginning of E2 treatment in huNBSGW mice that would later become THX mice (dark navy line starting at 18 weeks of age) and continuing thereafter. Right, Human and mouse CD45^+^ PBMCs (% total PBMCs), as identified by flow cytometry. Fluorescence-activated cell sorting (FACS) plots are from one THX, one huNBSGW and one huNSG mouse, each representative of five mice. **b**, huCD45^+^ mononuclear cell counts in THX, huNBSGW and huNSG mice. **c**, Total serum huIgM, huIgD, huIgG, huIgA and huIgE (expressed as µg equivalents per ml, µg eq ml^−1^) in THX (*n* = 6), huNBSGW (*n* = 5) and huNSG (*n* = 7) mice—huIgD and huIgE were undetectable in huNBSGW and huNSG mice. **d**, Survival of THX (*n* = 48), huNBSGW (*n* = 23) and huNSG (*n* = 18) mice through 55 weeks after huCD34^+^ cell engraftment (Kaplan–Meier curves, THX versus huNSG mice, *P* = 0.0323; THX versus huNBSGW mice, *P* = 0.1809, log-rank Mantel–Cox test). **e**, Number of huB cells (huCD45^+^CD19^+^), huT cells (huCD45^+^CD3^+^), huDCs (huCD45^+^CD3^−^CD14^−^CD11c^+^), huNK cells (huCD45^+^CD3^−^CD56^+^) and human monocytes (huCD45^+^CD3^−^CD14^+^) per ml of peripheral blood in THX, huNBSGW and huNSG mice (same mice as in **b**). **f**, Human immune cell profiling of THX mouse (*n* = 5) spleen huCD45^+^ cells analyzed by high-parameter cytometry with time-of-flight (CyTOF) analysis of 30 human markers. THX mouse spleen huCD45^+^ lymphoid and myeloid cell proportions were similar to those in spleens of humans (*n* = 6) who died from accidental death (Supplementary Table 4g). **g**, BM huCD34^+^ cells in THX (*n* = 3), huNBSGW (*n* = 3) and huNSG mice (*n* = 3). In the histograms (**b**, **c** and **g**), each dot represents an individual mouse, and the bar depicts the mean with s.e.m. Statistical significance (**c** and **g**) was assessed using two-sided Student’s unpaired *t*-test (NS, not significant; *P < 0.05, **P < 0.01, ***P < 0.001). Source data

**Extended Data Fig. 1:**
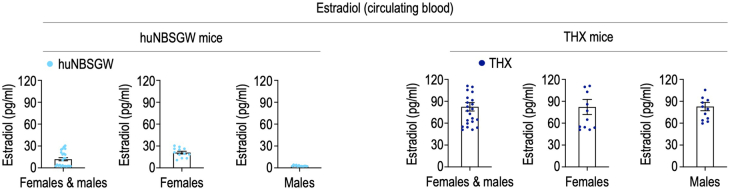
Serum 17β-estradiol concentrations in humanized mice. Serum estradiol concentrations (pg ml^−1^) in non-intentionally immunized female (*n* = 12) and male (*n* = 12) THX mice and non-intentionally immunized (non-E2-treated) female (*n* = 12) and male (*n* = 12) huNBSGW mice measured by specific ELISA (Cayman Chemical estradiol platform). Each dot in histograms depicts E2 concentration from an individual mouse and the bar depicts the mean with s.e.m. Estradiol concentrations in female and male THX mice were comparable and significantly greater than in huNBSGW mice (*P* < 0.0001, two-sided Student’s unpaired *t-*test). The normal blood estradiol concentration in mice can vary depending on factors such as age, sex and stage of the estrous cycle in females. In female C57BL/6 mice, blood estradiol concentration range is as follows: Proestrus (the stage just before estrus), 5–60 pg ml^−1^; Estrus (the stage when ovulation occurs), 15–200 pg ml^−1^; Metestrus (the stage just after estrus), 5–50 pg ml^−1^; Diestrus (the stage between metestrus and proestrus), 5–40 pg ml^−1^. In male mice, blood estradiol concentrations are lower ( < 5.0 pg ml^−1^). In women, blood estradiol concentration range is as follows: Follicular phase (days 1–14 of the menstrual cycle), 35–400 pg ml^−1^; Mid-cycle (around day 14 of the menstrual cycle), 100–500 pg ml^−1^; Luteal phase (days 14–28 of the menstrual cycle), 35–400 pg ml^−1^; Postmenopausal women, less than 10–30 pg ml^−1^. In pregnant women, blood estradiol concentration range is as follows: First trimester, 300–3,000 pg ml^−1^; Second trimester, 1,900–10,000 pg ml^−1^; Third trimester, 2,000–14,000 pg ml^−1^. In men, blood estradiol concentration range is 10–30 pg ml^−1^. It is important to note that estradiol concentration ranges may vary depending on the laboratory that performs the test and the assay used for measurement. Blood estradiol concentration ranges reported here were derived from multiple sources^80–86^. Source data

**Extended Data Fig. 2:**
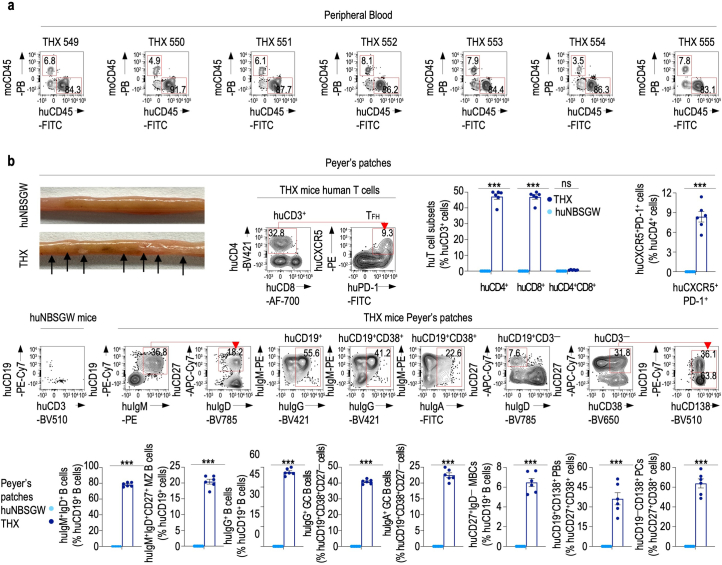
THX mice huCD45^+^ cell reconstitution and THX mice but not huNBSGW mice develop Peyer’s patches, containing huB cells, huMZ B cells, huGC B cells, huMBCs, huPBs/PCs and huT cells. **a**, Identification of circulating huCD45^+^ PBMCs in non-intentionally immunized THX mice (*n* = 7) by flow cytometry. huCD45^+^ cells account for 92−96% of total (human plus mouse) CD45^+^ cells in blood of THX mice. **b**, THX (*n* = 6 of the 7 as in Fig. 4g) and huNBSGW (*n* = 6 of the 7 as in Fig. 4g) mice were injected i.p. with DNP-CpG (50 μg in 100 µl PBS) at day 0, boosted on day 14 and euthanized on day 28. (Top left row) Peyer’s patches in THX mice and lack thereof in huNBSGW mice. (Top right row) huCD3^+^CD4^—^CD8^—^, huCD3^+^CD4^+^, huCD3^+^CD8^+^, huCD3^+^CD4^+^CD8^+^ T cells and huCD3^+^CD4^+^CXCR5^+^PD-1^+^ T_FH_ cells. (Middle row) MZ huCD19^+^IgM^+^IgD^+^CD27^+^ B cells (20.4% ± 0.7% huB cells), huIgM^+^ and huIgG^+^ B cells, GC huCD19^+^CD38^+^CD27^—^IgG^+^ and GC huCD19^+^CD38^+^CD27^—^IgA^+^ B cells, class-switched memory huCD27^+^huIgD^—^ B cells, huCD19^+^CD27^+^CD38^+^CD138^+^ PBs and huCD19^—^CD27^+^CD38^+^CD138^+^ PCs in Peyer’s patches of DNP-CpG-immunized THX and huNBSGW mice. Due to the extreme paucity of cells in barely detectable Peyer’s patches of huNBSGW mice, not enough events could be collected for a meaningful analysis. Flow cytometry plots are from one THX and one huNBSGW mouse, each representative of 6 mice. huCD45^+^ cells were pre-gated in all FACS analyses. Captions on top of FACS plots indicate pre-gating markers. (Bottom row) Quantification of huMZ B cells, class-switched huB cells, huGC B cells, huMBCs and huPBs/PCs in Peyer’s patches of THX and huNBSGW mice. Each dot represents an individual mouse, the bar depicts the mean with s.e.m. Statistical significance was assessed by two-sided Student’s unpaired *t*-test (NS, not significant; ***P < 0.001). Source data

To make huNBSGW and huNSG mice, we injected intracardially (left ventricle) NBSGW and γ-irradiated NSG neonates with cord blood huCD34^+^ cells. To make THX mice, we fed huNBSGW mice E2 ad libitum in drinking water starting at 14 to 18 weeks of age. After 4 weeks, THX mice were ready for experimental use or continued on E2 for use at a later time. Female and male THX mice showed comparable blood E2 levels (82.17 ± 10.36 pg ml^−1^ and 82.75 ± 5.72 pg ml^−1^, respectively, mean ± s.e.m.), higher than those in female and male huNBSGW mice (20.94 ± 1.88 and <5 pg ml^−1^) and within women’s physiological E2 level (35–500 pg ml^−1^; Extended Data Fig. 1 and Supplementary Table 1). THX and huNBSGW mice sustained human peripheral blood mononuclear cells (huPBMCs) at higher levels (up to 96.1% and 89.3% huCD45^+^ cells, respectively) than huNSG mice (Fig. 1a,b and Extended Data Fig. 2a). They showed more blood huB cells, huT cells, human dendritic cells (huDCs), human natural killer (huNK) cells and human monocytes, and more huB cells in spleen and LNs than huNSG mice (Fig. 1e and Supplementary Figs. 1 and 2). THX mice displayed higher levels of circulating huIgM, huIgD, huIgG, huIgA and huIgE, and had a longer lifespan than huNBSGW and huNSG mice (Fig. 1c,d). Their spleens contained a spectrum of huCD45^+^ lymphoid and myeloid cells, like spleens of humans who died from accidental death^31^ (Fig. 1f and Supplementary Tables 2–4a–g). THX mice showed blood huCD45^−^CD235a^−^CD61^+^ platelets and, as in other humanized mice, few huCD235a^+^ red blood cells^5^ (Supplementary Table 5a,b). THX and huNBSGW mice harbored more BM huCD34^+^ cells than huNSG mice (Fig. 1g). Thus, female and male THX mice reconstitute human lymphoid and myeloid cells, showed higher levels of huIgM, huIgD, huIgG, huIgA and huIgE than huNBSGW and huNSG mice, and extended survival.

### THX mice BCR huV(D)J gene repertoire reflects that of humans

**Fig. 2:**
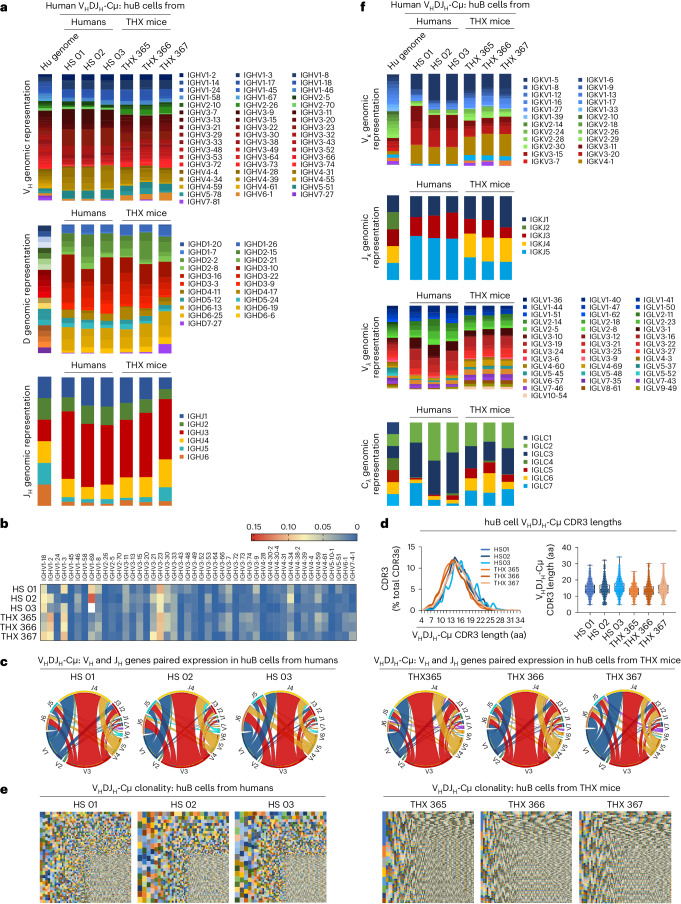
THX mice huBCR repertoire and clonality are similar to those in humans. **a**, huIgH V_H_, D and J_H_ gene genomic representation and expression in blood and spleen huIgM^+^ B cells of healthy humans (*n* = 3, HS 01, 02, 03) and non-intentionally immunized THX mice (*n* = 3, THX 365, 366, 367), depicted as stacked columns. In these, different colors denote different huV_H_, huD or huJ_H_ gene families; color gradients denote individual family members—the huIgH locus haploid complement consists of 36–49 functional huV_H_ genes segregated into 7 families^32^. **b**, Heat map of individual huV_H_ family members in huIgM^+^ B cells of HS and THX mice as in **a**. **c**, Associated expression of huV_H_ and huJ_H_ genes in huIgM^+^ B cell repertoire of HS and THX mice as in **a**, depicted by Circos plots. Outermost Circos plot tracks mark the boundaries of each huV_H_ or huJ_H_ region subfamily. **d**, huIgH CDR3 (translated amino acid sequence) length distribution (left) and frequency (right) in huIgM^+^ B cell recombined huV_H_DJ_H_-Cμ transcripts of HS and THX mice as in **a**—the somatically generated IgH CDR3 is the most polymorphic BCR region and provides the main structural correlate for antigen binding. In the violin plots, the upper and lower edges of the box plot indicate the 75th and 25th percentiles, respectively, and the middle line indicates the median. Each dot depicts CDR3 length in an individual huB cell. **e**, huB cell clones in HS and THX mice as in **a**, as identified by unique huV_H_DJ_H_-Cμ (including CDR3 as translated amino acid sequence) transcripts and depicted by TreeMaps. Individual rectangle or square (unique color) area reflects huB cell clone size. In THX mice, huV_H_DJ_H_-Cμ transcripts identified 521,859, 23,052 and 20,045 discrete huB cell clones in the same order of magnitude as in HS huV_H_DJ_H_-Cμ transcripts, which identified 11,115, 9,016 and 11,570 huB cell clones. **f**, huIgK chain (Vκ and Jκ) and huIgL chain (Vλ and Jλ-Cλ) gene genomic representation and expression in huIgM^+^ B cells of HS and THX mice as in **a**, depicted as stacked columns. In these, different colors denote different huVκ, huJκ or huVλ, huCλ gene families; color gradients denote individual gene family members—the huIgκ locus comprises 39 functional huVκ genes and 5 huJκ genes, while the huIgλ locus comprises 30 functional huVλ genes segregated into 10 subgroups and 5 functional huJλ-Cλ clusters^32^.

The THX mouse huBCR repertoire mirrored that of humans. Indeed, THX mouse huCD19^+^IgM^+^ B cells expressed huV_H_DJ_H_-Cμ transcripts with probabilistic V_H_ gene usage, that is, reflecting the genomic representation of human V_H_ genes (huIgH locus haploid complement consists of 36–49 functional V_H_ genes segregated in seven families^32^), with V3 family genes, particularly V3–V30, as the most frequently utilized, followed by V1 and V4 (Fig. 2a,b). Like humans, THX mouse huIgM^+^ B cells showed preponderant human D3 and J_H_3 utilization and dominant V3 to J_H_4 combination (Fig. 2c). Their huV_H_DJ_H_-Cμ transcripts showed a pseudo-normal CDR3 length distribution, which peaked at 14 amino acids, mimicking huIgM^+^ B cells in humans (Fig. 2d). Discrete huIgM^+^ B cell clones identified by unique and identical huV_H_DJ_H_-Cμ transcripts showed even greater diversity than in humans (Fig. 2e). THX mouse huIgM^+^ B cells displayed a Vκ gene utilization similar to that of humans^32^, albeit biased to Vκ4, and a Jλ-Cλ3 utilization versus human huIgM^+^ B cells Jλ-Cλ2 and Jλ-Cλ3 (Fig. 2f). Thus, the THX mouse huIgM^+^ BCR repertoire mirrors that of humans, with minor differences in VκJκ and VλJλ gene expression.

### THX mice transition from a mouse to a human-like intestinal microbiome

**Extended Data Fig. 3:**
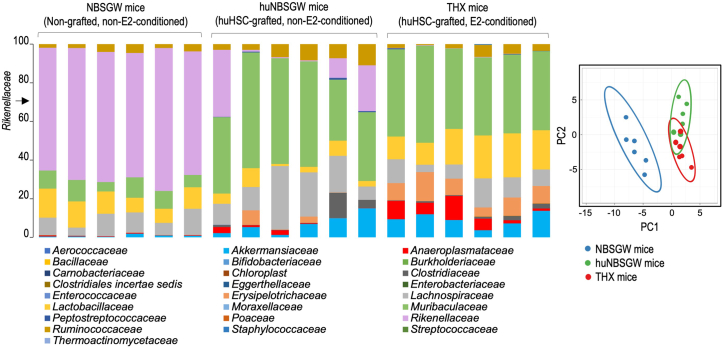
Gut microbiome composition in NBSGW, huNBSGW and THX mice. Left, bacterial families identified in gut microbiome of non-intentionally immunized (non-huHSC-grafted, non-E2-conditioned) NBSGW (*n* = 6), (huHSC-grafted, non-E2-conditioned) huNBSGW (*n* = 6) and (huHSC-grafted, E2-conditioned) THX mice (*n* = 6 including the 3 mice as in Fig. 2a–f) by high-throughput 16 s rRNA gene MiSeq amplicon sequencing. In histograms, different colors denote different bacterial families, depicted as stacked columns. Each column depicts microbiome composition in an individual mouse. THX, huNBSGW and NBSGW mice developed distinct gut bacterial microbiomes (THX, 8; huNBSGW, 7-8; NBSGW, 6 families). THX mice gut was colonized by *Lactobacillaceae*, *Lachnospiraceae, Erysipelotrichaceae* and *Clostridiaceae* bacterial families (phylum: Firmicutes), *Muribaculaceae* (Bacteroidetes), *Akkermansiaceae* (Verrucomicrobia) and *Enterobacteriaceae* (Pseudomonadota). NBSGW mice gut harbored predominately (up to virtually 80%) *Rikenellaceae* (Bacteroidetes), which are characteristic of mouse gut microbiome and were not found in THX mice. *Rikenellaceae* contributed moderately to gut microbiome of 3 out of 6 huNBSGW mice, suggesting a human pseudo-normalization of the mouse microbiome by human immune system elements’ development and differentiation. Disappearance of *Rikenellaceae* in THX mice suggested an important impact of E2 conditioning on further ‘humanization’ of these mice gut microbiome. Right, principal component analysis (PCA) of gut bacterial composition in the same non-intentionally immunized NBSGW (blue), huNBSGW (green) and THX (red) mice. Each dot depicts an individual mouse; colors denote NBSGW, huNBSGW and THX mice. THX and huNBSGW mice, both hosting bacterial families contributing to gut microbiota in healthy humans, fully segregate from NBSGW mice, which host predominately ‘murine’ *Rikenellaceae*. Source data

The BCR repertoire underpins antibody diversity, which, in turn, conditions gut microbiome composition^33^. Non-intentionally immunized THX, huNBSGW and NBSGW mice showed distinct and shared gut bacteria families (Extended Data Fig. 3). *Muribaculaceae* together with other families contributing to the human gut microbiome^34^ made up for the THX mouse microbiome. This shared most bacteria families with huNBSGW mice and substantially differed from that of NBSGW mice, which was dominated by the characteristically ‘murine’ *Rikenellaceae*, still found in the ‘transitional’ microbiome of huNBSGW mice, but absent in THX mice. Thus, reflecting the impact of human immune cells and E2, the THX gut microbiome consists of bacteria all found in human gut microbiome and shows little similarity to that of (non-grafted) NBSGW mice.

### THX mice huTCRa and huTCRb gene repertoires reflect those of humans

**Fig. 3:**
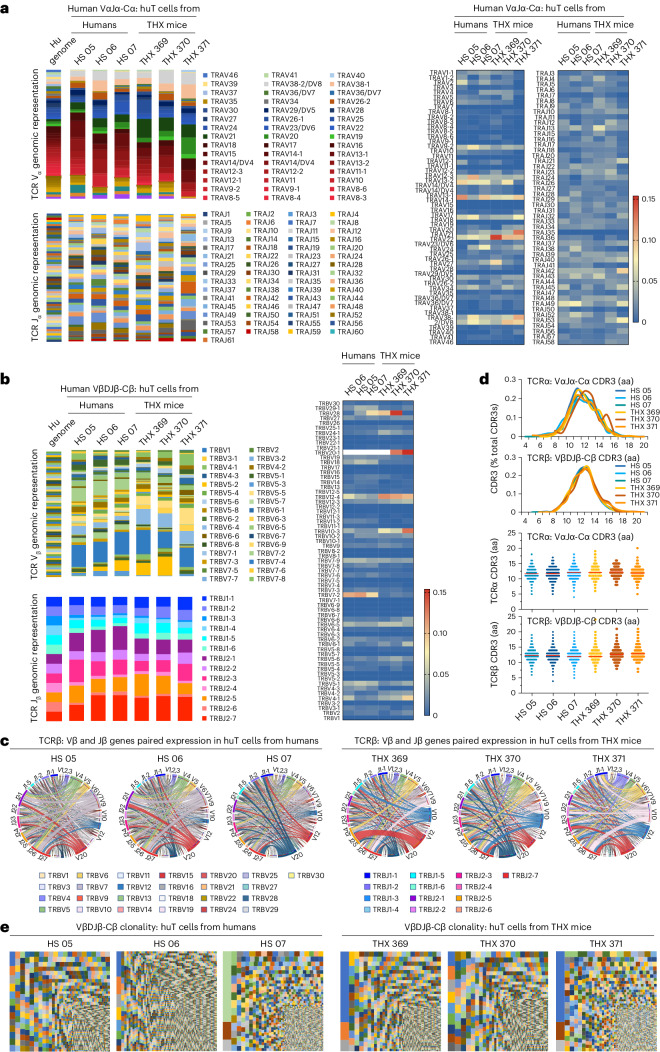
THX mice huTCR cell repertoire and clonality are similar to those in humans. **a**, huVα and huJα (huTCRa) genomic representation and gene expression in blood and spleen huT cells of healthy humans (*n* = 3, HS 05, 06, 07) and non-intentionally immunized THX mice (*n* = 3, THX mice 369, 370, 371) depicted as stacked columns (left). In these, different colors denote different huVα or huJα gene families; color gradients denote individual family members. Heat maps of expressed individual huVα and huJα genes (right). **b**, huVβ and huJβ (huTCRb) genomic representation and gene expression in huT cells of HS and THX mice as in **a**, depicted as stacked columns (left). In these, different colors denote different huVβ or huJβ gene families; color gradients denote individual family members. Heat maps of individual huVβ and huJβ genes (right). **c**, Associated expression of huVβ and huJβ genes in HS and THX mouse huT cell repertoires as in **a**, depicted by Circos plots. **d**, CDR3 length distribution (top) and frequency (bottom) in huT cell recombined huVαJα-Cα (huTCRa) and huVβDJβ-Cβ (huTCRb) transcripts of HS and THX mice as in **a**. Each dot depicts CDR3 length in an individual cell. **e**, huT cell clones in HS and THX mice as in **a**, as identified by unique huVβDJβ-Cβ (including CDR3 as translated amino acid sequence) transcripts and depicted by TreeMaps. Individual rectangle or square (unique color) area reflects huT cell clone size. In THX mice, huVβDJβ-Cβ transcripts identified 5,531, 3,981 and 8,142 discrete huT cell clones in the same order of magnitude as in HS huVβDJβ-Cβ transcripts, which identified 3,437, 11,305 and 4,266 discrete huT cell clones.

THX mouse spleen huTCRα and huTCRβ repertoire diversity largely reflected the human genomic representation of huVα, huJα, huVβ and huJβ genes^32^ (Fig. 3a), and broadly overlapped with huTCRα and huTCRβ gene expression in human blood, including huVβ and huJβ gene pair preferences (Fig. 3b,c). THX mice huVαJα−Cα and huVβDJβ−Cβ CDR3 lengths followed a pseudo-normal distribution, 4 to 21 amino acids, peaking at 11 and 13 amino acids, comparable to huT cells in humans (Fig. 3d). THX mouse huVβDJβ−Cβ transcripts identified discrete huT cell clones of a diversity comparable to humans (Fig. 3e). Thus, THX mouse huT cells express diverse huTCRα and huTCRβ repertoires, with huVα, huJα, huVβ and huJβ gene utilization reflecting huVα, huJα and huVβ, huJβ genomic representation and overlapping with that of huTCRα and huTCRβ in humans.

### THX mice mount a T cell-dependent-specific and mature antibody response

**Fig. 4:**
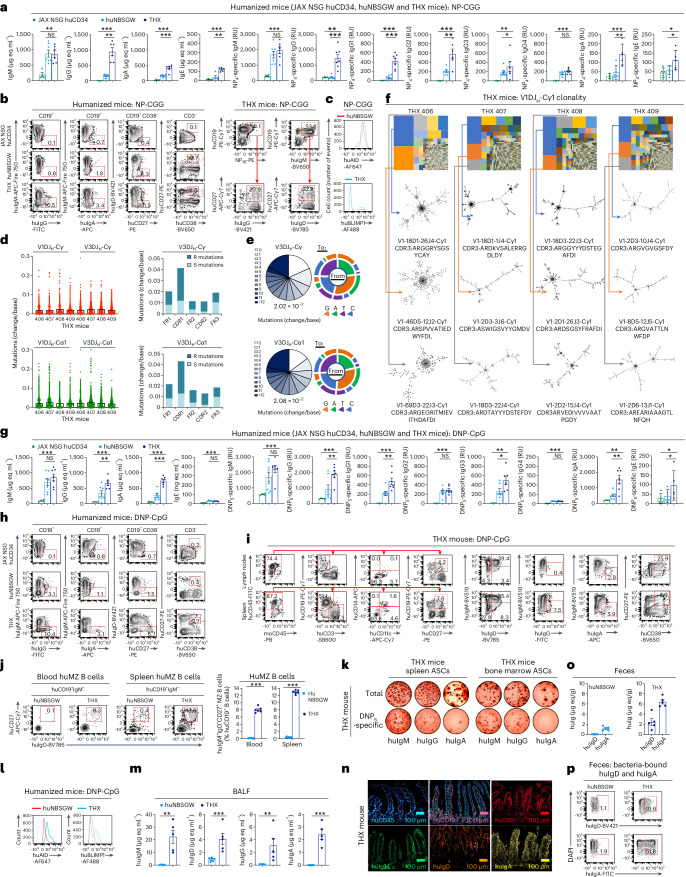
THX mice mount specific T cell-dependent and T cell-independent class-switched, hypermutated and clonal antibody responses. **a**–**f**, THX (*n* = 7), huNBSGW (*n* = 7) and JAX NSG huCD34 (*n* = 4) mice were injected i.p. with NP_16_-CGG (100 μg in 100 μl alum) on day 0, boosted (100 μg in 100 μl PBS) on day 14 and euthanized on day 28. **a**, Total serum human immunoglobulin and NP_4_-specific human antibodies measured by ELISAs. Total human immunoglobulin concentrations expressed as μg eq ml^−1^ and NP_4_-specific human antibodies expressed as relative units (RUs). Fewer than seven data points were derived for human immunoglobulins other than NP_4_-specific huIgM, huIgG and huIgG1. **b**, Left, spleen huIgM^+^, huIgG^+^ and huIgA^+^ B cells, class-switched memory huCD27^+^IgD^−^ B cells and huCD27^+^CD38^+^ PBs/PCs. Right, NP-specific huCD19^+^ B cells and memory huCD19^+^CD27^+^IgG^+^ B cells (identified by binding of PE-labeled NP_16_), and MZ huCD19^+^IgM^+^IgD^+^CD27^+^ B cells in THX mouse spleen. **c**, Spleen huB cell intracellular AID and BLIMP-1 expression in THX and huNBSGW mice. **d**, Left, point mutation frequencies (change/base) in spleen huB cell huV_H_DJ_H_-C_H_ transcripts of THX 406, 407, 408 and 409 mice depicted as scatterplots. Each dot represents a single sequence and the bar depicts the mean with s.e.m. Right, means of total, S and R huV3 mutation frequencies in FR1, CDR1, FR2, CDR2 and FR3 of huV_H_DJ_H_-C_H_ transcripts depicted as histograms. **e**, In the SHM pie charts, slices depict proportions of huV_H_DJ_H_-C_H_ transcripts carrying given numbers of point mutations; slice gray gradients depict increasing numbers of point mutations; the overall mutation frequency is listed below each pie chart. Spectrum of point mutations depicted as donut charts (same mice as in **d**). **f**, huV1DJ_H_-Cγ1 B cell clones and intraclonal diversification in THX mice (same mice as in **d**) depicted by TreeMaps and phylogenetic trees. Individual rectangle or square (unique color) area reflects huB cell clone size. In THX 406, 407, 408 and 409 mice, the three largest huV1DJ_H_-Cγ1 clones accounted for 42.9%, 26.6%, 32.0% and 23.4% of huV1DJ_H_-Cγ1 B cells. **g**–**p**, THX (*n* = 7), huNBSGW (*n* = 7) and JAX NSG huCD34 (*n* = 4) mice were injected i.p. with DNP-CpG (50 μg in 100 μl PBS) on day 0, boosted (50 μg in 100 μl PBS) on day 14 and euthanized on day 28. **g**, Total serum immunoglobulin concentration (μg eq ml^−1^) and DNP_5_-specific human antibodies (RUs) measured by specific ELISAs. Fewer than seven data points were derived for DNP-specific huIgE. **h**, Spleen huB cells, huMBCs and huPBs/PCs as in **b**. **i**, huCD45^+^ huCD19^+^ B cells, huCD3^+^ T cells, huCD11c^+^ DCs, huCD14^+^ monocytes, memory huCD19^+^CD27^+^ B cells, huCD27^+^CD38^+^ PBs/PCs as well as huIgM^+^, huIgD^+^, huIgG^+^ and huIgA^+^ B cells in THX mouse mesenteric LNs and spleen. **j**, Blood and spleen MZ huCD19^+^IgM^+^IgD^+^CD27^+^ B cells (8.1% ± 0.3% and 12.9% ± 0.2% huB cells, respectively) in THX (*n* = 6) and huNBSGW (*n* = 6) mice. FACS plots are from one THX and one huNBSGW mouse, each representative of six mice. **k**, Total and DNP_5_-specific huIgM, huIgG and huIgA ASCs in THX mouse spleen and BM, as analyzed by specific ELISPOTs. **l**, Spleen huB cell intracellular AID and BLIMP-1 expression in THX and huNBSGW mice. **m**, Total human immunoglobulin (μg eq ml^−1^) in the BALF of THX (*n* = 5) and huNBSGW (*n* = 5) mice. **n**, huCD45^+^ cells, huCD19^+^ B cells, huCD3^+^ T cells, and huIgM-, huIgD- and huIgA-producing cells in THX mouse lamina propria (immunofluorescence; scale bar, 100 μm). Different pseudocolors denote different cells. **o**,**p**, Free and bacteria-bound huIgD and huIgA in feces of THX (*n* = 5) and huNBSGW (*n* = 5) mice measured by specific ELISA (μg eq/g, THX versus huNBSGW mice: huIgD, *P* < 0.0001; huIgA, *P* = 0.007, two-sided Student’s unpaired *t*-test) and identified by flow cytometry (% total bacterial cells). Flow cytometry plots (**b**, **c**, **h**, **i** and **l**) are from one THX, one huNBSGW or one JAX NSG huCD34 mouse, each representative of three mice. huCD45^+^ cells were pre-gated in all FACS analyses. ELISPOT images (**k**) and micrographs (**n**) are from one THX mouse representative of five mice. In the histograms (**a**, **g**, **j**, **m** and **o**), each dot represents an individual mouse and the bar depicts the mean with s.e.m. Statistical significance (**a**, **g**, **j** and **m**) was assessed by two-sided Student’s unpaired *t*-test (NS, not significant; *P < 0.05, **P < 0.01, ***P < 0.001). Source data

**Extended Data Fig. 4:**
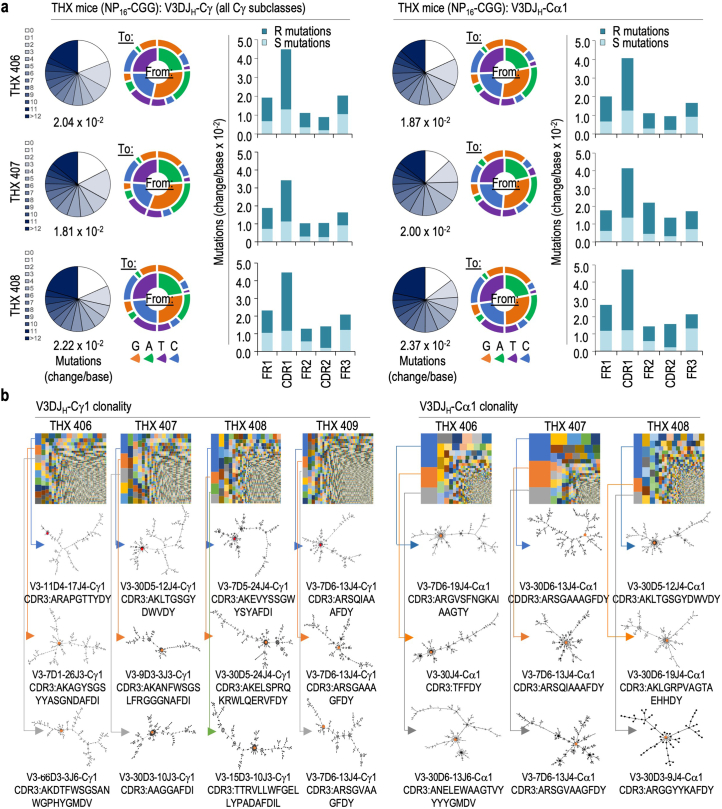
NP_16_-CGG-immunized THX mice mount a T-dependent class-switched antibody response to NP entailing select B cell oligoclonal expansion and SHM-mediated intraclonal diversification. **a, b**, Spleen huB cell huV3DJ_H_-Cγ and huV3DJ_H_-Cα1 transcripts in NP_16_-CGG-immunized THX mice (*n* = 3, same mice as in Fig. 4d–f) were analyzed for SHM, B cell clonal expansion and intraclonal diversification. (**a**) In the SHM pie charts, slices depict proportions of transcripts carrying given numbers of point-mutations; slice gray gradients depict increasing numbers of point-mutations; the overall mutation frequency (change/base) is listed below each pie chart. Spectrum of point-mutations depicted as donut charts. Means of total, S and R huV3 mutation frequencies in FR1, CDR1, FR2, CDR2 and FR3 of recombined huV3DJ_H_-Cγ and huV3DJ_H_-Cα1 transcripts depicted as histograms. (**b**) huV3DJ_H_-Cγ1 and huV3DJ_H_-Cα1 huB cell clones and intraclonal diversification, as depicted by TreeMaps and phylogenetic trees. Individual rectangle or square (unique color) area reflects huB cell clone size. In THX 406, 407, 408 and 409 mice, the 3 largest huV3DJ_H_-Cγ1 huB cell clones accounted for 3.5%, 6.9%, 8.3% and 4.6% of huV3DJ_H_-Cγ1 huB cells, while the 3 largest huV3DJ_H_-Cα1 huB cell clones accounted for 22.6%, 31.2% and 12.5% of huV3DJ_H_-Cα1 huB cells in THX 406, 407 and 408 mice. Select huIgG^+^B cell clones expressed V3 with V3-30 overutilization (over 24% of V3DJ_H_-Cγ1 transcripts). Intraclonal diversification is depicted for each of the three largest clones as a genealogical tree constructed based on shared and unique point-mutations in recombined huV3DJ_H_-Cγ1 and huV3DJ_H_-Cα1 transcripts.

Upon intraperitoneal (i.p.) immunization with T cell-dependent NP_16_-CGG conjugated hapten, THX mice showed serum huIgM at levels comparable to huNBSGW mice. They, however, made significantly greater amounts of total and high-affinity NP_4_-specific huIgG1, huIgG2, huIgG3, huIgA and huIgE than huNBSGW mice, with JAX NSG huCD34 mice making virtually no such antibodies (Fig. 4a). THX mice showed spleen huIgG^+^ and huIgA^+^ B cells, huCD27^+^CD38^+^ plasmablasts (PBs)/PCs, as accompanied by MZ huCD19^+^IgM^+^IgD^+^CD27^+^ B cells and including class-switched memory huCD19^+^huIgD^−^CD27^+^ and huCD19^+^IgG^+^CD27^+^ B cells^35^ at greater numbers than huNBSGW and JAX NSG huCD34 mice (Fig. 4b). Their B cells expressed higher levels of huAID and huBLIMP-1 than huNBSGW mice (Fig. 4c). THX 406, 407, 408 and 409 mouse huIgG^+^ and huIgA^+^ B cells accumulated more than 2.0 × 10^−2^ somatic point mutations per base, with a high ratio of replacement (R) to silent (S) mutations^36^ in huV1DJ_H_-Cγ, huV3DJ_H_-Cγ, huV1DJ_H_-Cα1 and huV3DJ_H_-Cα1 transcripts. In such THX mice, select huIgG^+^ B cell clones, expressing mainly V1 and V3 (including V3–V30) genes, expanded and intraclonally diversified, likely responding to NP_16_-CGG (Fig. 4d–f and Extended Data Fig. 4a,b)—NP_16_-specific huB cells sorted from THX mouse 406 included the two largest huV1DJ_H_-Cγ1 clones. Thus, THX mice can mount a mature T cell-dependent response, entailing B cell huAID and huBLIMP-1 expression, SHM/CSR, BCR-driven clonal selection and intraclonal diversification, differentiation of specific huPCs and huMBCs, yielding high-affinity antibodies and as accompanied by huMZ B cells.

### THX mice mount a T cell-independent specific and mature antibody response

**Extended Data Fig. 5:**
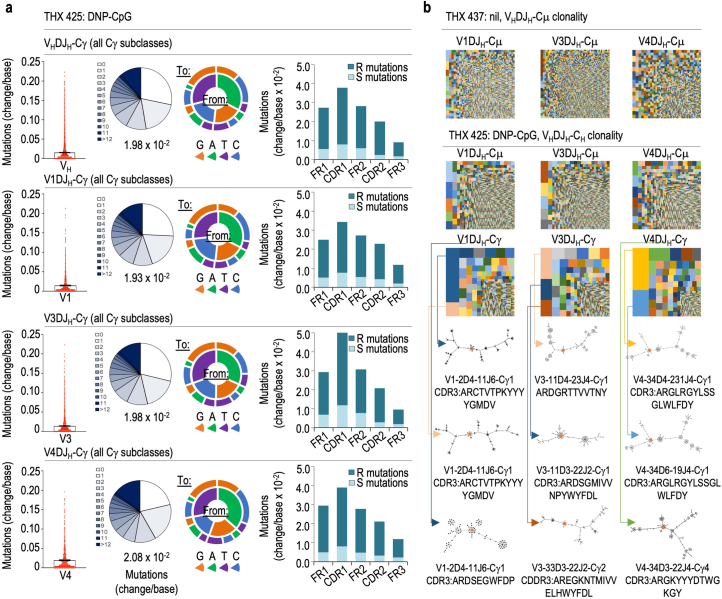
DNP-CpG-immunized THX mice mount a T-independent class-switched antibody response to DNP entailing select B cell oligoclonal expansion and SHM-mediated intraclonal diversification. **a**, Spleen huB cell huV_H_DJ_H_-Cγ transcripts in a DNP-CpG-immunized THX mouse (*n* = 1, THX 425 as in Fig. 4g) were analyzed for SHM. Left, huV_H_ mutation frequency (change/base) in recombined huV_H_DJ_H_-Cγ transcripts, as depicted by scatter plots. Each dot depicts a single sequence and bar depicts mean with s.e.m. Middle, in the SHM pie charts, slices depict proportions of transcripts carrying given numbers of point-mutations; slice gray gradients depict increasing numbers of point-mutations; the overall mutation frequency (change/base) is listed below each pie chart. Spectrum of point-mutations depicted as donut charts. Right, means of total, S and R huV_H_, huV1, huV3, huV4 mutation frequencies in FR1, CDR1, FR2, CDR2 and FR3 of recombined huV_H_DJ_H_-Cγ transcripts depicted as histograms. **b**, huV_H_DJ_H_-Cμ and huV_H_DJ_H_-Cγ huB cell clones and intraclonal diversification in a non-intentionally immunized THX mouse (*n* = 1, THX 437) and DNP-CpG-immunized THX mouse 425 as in (**a**), as depicted by TreeMaps and phylogenetic trees. Individual rectangle or square (unique color) area reflects huB cell clone size. In the DNP-CpG-immunized mouse (THX 425), the 3 largest huV1DJ_H_-Cμ, huV3DJ_H_-Cμ and huV4DJ_H_-Cμ huB cell clones accounted for 7.2%, 7.7% and 4.5% of huV1DJ_H_-Cμ, huV3DJ_H_-Cμ and huV4DJ_H_-Cμ huB cells, while only accounting for 2.1%, 1.4% and 1.4% of similar huB cells in the non-intentionally immunized THX mouse (THX 437). In the same DNP-CpG-immunized THX mouse, the 3 largest huV1DJ_H_-Cγ, huV3DJ_H_-Cγ and huV4DJ_H_-Cγ huB cell clones accounted for 22.3%, 16.8% and 29.4% of huV1DJ_H_-Cγ, huV3DJ_H_-Cγ and huV4DJ_H_-Cγ huB cells.

Mature T cell-independent antibody responses are mounted by *Tcrb*^−/−^*Tcrd*^−/−^ and NSG/B mice through B cell Toll-like receptors (TLRs)^37–39^. THX mice i.p. injected with T cell-independent TLR9 ligand DNP-CpG made greater amounts of total and high-affinity DNP_5_-specific huIgM, huIgG, huIgA and huIgE than huNBSGW mice, with JAX NSG huCD34 mice making few high-affinity antibodies (Fig. 4g). They showed greater numbers of spleen huIgG^+^ and huIgA^+^ B cells, class-switched memory huIgD^−^CD27^+^ B cells and huCD27^+^CD38^+^ PBs/PCs, together with more spleen and blood MZ huIgM^+^IgD^+^CD27^+^ B cells than huNBSGW or JAX NSG huCD34 mice (Fig. 4h–j). THX mice showed increased B cell huAID and huBLIMP-1 expression and DNP_5_-specific hugM, huIgG and huIgA antibody-secreting cells (ASCs) in spleen and BM than huNBSGW mice (Fig. 4k,l). They secreted huIgM, huIgD, huIgG and huIgA in the respiratory tract (bronchoalveolar lavage fluid, BALF) and showed huIgM-, huIgD- and huIgA-expressing B cells in intestinal lamina propria together with huCD3^+^ T cells (Fig. 4m,n). They also developed Peyer’s patches, not detected in huNBSGW mice, hosting MZ huCD19^+^IgM^+^IgD^+^CD27^+^ B cells, class-switched huCD19^+^IgG^+^ and huIgA^+^ B cells, GC huCD19^+^CD38^+^CD27^−^IgG^+^ and huCD19^+^CD38^+^CD27^–^IgA^+^ B cells, memory huCD19^+^CD27^+^IgD^−^B cells, huCD19^+^CD38^+^CD138^+^CD27^+^ PBs, huCD19^−^CD38^+^CD138^+^CD27^+^ PCs, huCD3^+^CD4^+^CD8^−^ and huCD3^+^CD4^−^CD8^+^ T cells, and huCD3^+^CD4^+^CXCR5^+^PD-1^+^ T_FH_ cells (Extended Data Fig. 2b). In THX mice, gut lymphoid cells were associated with high levels of fecal huIgD and huIgA (free and bound to fecal bacteria; Fig. 4o,p). THX mice huB cells accumulated somatic point mutations at more than 1.9 × 10^−2^ changes per base with high R:S mutation ratios, through select clonal expansion and intraclonal diversification of huV1DJ_H_-Cμ-, huV3DJ_H_-Cγ- and huV4DJ_H_-Cγ-expressing huB cells (Extended Data Fig. 5a,b). Thus, THX mice can mount a mature T cell-independent antibody response, entailing B cell huAID and huBLIMP-1 expression, SHM/CSR, huPC and huMBC differentiation, huB cell clonal selection and intraclonal diversification, yielding high-affinity antibodies. Also, unlike huNBSGW mice, they develop gut-associated Peyer’s patches containing MZ huB cells, huT cells, GC huIgG^+^ and huIgA^+^ B cells, memory huB cells and huPBs/huPCs. THX mice also display MZ huB cells in blood and spleen, secrete BALF huIgM, huIgD, huIgG, huIgA and fecal antibacterial huIgD and huIgA.

### huB cells from THX mice have full differentiation potential

**Extended Data Fig. 6:**
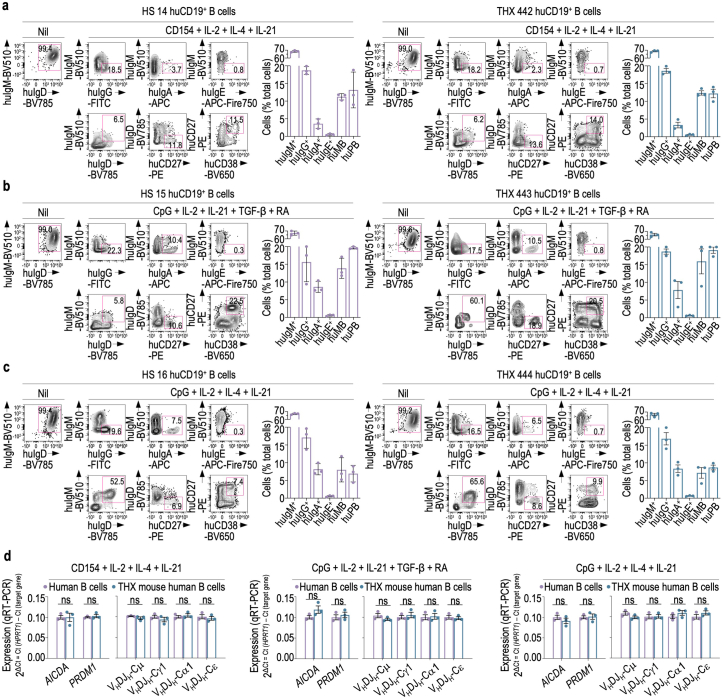
THX mice huB cells undergo CSR and differentiation as efficiently as huB cells from adult humans. **a**–**c**, Naïve huIgM^+^IgD^+^ B cells isolated from blood of healthy humans (*n* = 3, HS 14, 15, 16) and spleens of non-intentionally immunized THX mice (*n* = 3, THX 442, 443, 444) were cultured for 120 h upon stimulation with: (**a**) CD154 (3 U/ml), huIL-2 (100 ng/ml), huIL-4 (20 ng/ml) and huIL-21 (50 ng/ml), (**b**) CpG ODN2395 (2.5 mg/ml), huIL-2, huIL-21, TGF-β (4 ng/ml) and retinoic acid (4 ng/ml), or (**c**) CpG ODN2395, huIL-2, huIL-4 and huIL-21. Identification of huIgM^+^, huIgD^+^, huIgG^+^, huIgA^+^ or huIgE^+^ B cells, huCD27^+^IgD^–^ class-switched memory-like B cells (huMB) and huCD27^+^CD38^+^ PBs by flow cytometry. huCD45^+^CD19^+^ cells were pre-gated in all FACS analyses. **d**, *AICDA*, *PRDM1*, V_H_DJ_H_-Cμ, V_H_DJ_H_-Cγ1, V_H_DJ_H_-Cα1 and V_H_DJ_H_-Cε transcript expression in HS and THX mice huB cell microcultures (*n* = 3 biological replicates for each different microculture), as measured by qPCR and normalized to *HPRT1* expression (2^−ΔCt^ method). In histograms, each dot represents transcript expression from one human or one THX mouse huB cell microculture and the bar depicts the mean with s.e.m. Statistical significance (**d**) was assessed by two-sided Student’s unpaired *t-*test (NS, not significant). Source data

Naive huIgM^+^IgD^+^ B cells from THX mice and from humans were cultured in vitro to compare their potential to undergo CSR, PC and memory-like B cell differentiation. Upon culture with T cell-dependent (CD154, IL-2, IL-4 and IL-21) or T cell-independent (CpG, IL-2, IL-21, transforming growth factor (TGF)-β and retinoic acid, or CpG, IL-2, IL-4 and IL-21) stimuli, THX mouse huB cells underwent CSR to IgG, IgA and IgE, differentiated to huCD27^+^CD38^+^ PBs and class-switched huIgD^−^CD27^+^ memory-like huB cells like huB cells from humans, expressing comparable *AICDA*, *PRDM1* and post-recombination huV_H_DJ_H_-Cγ, huV_H_DJ_H_-Cα and huV_H_DJ_H_-Cε transcripts (Extended Data Fig. 6a–d).

### THX mice develop LNs, huTECs, huT_FH_ cells, generate huMBCs and form GCs

**Fig. 5:**
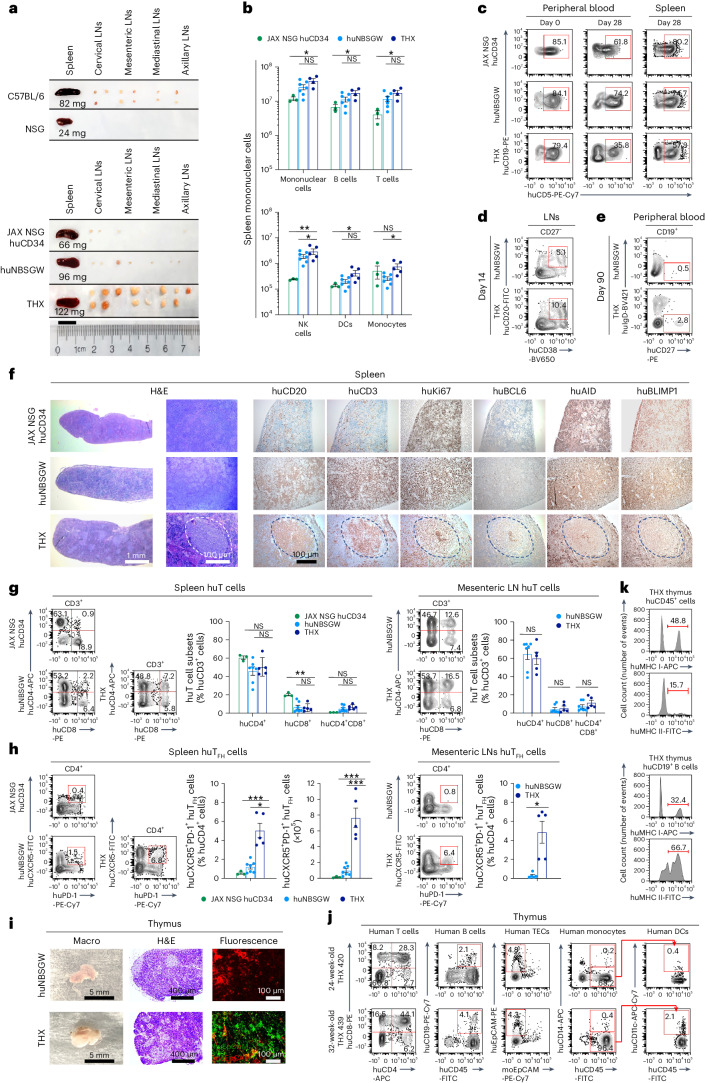
THX mice develop thymic huTECs and huB cells, differentiate huT_FH_ cells, form GCs and generate class-switched huMBCs. **a**–**k**, THX (*n* = 5), huNBSGW (*n* = 7) and JAX NSG huCD34 (*n* = 3) mice of the *n* = 7, 7 and 4 mice, respectively, of Fig. 4, were injected i.p. with NP_16_-CGG on day 0, boosted on day 14 and euthanized on day 28, unless otherwise specified. **a**, Spleen and LNs (cervical, mesenteric, mediastinal and axillary) of NP_16_-CGG-immunized THX, huNBSGW and JAX NSG huCD34 mice and non-immunized NSG and C57BL/6 mice (scale bar, 1 cm). **b**, Spleen huB cells, huT cells, huNK cells, huDCs and human monocytes in THX versus huNBSGW and JAX NSG huCD34 mice (39.5 ± 6.6 × 10^6^ versus 26.0 ± 4.4 × 10^6^ and 11.9 ± 1.5 × 10^6^ mononuclear cells). **c**, Blood and spleen huCD19^+^CD5^+^ (B1) cells in THX, huNBSGW and JAX NSG huCD34 mice on days 0 and 28. **d**,**e**, Mesenteric LN GC huCD27^−^CD20^+^CD38^+^ B cells (**d**, day 14) and blood class-switched memory huCD19^+^CD27^+^IgD^−^ B cells (**e**, day 90) in THX and huNBSGW mice. **f**, Spleen sections from THX, huNBSGW and JAX NSG huCD34 mice (day 14, hematoxylin and eosin (H&E) and immunohistochemical huCD20, huCD3, huKi67, huBCL6, huAID and huBLIMP-1; scale bars, 1.0 mm and 100 μm). **g**,**h**, huCD4^+^, huCD8^+^, huCD4^+^CD8^+^ T cells (**g**) and huCD4^+^CXCR5^+^PD-1^+^ T_FH_ cells (**h**) in spleens and mesenteric LNs of THX, huNBSGW and JAX NSG huCD34 mice. **i**, Thymus sections of THX and huNBSGW mice (whole organ, H&E, human and mouse EpCAM^+^ TECs immunofluorescence; scale bars, 100 μm, 400 µm and 5 mm). **j**,**k**, Human and mouse EpCAM^+^ TECs, huT cells, huB cells, huDCs, human monocytes (**j**) and cells expressing huMHC class I and huMHC class II (**k**) in THX mouse thymus. Images (**a**), micrographs (**f** and **i**) and FACS plots (**c**–**e**, **g**, **h**, **j** and **k**) are from one mouse per group, each representative of three mice. huCD45^+^ cells were pre-gated in all FACS analyses. In the histograms (**b**, **g** and **h**), each dot represents an individual mouse and bars depict the mean with s.e.m. Statistical significance (**b**, **g** and **h**) was assessed by two-sided Student’s unpaired *t*-test (NS, not significant; *P < 0.05, **P < 0.01, ***P < 0.001). Source data

Deficient peripheral lymphoid organ development, particularly LNs, has been an important limitation of humanized mice^5^. Unlike similarly immunized huNBSGW or JAX NSG huCD34 mice, NP_16_-CGG-immunized THX mice developed well-formed cervical, mediastinal, axillary and mesenteric LNs. They showed greater numbers of spleen huB cells, huT cells, huNK cells, huDCs and human monocytes (Fig. 5a,b). THX mice also showed an increased huCD5^−^ to huCD5^+^ B cell (B2/B1) ratio as compared to huNBSGW or JAX NSG huCD34 mice and accumulated more class-switched LN GC huCD20^+^CD38^+^CD27^−^ B cells and circulating memory huCD19^+^CD38^−^IgD^−^CD27^+^ B cells (Fig. 5c–e). Further, they developed GCs containing huCD20^+^ B cells, huCD3^+^ T cells, proliferating huKi67^+^ cells, huBCL6^+^ B cells, huAID^+^ B cells and huBLIMP-1^+^ PBs, while huNBSGW and JAX NSG huCD34 mice did not (Fig. 5f). In spleen and LNs, the proportions of huCD4^+^ T and huCD4^+^CD8^+^ T cells were comparable across the three humanized mouse models, while huCD8^+^ T cells were more numerous in JAX NSG huCD34 than THX or huNBSGW mice (Fig. 5g). Further, THX but not huNBSGW or JAX NSG huCD34 mice showed abundant huT_FH_ cells in spleen and mesenteric LNs (Fig. 5h). Unlike huNBSGW mice, THX mouse thymi showed medullary and cortical organization and abundant huTECs (huEpCAM^+^CD45^−^). They also showed huCD19^+^ B cells and huCD14^−^CD11c^+^ DCs, which together with huTECs mediate T cell selection^40–42^, as well as huCD14^+^ monocytes, which like huB and huT cells did not decrease with age (Fig. 5i,j). Finally, THX mouse thymus huCD45^+^ cells broadly expressed human major histocompatibility complex (huMHC) class I and/or huMHC class II (Fig. 5k). Thus, THX mice develop peripheral LNs and thymus-containing huT cells, huTECs, huB cells, huDCs and human monocytes, differentiate huT_FH_ and huB cells to form GCs, increase B2/B1 cell ratio and generate huMBCs.

### Flagellin-vaccinated THX mice mount a neutralizing response to *Salmonella*

**Fig. 6:**
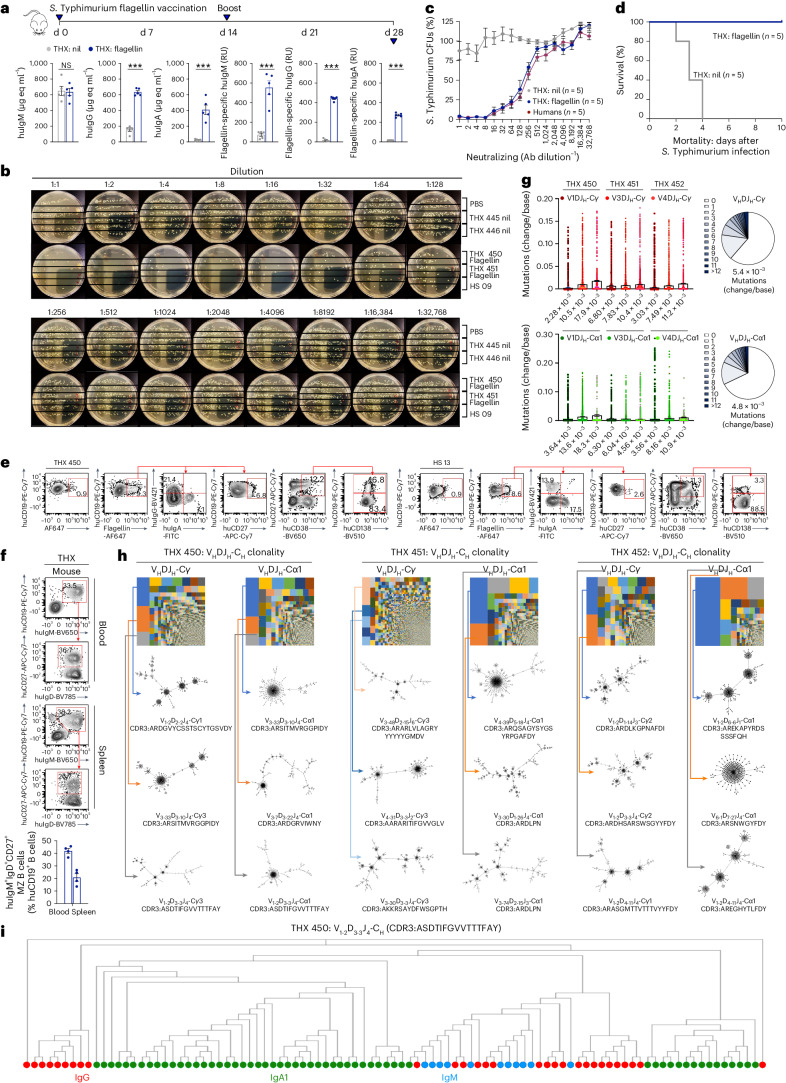
THX mice vaccinated with flagellin mount a mature neutralizing antibody response to *S*. Typhimurium. **a**–**i**, THX mice were injected i.p. with *S*. Typhimurium flagellin (50 μg in 100 μl alum) or nil (100 μl alum) on day 0, boosted (50 μg in 100 μl PBS or 100 μl PBS) on day 14 and euthanized on day 28. **a**, Total serum immunoglobulin concentration (μg eq ml^−1^) and flagellin-specific human antibodies (RUs) in flagellin-vaccinated (*n* = 5) and non-vaccinated (nil, *n* = 5) THX mice measured by specific ELISAs (NS, not significant; ***P < 0.001, two-sided Student’s unpaired *t*-test). **b**,**c**, Dose-dependent antibody *S*. Typhimurium neutralizing activity of sera from flagellin-vaccinated THX mice (*n* = 5), non*-*vaccinated THX mice (*n* = 5) and healthy humans (*n* = 5); representative Luria-Bertoni (LB)-agar plates showing (residual) *S*. Typhimurium colony-forming units (CFUs) at each serum dilution. **d**, Survival of flagellin-vaccinated (*n* = 5) and non-vaccinated (*n* = 5) THX mice infected orally with *S*. Typhimurium (1 × 10^5^ CFUs, day 21; Kaplan–Meier curves, *P* = 0.0026, log-rank Mantel–Cox test). **e**, Flagellin-specific huCD19^+^ B cells, huIgG^+^ B cells, huIgA^+^ B cells and class-switched memory huCD19^+^CD27^+^ B cells in flagellin-vaccinated THX mouse spleen and healthy human blood, as identified by binding of Andy Fluor 647 (AF647)-labeled flagellin (AF647 alone as negative control); identification of huCD19^+^CD138^+^ PBs and huCD19^−^CD138^+^ PCs among pre-gated huCD27^+^CD38^+^ cells. **f**, Blood and spleen MZ huCD19^+^IgM^+^IgD^+^CD27^+^ B cells (41.9% ± 1.9% and 20.7% ± 3.0% huB cells, respectively) in flagellin-vaccinated THX mice (*n* = 4). **g**, Point mutation frequencies (changes per base) in sorted spleen huB cell huV_H_DJ_H_-Cγ (V1: 4.0 ± 1.4 × 10^−3^, V2: 8.6 ± 0.9 × 10^−3^, V3: 1.3 ± 0.2 × 10^−2^) and huV_H_DJ_H_-Cα1 (V1: 4.5 ± 0.9 × 10^−3^, V3: 9.3 ± 2.3 × 10^−3^, V4: 1.1 ± 0.4 × 10^−2^) transcripts of additional flagellin-vaccinated THX mice (*n* = 3, THX 450, 451, 452) depicted as scatterplots and pie charts. Each dot represents a single sequence and bars depict the mean with s.e.m. **h**, huV_H_DJ_H_-Cγ and huV_H_DJ_H_-Cα1 huB cell clones and intraclonal diversification in THX mice as in **g**, depicted by TreeMaps and phylogenetic trees. Individual rectangle or square (unique color) area reflects huB cell clone size. In THX 450, 451 and 452 mice, the three largest huV_H_DJ_H_-Cγ and huV_H_DJ_H_-Cα1 clones accounted for 18.6%, 9.2% and 18.4% of huV_H_DJ_H_-Cγ huB cells and 19.6%, 33.4% and 57.9% of huV_H_DJ_H_-Cα1 huB cells. **i**, Evolutive lineage of a huB cell clone that underwent SHM and CSR in a flagellin-vaccinated THX mouse (THX 450). huCD45^+^ cells were pre-gated in all FACS analyses. In the histograms (**a** and **f**), each dot represents an individual mouse and bars depict the mean with s.e.m. Source data

**Extended Data Fig. 7:**
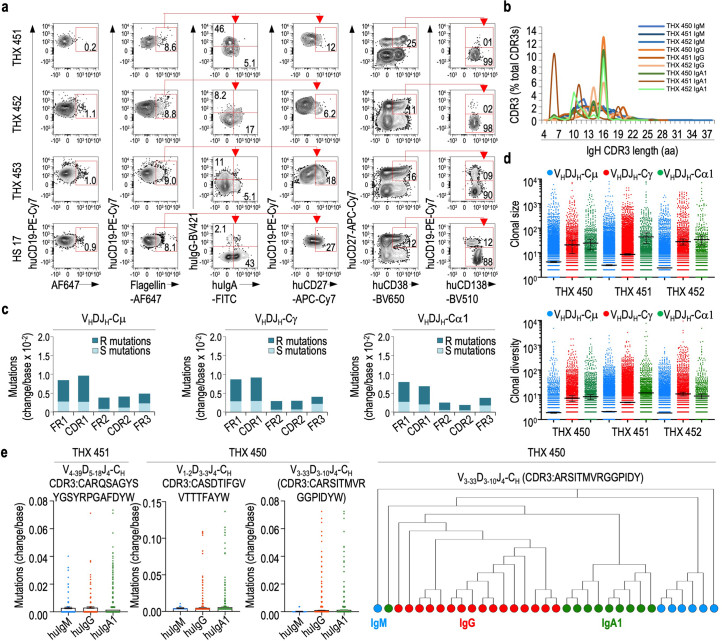
Flagellin-vaccinated THX mice mount a class-switched antibody response to *S*. Typhimurium entailing select B cell oligoclonal expansion and SHM-mediated intraclonal diversification. **a**–**e**, THX mice (*n* = 3, THX 450, 451, 452, same mice as in Fig. 6g, h) were injected i.p. with *S*. Typhimurium flagellin on day 0 (50 μg in 100 μl alum), boosted on day 14 (50 μg in 100 μl PBS) and euthanized on day 28. (**a**) Flagellin-specific huCD19^+^ B cells, huIgG^+^ B cells, huIgA^+^ B cells and class-switched memory huCD19^+^huCD27^+^ B cells in flagellin-vaccinated THX mice spleen and blood of a healthy human, as identified by binding of AF647-labeled flagellin (AF647 alone as negative control); identification of huCD19^+^CD138^+^ PBs and huCD19^—^CD138^+^ PCs among pre-gated huCD27^+^CD38^+^ cells. huCD45^+^ cells were pre-gated in all FACS analyses. **b**–**e**, Spleen huB cell huV_H_DJ_H_-C_H_ transcripts analyzed for CDR3 length, R:S mutations, huB cell clonal size and diversity, mutation frequency and evolution of a huB cell clone. (**b**) CDR3 length distribution in huIgM^+^, huIgG^+^ and huIgA^+^ B cells. Colors denote different antibody isotypes; color gradients denote different THX mice. (**c**) Means of total, S and R huV_H_ mutation frequencies in FR1, CDR1, FR2, CDR2 and FR3 of recombined huV_H_DJ_H_-C_H_ transcripts depicted as histograms. Data are from one THX mouse representative of 3 THX mice. (**d**) huV_H_DJ_H_-Cμ, huV_H_DJ_H_-Cγ and huV_H_DJ_H_-Cα1 huB cell clonal size and diversity in flagellin-vaccinated THX mice (*n* = 3, THX 450, 451, 452) depicted as scatter plots. Bars depict the mean with s.e.m. (**e**) Left, point-mutation frequency (change/base) in huB cell huV_H_DJ_H_-C_H_ transcripts of flagellin-vaccinated THX mice (*n* = 2, THX 450, 451) depicted as scatter plots. Each dot depicts a single sequence and the bar depicts the mean with s.e.m. Right, evolutive lineage of a huB cell clone that underwent SHM and CSR in a flagellin-vaccinated THX mouse (*n* = 1, THX 450). The root represents the rearranged, unmutated and unswitched recombined huV_3-33_D_3-10_J_4_-Cμ gene sequence of the huB cell progenitor and the leaves represent somatically hypermutated or class-switched and somatically hypermutated huB cell sub-mutants. Nodes represent huB cell sub-mutants that emerged during the clonal evolutionary process.

**Extended Data Fig. 8:**
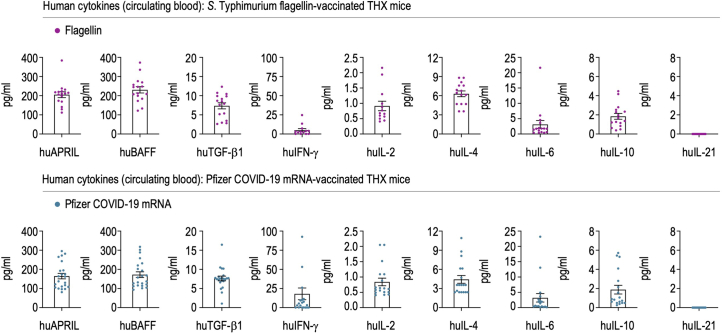
Serum human cytokine concentrations in flagellin- and COVID-19 mRNA-vaccinated THX mice. Serum huAPRIL, huBAFF, huTGF-β1, huIFN-γ, huIL-2, huIL-4, huIL-6, huIL-10 and huIL-21 (pg ml^−1^) in flagellin-vaccinated (*n* = 16) and Pfizer COVID-19 mRNA-vaccinated (*n* = 24) THX mice measured by Luminex Human Discovery Assay 8-plex or TGF-β Premixed Magnetic Luminex Performance Assay (R&D Systems Luminex Platform). In the histograms, each dot represents human cytokine concentration from an individual mouse and the bar depicts the mean with s.e.m. No significant difference was found in huAPRIL, huTGF-β1, huIFN-γ, huIL-2, huIL-6, huIL-10 and huIL-21 concentrations between flagellin-vaccinated and COVID-19 mRNA-vaccinated THX mice. huBAFF and huIL-4 concentrations were significantly greater in flagellin-vaccinated than in COVID-19 mRNA-vaccinated THX mice (*P* = 0.0134 and *P* = 0.0229, two-sided Student’s unpaired *t-*test). Flagellin-vaccinated THX mice showed blood incretion of huAPRIL, huBAFF, huTGF-β, huIFN-γ, huIL-2, huIL-4, huIL-6, huIL-10 and huIL-21 (205 ± 15.20, 231 ± 16.50, 7351 ± 794, 5.03 ± 1.94, 0.91 ± 0.16, 6.29 ± 0.45, 3.11 ± 1.30, 1.85 ± 0.30 and < 0.1 pg ml^−1^) at human physiological concentrations. COVID-19 mRNA-vaccinated THX mice also showed blood incretion of huAPRIL, huBAFF, huTGF-β, huIFN-γ, huIL-2, huIL-4, huIL-6, huIL-10 and huIL-21 (164 ± 14.45, 172 ± 14.86, 7627 ± 610, 18.00 ± 7.66, 0.84 ± 0.12, 4.43 ± 0.60, 3.06 ± 1.31, 1.89 ± 0.45 and < 0.1 pg ml^−1^, mean ± s.e.m.) at human physiological concentrations. In healthy adult humans, the approximate concentrations (range) of circulating cytokines are as follows: huAPRIL, 100–400 pg ml^−1^; huBAFF, 50–400 pg ml^−1^; huTGF-β1, 1000–10,000 pg ml^−1^; huIFN-γ, 0.1–4.2 pg ml^−1^; huIL-2, 0.1–2.0 pg ml^−1^; huIL-4, 0.5–4.0 pg ml^−1^; huIL-6, 0.1–5.0 pg ml^−1^; huIL-10, 0.1–2.8 pg ml^−1^; huIL-21, < 0.1 pg ml^−1^. In healthy humans, circulating huIL-21 is below 100 fg ml^−1^, a concentration below the detection limit of Luminex Human Discovery Assay 8-plex. It is important to note that human cytokine concentration ranges may vary depending on the type of assay used for measurement. The human cytokine concentration ranges reported here were derived from multiple sources^93–98^. Source data

THX mice vaccinated with purified *S*. Typhimurium flagellin made anti-flagellin huIgM, huIgG and huIgA, a *Salmonella*-neutralizing response comparable to humans, and survived *S*. Typhimurium infection, while non-vaccinated THX mice did not (Fig. 6a–d). Their bactericidal antibody response was accompanied by blood and spleen MZ huCD19^+^IgM^+^IgD^+^CD27^+^ B cells, flagellin-specific huCD19^+^IgG^+^ and huCD19^+^IgA^+^ B cells, huCD19^+^CD38^+^CD138^+^CD27^+^ PBs, CD19^−^CD38^+^CD138^+^CD27^+^ PCs and specific memory huCD19^+^CD27^+^ B cells, at higher frequencies than similar cells in humans. Flagellin-specific spleen huB cells sorted from THX mice expressed huV_H_DJ_H_-Cγ and huV_H_DJ_H_-Cα1 transcripts involving V1, V3 and V4 genes, pseudo-normal huIgH CDR3 lengths distribution, peaking at 16 amino acids, and bearing substantial loads of point mutations. huIgM^+^, huIgG^+^ and huIgA^+^ B cells underwent select clonal expansion and intraclonal diversification, with the three largest huV_H_DJ_H_-Cα1-expressing huB cell clones accounting for a greater proportion of huV_H_DJ_H_-Cα1-huB cells than the three largest huV_H_DJ_H_-Cγ-expressing huB cell clones did of huV_H_DJ_H_-Cγ-huB cells (Fig. 6e–i and Extended Data Fig. 7a–e). Also, vaccinated THX mice showed blood incretion of huAPRIL, huBAFF, huTGF-β, human interferon gamma (huIFN-γ), huIL-2, huIL-4, huIL-6, huIL-10 and huIL-21 at human physiological concentrations (Extended Data Fig. 8 and Supplementary Table 6). Thus, flagellin-vaccinated THX mice mount a protective antibody response to *Salmonella*, entailing SHM/CSR, huB cell clonal selection and intraclonal diversification, huPC and huMBC differentiation, huMZ B cells and blood incretion of antibody response-related human cytokines.

### COVID-19 mRNA-vaccinated THX mice mount an RBD-neutralizing response

**Fig. 7:**
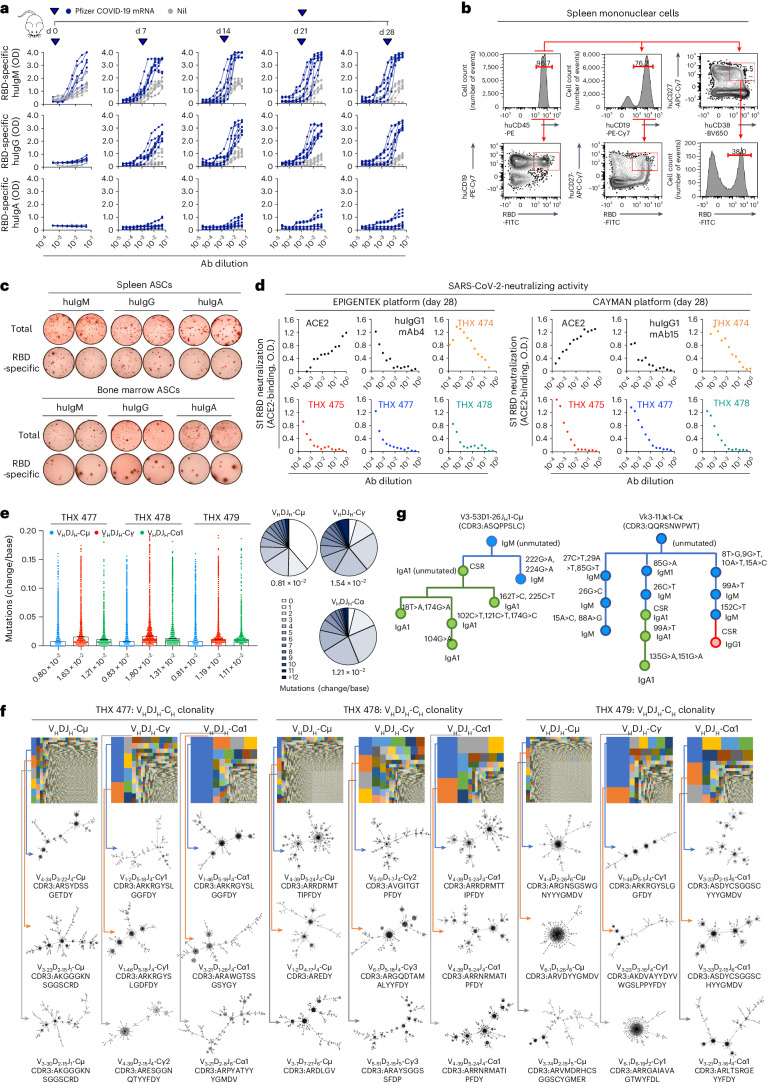
THX mice vaccinated with Pfizer COVID-19 mRNA mount a mature neutralizing antibody response to SARS-CoV-2 Spike S1 RBD. **a**–**g**, THX mice were injected i.m. with Pfizer-BioNTech 162b2 COVID-19 mRNA vaccine (5 μg in 50 μl PBS) or nil (50 μl PBS) on day 0, boosted (5 μg in 50 μl PBS or 50 μl PBS) on day 21 and euthanized on day 28. **a**, Serum RBD-specific human antibodies in COVID-19 mRNA-vaccinated (*n* = 8) and non-vaccinated (nil, *n* = 8) THX mice by specific ELISA (titers expressed as optical density (OD) readings at different dilutions). **b**, Total and RBD-specific spleen huCD45^+^ cells, huB cells, huMBCs and huPBs/PCs in COVID-19 mRNA-vaccinated THX mice, as identified by binding of labeled SARS-CoV-2 Spike S1 RBD. **c**, Total and RBD-specific huIgM, huIgG and huIgA ASCs in spleen and BM of COVID-19 mRNA-vaccinated THX mice, as analyzed by specific ELISPOTs. Data in **b** and **c** are from one THX mouse representative of three THX mice. **d**, Dose-dependent neutralizing antibody activity of sera from COVID-19 mRNA-vaccinated THX mice (*n* = 4), as analyzed by EpigenTek and Cayman SARS-CoV-2-neutralizing antibody detection platforms. SARS-CoV-2-neutralizing humAb4 and humAb15 were provided as positive control by EpigenTek and Cayman. **e**, Point mutation frequencies (changes per base) in spleen huB cell huV_H_DJ_H_-C_H_ (huV_H_DJ_H_-Cμ: 0.8 ± 0.01 × 10^−2^, huV_H_DJ_H_-Cγ: 1.5 ± 0.2 × 10^−2^, huV_H_DJ_H_-Cα1: 1.2 ± 0.1 × 10^−2^) transcripts of COVID-19 mRNA-vaccinated THX mice (*n* = 3, THX 477, 478, 479) depicted as scatterplots and pie charts. Each dot represents a single sequence and bars depict the mean with s.e.m. **f**, huV_H_DJ_H_-C_H_ B cell clones and intraclonal diversification in THX mice as in **e**, depicted by TreeMaps and phylogenetic trees. Individual rectangle or square (unique color) area reflects huB cell clone size. In THX 477, 478 and 479 mice, the three largest huV_H_DJ_H_-Cμ, huV_H_DJ_H_-Cγ and huV_H_DJ_H_-Cα1 clones accounted for 4.7%, 3.9% and 2.8% of huV_H_DJ_H_-Cμ huB cells, 22.0%, 16.1% and 36.9% of huV_H_DJ_H_-Cγ huB cells and 42.8%, 40.7% and 22.5% of huV_H_DJ_H_-Cα1 huB cells. **g**, Interplay of SHM and CSR shapes B cell stepwise intraclonal diversification in COVID-19 mRNA-vaccinated THX mice, as exemplified by genealogical trees outlining the evolution of two huB cell clones, one tracked from its huV3-53D1-26J_H_1-Cμ heavy-chain huB cell progenitor, the other from its huVκ3-11Jκ1-Cκ light-chain huB cell progenitor. Source data

**Extended Data Fig. 9:**
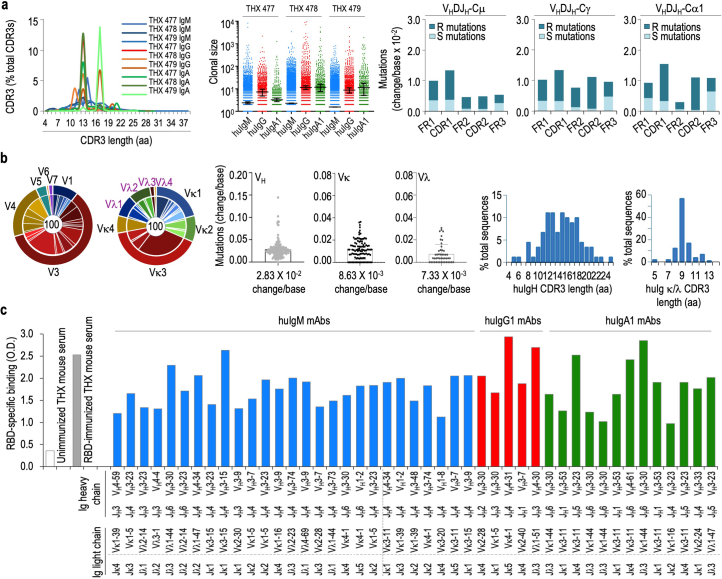
THX mice vaccinated with Pfizer-BioNTech 162b2 COVID-19 mRNA mount a class-switched and somatically hypermutated antibody response to SARS-CoV-2 Spike S1 RBD. **a**, Spleen huB cell huV_H_DJ_H_-Cμ, huV_H_DJ_H_-Cγ and huV_H_DJ_H_-Cα1 transcripts in COVID-19 mRNA-vaccinated THX mice (*n*=3, same mice as in Fig. 7e, f) were analyzed for CDR3 length, R:S mutations and huB cell clonal size. Left, CDR3 length distribution in recombined huV_H_DJ_H_-C_H_ transcripts. Colors denote different antibody isotypes; color gradients denote different THX mice. Middle, huV_H_DJ_H_-Cμ, huV_H_DJ_H_-Cγ and huV_H_DJ_H_-Cα1 huB cell clonal size depicted as scatter plots. Bars depict the mean with s.e.m. Right, means of total, S and R V_H_ mutation frequencies in FR1, CDR1, FR2, CDR2 and FR3 depicted as histograms. R:S data are from one THX mouse representative of 3 THX mice. **b–c**, Spleen RBD-specific huB cell huV_H_DJ_H_-C_H_, huVκJκ and huVλJλ transcripts in 3 additional COVID-19 mRNA-vaccinated THX mice were reverse transcribed and amplified by RT-PCR. Paired huV_H_DJ_H_ and huVκJκ or huVλJλ gene segments from 100 single cells were used to make recombinant human monoclonal antibodies. (**b**) Left, huV_H_, huVκ or huVλ gene family member expression in 100 recombinant human monoclonal antibodies, as depicted by pie charts. Colors depict different huV_H_, huVκ or huVλ gene families; color gradients denote individual gene family members. The 100 human monoclonal antibodies showed predominant utilization of V3, V4 and V1, together with Vκ3, Vκ1 and Vκ2 as well as Vλ1 and Vλ2 genes. Middle, mutation frequency (change/base) of recombined huIgH V_H_DJ_H_ and huIg VκJκ or VλJλ regions in recombinant human monoclonal antibodies, as depicted by scatter plots. Each dot represents a single sequence and the bar depicts the mean with s.e.m. Right, CDR3 length distribution in paired V_H_DJ_H_ and human immunoglobulin VκJκ or VλJλ human monoclonal antibodies. huIgH CDR3 lengths varied between 5 and 25 amino acids, peaking at 12, 13 and 15 amino acids; those of huVκ and huVλ varied between 5 and 13 amino acids, peaking at 9 amino acids. **c**, Forty-five expressed recombinant human monoclonal antibodies were selected for analysis of paired huIgH and huIgL genes based on their highest RBD-binding activity (> 1.0 OD by specific ELISA). Shown are 27 huIgM (blue), 5 huIgG (red) and 13 huIgA (green) monoclonal antibodies. Consistent with the higher haploid representation of V3, V4 and V1 gene families, V3 genes were the most frequently utilized (35 human monoclonal antibodies), particularly V3-23 (10 human monoclonal antibodies), V3-30 (8), V3-9 (4) and V3-7 (3), followed by V4 (7) and V1 (3) genes. Twenty-two human monoclonal antibodies utilized J_H_4, 9 J_H_6 and 6 J_H_3, with J_H_1, J_H_5 and J_H_2 accounting for the remaining 8 human monoclonal antibodies. Thirty-five human monoclonal antibodies utilized Vκ genes, with Vκ3-11 as the most frequently utilized (8 human monoclonal antibodies) followed by Vκ1-44 (4) and Vκ4-1 (4). Ten human monoclonal antibodies utilized Vλ genes, with Vλ1-44 and Vλ2-14 (2 and 2 human monoclonal antibodies) as the most frequently utilized. Source data

THX mice mounted a mature antiviral response. THX mice vaccinated intramuscularly (i.m.) with Pfizer-BioNTech 162b2 coronavirus disease 2019 (COVID*-*19) mRNA, according to human vaccination schedule, made huIgM, huIgG and, to a moderate degree, huIgA to SARS-CoV-2 Spike S1 RBD (37 amino acid core peptide) as well as RBD-specific huASCs, huCD19^+^ B cells, memory huCD19^+^CD27^+^ B cells and huCD19^+^CD27^+^CD38^+^ PBs (Fig. 7a–c). They showed blood incretion of huAPRIL, huBAFF, huTGF-β, huIFN-γ, huIL-2, huIL-4, huIL-6, huIL-10 and huIL-21 at human physiological concentrations (Extended Data Fig. 8 and Supplementary Table 7). THX mice sera with high RBD-binding huIgG titers displayed SARS-CoV-2-neutralizing activity comparable to huIgG1 monoclonal antibodies, as assessed by two different Spike S1 RBD–ACE2 platforms (Cayman Chemical and EpigenTek; Fig. 7d). In vaccinated THX mice, huB cell huV_H_DJ_H_-C_H_ transcripts displayed huIgH CDR3 lengths peaking at 13 and 17 amino acids and substantial loads of V gene somatic point mutations, greater in huV_H_DJ_H_-Cγ and huV_H_DJ_H_-Cα1 than in huV_H_DJ_H_-Cμ transcripts, all with high R:S mutation ratios (Fig. 7e and Extended Data Fig. 9a). huIgG^+^, huIgA^+^ and huIgM^+^ B cells underwent select clonal expansion and intraclonal diversification, with the three largest huV_H_DJ_H_-Cγ- and huV_H_DJ_H_-Cα1-expressing huB cell clones accounting for a greater proportion of huV_H_DJ_H_-Cγ-huB and huV_H_DJ_H_-Cα1-huB cells than the three largest huV_H_DJ_H_-Cμ-expressing huB cell clones did of huV_H_DJ_H_-Cμ-huB cells (Fig. 7f and Extended Data Fig. 9a). In fact, the latter comprised a multitude of huIgM^+^ B cell ‘microclones’, likely not participants in the anti-RBD response. The interplay of SHM and CSR in shaping B cell intraclonal diversification was exemplified by genealogical trees outlining the stepwise evolution of two clones, one developing from an unmutated huV3-53D1-26J_H_1-Cμ-B cell progenitor, the other from an unmutated huVκ3-11 Jκ1-Cκ-B cell progenitor (Fig. 7g). RBD-specific huB cells were sorted from spleens of additional mRNA-vaccinated THX mice, and paired huV_H_DJ_H_-Cγ and huVκJκ or huVλJλ gene segments were amplified from single huB cells to make 100 recombinant human monoclonal antibodies. These showed predominant utilization of human V3, V4 and V1, reflecting the human haplotypic representation of these human V_H_ genes, together with human Vκ3, Vκ1 and Vκ2 as well as Vλ1 and Vλ2 genes; as expected, somatic point mutations were more frequent in V_H_ than in Vκ or Vλ gene segments (Extended Data Fig. 9b). Forty-five of the 100 human monoclonal antibody huB cell clones (27 huIgM, 5 huIgG1 and 13 huIgA1) were selected based on greater RBD-binding activity and characterized for paired huIgH and huIgL genes (Extended Data Fig. 9c). Thus, upon COVID*-*19 mRNA vaccination, THX mice mount a mature neutralizing antibody response to Spike S1 RBD, entailing SHM/CSR, huB cell select clonal expansion and intraclonal diversification, huPC differentiation, generation of huMBCs and blood incretion of antibody response-related human cytokines.

### RBD–KLH-vaccinated THX mice mount a mature antibody response to RBD

**Extended Data Fig. 10:**
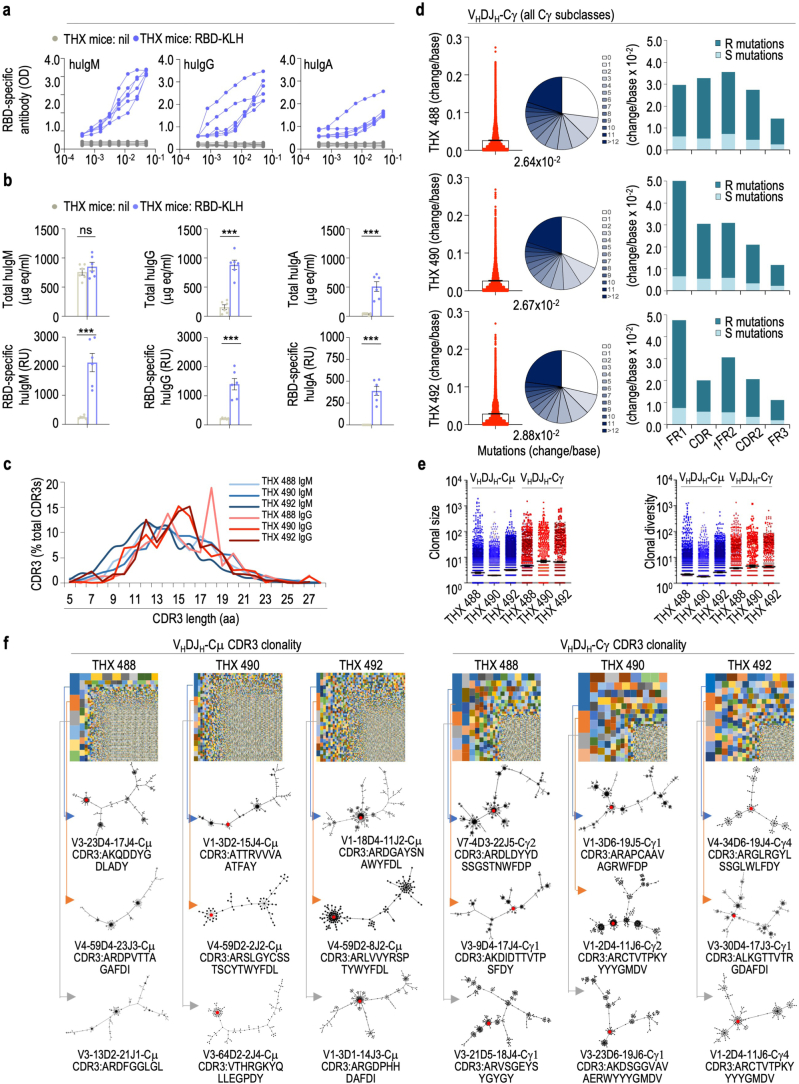
RBD-KLH-vaccinated THX mice mount a class-switched and somatically hypermutated antibody response to SARS-CoV-2 Spike S1 RBD. **a, b**, THX mice were injected i.p. with RBD-KLH (100 μg in 100 μl alum) or nil (100 μl alum) on day 0, boosted (100 μg in 100 µl PBS or 100 μl PBS alone) on day 21 and euthanized on day 28. Total serum human immunoglobulin and RBD-specific huIgM, huIgG and huIgA antibodies in RBD-KLH-immunized (*n*=6) and non-immunized (*n*=6) THX mice measured by specific ELISAs (total human immunoglobulin concentrations expressed as μg eq ml^−1^ and RBD-specific human antibody titers as OD readings at different dilutions or RUs). In the histograms, each dot represents an individual mouse and the bar depicts the mean with s.e.m. Statistical significance was assessed by two-sided Student’s unpaired *t*-test (NS, not significant; **P<0.01, ***P<0.001). **c–f**, Spleen huB cell huV_H_DJ_H_-C_H_ transcripts in RBD-KLH-immunized THX mice (*n*=3 of the 6 as in **a–b**, THX 488, 490, 492) were analyzed for CDR3 length, clonal expansion and intraclonal diversification. (**c**) CDR3 length distribution in huV_H_DJ_H_-C_H_ transcripts. Colors denote different antibody isotypes; color gradients denote different THX mice. (**d**) huV_H_ mutation frequency (change/base) in huV_H_DJ_H_-Cγ (2.7+0.08x10^-2^, mean+s.e.m.) transcripts depicted as scatter plots (left) and pie charts (middle). Each dot represents a single sequence and the bar depicts the mean with s.e.m. Right, means of total, S and R huV_H_ mutation frequencies in FR1, CDR1, FR2, CDR2 and FR3 of huV_H_DJ_H_-Cμ and huV_H_DJ_H_-Cγ transcripts depicted as histograms. R:S data are from one THX mouse representative of 3 THX mice. (**e**) huV_H_DJ_H_-Cμ and huV_H_DJ_H_-Cγ huB cell clonal size and diversity in RBD-KLH-immunized THX mice (THX 488, 490, 492) depicted as scatter plots. Bars depict the mean with s.e.m. (**f**) huV_H_DJ_H_-Cμ and huV_H_DJ_H_-Cγ huB cell clones and intraclonal diversification, as depicted by TreeMaps and phylogenetic trees. Individual rectangle or square (unique color) area reflects huB cell clone size. In THX mice 488, 490 and 492, the 20 largest huV_H_DJ_H_-Cμ and huV_H_DJ_H_-Cγ huB cell clones accounted for about one-tenth of huV_H_DJ_H_-Cμ huB cells and one-fourth of huV_H_DJ_H_-Cγ huB cells. Source data

THX mice mounted a mature antibody response to SARS-CoV-2 Spike S1 RBD, as elicited by RBD (47 amino acid peptide containing a core of 37 amino acids) conjugated to keyhole limpet hemocyanin (KLH; i.p. priming and boost). RBD–KLH-injected THX mice, not non-vaccinated controls, made specific huIgM, huIgG and, to a lesser extent, huIgA antibodies to RBD (37 amino acid core peptide) (Extended Data Fig. 10a,b). Their huB cell huV_H_DJ_H_-Cμ and huV_H_DJ_H_-Cγ transcripts displayed heterogeneous huIgH CDR3 lengths and heavy loads of somatic point mutations with high R:S mutation ratios (Extended Data Fig. 10c,d)—huV_H_DJ_H_-Cγ-huB cells underwent greater select clonal expansion and intraclonal diversification than huV_H_DJ_H_-Cμ-huB cells, which comprised a multitude of ‘microclones’, reflecting moderate to no clonal expansion (Extended Data Fig. 10e,f). Thus, upon vaccination with SARS-CoV-2 Spike S1 RBD–KLH, THX mice mount a specific mature antibody response to RBD, involving SHM/CSR, huB cell clonal selection and intraclonal diversification.

### THX mice can model SLE autoimmunity

**Fig. 8:**
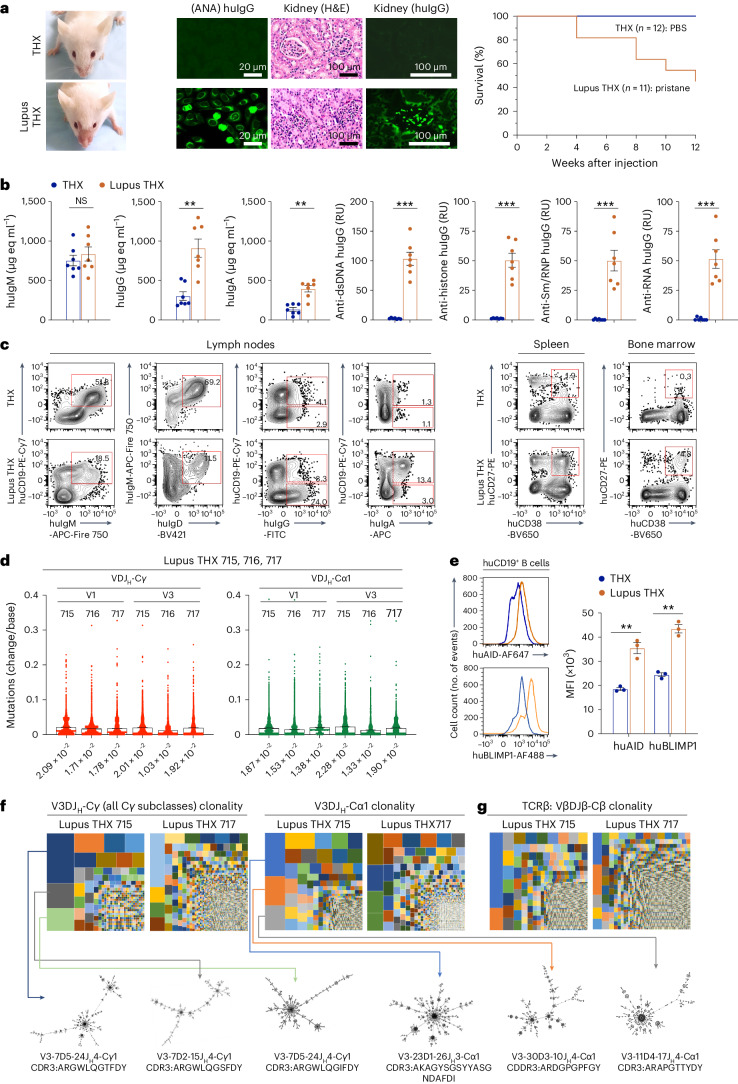
THX mice can develop human autoantibodies and model SLE. **a**–**g**, Lupus THX mice were generated by injecting THX mice (*n* = 11) once i.p. with 500 μl pristane. THX mice (*n* = 12) injected with 500 μl PBS served as healthy controls. **a**, Left, malar rash in a (huNSGW41-derived) Lupus THX mouse (3 weeks after pristane injection). Middle, serum antinuclear IgGs (scale bar, 20 μm) and kidney immunopathology (H&E and anti-huIgG immunofluorescence; scale bar, 100 μm) in Lupus THX and THX mice (12 weeks after pristane or PBS injection). Micrographs are from one Lupus THX and one THX mouse, each representative of 3 mice. Right, survival of Lupus THX (*n* = 11) and THX (*n* = 12) mice through 12 weeks after pristane or PBS injection (Kaplan–Meier curves, *P* = 0.04, log-rank Mantel–Cox test). **b**, Total serum huIgM, huIgG and huIgA (µg eq ml^−1^) as well as anti-dsDNA, anti-histone, anti-RNP and anti-RNA huIgG (RUs) in Lupus THX (*n* = 7) and THX (*n* = 7) mice measured by specific ELISAs. **c**, Mesenteric LN huIgM^+^IgD^+^, huIgM^+^, huIgG^+^ and huIgA^+^ B cells as well as spleen and BM huCD27^+^CD38^+^ PBs/PCs in Lupus THX and THX mice. Data are from one Lupus THX and one THX mouse, each representative of three mice (3 Lupus THX mice were euthanized when showing obvious signs of disease; 3 euthanized healthy THX mice entered into the study in addition to the 12 followed in the survival study). **d**, Numbers of point mutations in recombined huV_H_DJ_H_-Cγ and huV_H_DJ_H_-Cα1 transcripts in Lupus THX mice (*n* = 3, Lupus THX 715, 716, 717) huB cells depicted as scatterplots. Each dot represents a single sequence and bars depict the mean with s.e.m. **e**, Spleen huB cell intracellular AID and BLIMP-1 expression in Lupus THX (*n* = 3) and THX (*n* = 3) mice. **f**,**g**, huV3DJ_H_-Cγ and huV3DJ_H_-Cα1 huB cell clones and intraclonal diversification (**f**) as well as huVβDJβ-Cβ huT cell clones (**g**) in Lupus THX mice (*n* = 2, Lupus THX 715, 717), as depicted by TreeMaps and phylogenetic trees. Individual rectangle or square (unique color) area reflects huB or huT cell clone size. Analyses in **b**–**g** were performed at 6 weeks after pristane or PBS injection. huCD45^+^ cells were pre-gated in all FACS analyses. In the histograms (**b** and **e**), each dot represents an individual mouse and bars depict the mean with s.e.m. Statistical significance (**b** and **e**) was assessed by two-sided Student’s unpaired *t*-test (NS, not significant; **P < 0.01, ***P < 0.001). Source data

Pristane, a saturated terpenoid alkane with pro-inflammatory activity, can induce lupus-like autoimmunity in C57BL/6, BALB/c and γ-irradiated humanized NSG mice^43^. Male and female 18-week-old THX mice (generated from huNBSGW and huNSGW41 mice) were injected i.p. with pristane or PBS. As early as 3 weeks after pristane injection, THX mice developed a malar rash evocative of the ‘butterfly rash’ in individuals with systemic lupus erythematosus (SLE), concomitant with rising levels of serum huIgG and huIgA, including antinuclear, anti-dsDNA, anti-histone, anti-Sm/anti-RNP and anti-RNA huIgG autoantibodies, eventually leading to kidney glomerular huIgG deposition and immunopathology (Fig. 8a,b). As compared to THX mice, ‘Lupus THX’ mice showed fewer mesenteric LN huCD19^+^IgM^+^ and huCD19^+^IgD^+^ B cells, greater class-switched huCD19^+^IgG^+^ and huCD19^+^IgA^+^ B cells as well as spleen and BM huPCs (Fig. 8c). Lupus THX mouse huB cells accumulated somatic point mutations in huV_H_DJ_H_-Cγ and huV_H_DJ_H_-Cα1 transcripts and expressed higher levels of huAID and huBLIMP-1 than THX mice (Fig. 8d,e). Likely reflecting an ongoing antigen-driven process, huIgG^+^ and huIgA1^+^ B cells selectively expanded and intraclonally diversified, as exemplified by the largest huV3DJ_H_-Cγ- and huV3DJ_H_-Cα1-huB cell clones emerging from unmutated progenitors (Fig. 8f). Diversified huVβDJβ cells also underwent select clonal expansions (Fig. 8g). Finally, Lupus THX mice suffered 45% mortality at 12 weeks after pristane injection, contrasting with 100% survival of THX mice (Fig. 8a). Thus, THX mice are amenable to model human SLE, including huB and huT cell clonality, autoantibodies to nuclear components and kidney immunopathology leading to reduced lifespan.

## Discussion

Humanized mice have been constructed using BM, fetal liver or umbilical cord blood huCD34^+^ cells or PBMCs, with cord blood being a highly enriched source of HSCs^5^. In THX mice, cord blood huCD34^+^ cell engraftment of genetically myeloablated *Kit*^W-41J^ mice enables human cell multilineage development and full immune tolerance. Intracardiac injection would maximize huCD34^+^ cell dissemination to multifocal BM sites, thereby facilitating huCD45^+^ cell colonization of peripheral lymphoid organs, such as LNs, gut-associated lymphoid tissue and Peyer’s patches. In THX mice, this is promoted by E2 and contrasts with underdeveloped peripheral lymphoid formations in huNBSGW, JAX NSG huCD34 and other humanized mice^2–6^. In these, LN development could be achieved only by supraphysiological expression of transgenic murine thymic stromal cell-derived lymphopoietin^44^. Thus, in addition to neonatal grafting of *Kit*^W-41J^ immunodeficient mice by intracardiac injection, the innovative estrogen conditioning is critical to the making of THX mice.

An important limitation of humanized mouse models is the failure to mount mature antibody responses^2–6^. E2 support of antibody response maturation is consistent with stronger antibody responses to viral vaccines, such as SARS-CoV-2 virus, influenza and hepatitis B virus, or bacterial vaccines, such as diphtheria, tetanus and pneumococcus, and greater incidence of autoantibody-mediated autoimmunity in female than male mice and humans^21,22,45–47^. Accordingly, E2 promotes differentiation of virtually all immune cells, including B cells, T cells and granulocytes, all of which express ERα and ERβ^14,20–22,25,26^. Although more information is needed on E2 impact on HSC differentiation, CD34^+^ HSCs express ERα and ERβ (encoded by *Esr1* and *Esr2* genes) and engraft more efficiently in immunodeficient female than male mice^13–15,24,48^.

The comparable blood E2 levels in male and female THX mice were higher than in huNBSGW mice, but well within women’s E2 physiological range. The critical role of E2 in promoting B cell differentiation in THX mice likely reflects an intrinsic B cell estrogen activity^16,18,49^, as revealed by the THX mouse mature antibody response to T cell-independent DNP-CpG. In THX mice, E2 is critical in promoting development of LNs, Peyer’s patches and GCs, supporting differentiation of huTECs, huT_FH_ cells and huGC B cells, increasing B2:B1 cell ratio and generating huMBCs. E2 conditioning was also important for the appearance of huIgM, huIgD, huIgG and huIgA in BALF and feces, as well as the high baseline levels of huIgD, huIgG and huIgA in non-intentionally immunized THX mice. Additionally, E2 supported differentiation of huMZ B cells, which contribute antibodies that provide the first-line of defense against blood-borne microbial pathogens. Spleen huMZ B cells in THX mice, whether immunized with NP-CGG, DNP-CpG or *Salmonella* flagellin, were comparable, as a proportion of B cells, to spleen MZ B cells in humans and mice^50^. As in humans, THX mice huMZ B cells occurred at a greater proportion in circulating blood than spleen.

E2 induces a genetic program, including *Ptpn6, Bcl2* and *Vcam1* expression, that promotes B cell activation and survival, while dampening pro-apoptotic mediators, such as PD-1 (ref. ^16^). The direct impact of estrogen on B cell differentiation was reflected in the ability of huB cells from THX mice to undergo CSR, PC and memory-like B cell differentiation in vitro as efficiently as B cells from healthy humans, in response to T cell-dependent and T cell-independent stimuli. Indeed, E2 promotes B cell AID expression and SHM/CSR by upregulating HoxC4, a transcription factor that induces the *Aicda* promoter to activate this gene^27–29^. E2 also downregulates miR-26a, a most abundant microRNA in B cells and suppressor of *Aicda* transcription, thereby further promoting AID expression^30^. Additionally, estrogen response elements are clustered within IgH switch (S) regions^51^, potentially enabling E2 amplification of CSR. Once bound to estrogen response elements, ERα forms complexes with GATA3 and PBX1 co-transcription factors and other ERα immune cell function agonists, including NF-κB, AP-1 and C/EBPβ, leading to increased RNA polymerase II recruitment^14^. High estrogen:androgen ratios support differentiation of class-switched MBCs and PCs, as in human aromatase transgenic male mice^52^. By contrast, progesterone (P4), the most important progestogen, precursor of testosterone and potent agonist of nuclear progesterone receptor, exerts a negative activity on B cell proliferation, differentiation and *Aicda* expression, thereby dampening SHM/CSR^53,54^. P4 impact on B cells can reduce antibody-mediated defense and promote disease, such as in P4-treated female mice infected with influenza virus^55^. Like P4, testosterone would exert a negative impact on immune cell activities, thereby contributing to weaker antibody responses to bacterial and viral vaccines in men than women^21,22,45–47^.

THX mouse human antibody responses to T cell-dependent and T cell-independent conjugated haptens, *Salmonella* flagellin and viral SARS-CoV-2 Spike S1 RBD peptide, entailed SHM/CSR mediating intraclonal diversification of selectively expanded huIgG^+^ and huIgA^+^ B cell clones, whose sizes accounted for major proportions of their respective huIgG^+^ and huIgA^+^ B cell repertoires. This contrasted with the, generally, multitude of huIgM^+^ B cells with virtually no clonal expansion, possibly progenitors of expanded class-switched and somatically hypermutated huIgG^+^ and huIgA^+^ B cell clones. In COVID-19 mRNA- or flagellin-vaccinated THX mice, the lower level of circulating anti-RBD or anti-flagellin huIgA than huIgG was incongruous with the comparable huIgA^+^ and huIgG^+^ B cell clonal expansions, huIgA and huIgG mutational loads and huIgA and huIgG ASC numbers. It, however, is consistent with the lower level of anti-RBD huIgA than huIgG in blood and saliva of COVID-19 mRNA-vaccinated humans^56,57^ as well as the lower level of anti-flagellin huIgA than huIgG in humans infected with *Salmonella*^58^. The predominant V3, V4 and V1 gene utilization by the class-switched antibodies in COVID-19 mRNA-vaccinated THX mice is evocative of similar V gene utilization by the class-switched antibody response in COVID-19 mRNA-vaccinated humans^59^. The mutational load of greater than 10^−2^ changes per base in huB cell huV_H_DJ_H_-Cγ transcripts in COVID-19 mRNA- and RBD–KLH-vaccinated THX mice is also evocative of the heavy mutational load of COVID-19 mRNA vaccine-induced huIgG response in humans^59–61^, possibly reflecting the high immunogenicity of Spike S1 RBD^62,63^.

Humanized mice generally lack thymic huMHCs, resulting in huT cells selected on mouse MHC, a shortcoming corrected by grafting human thymus fragments, as in BLT mice^5^. THX mouse mature antibody responses induced by NP_16_-CGG, *Salmonella* flagellin and Pfizer COVID-19 mRNA were presumably dependent on CD4^+^ T cells educated on huTECs or other human cells expressing MHC class II^40–42^, such as huB cells and huDCs, also present in THX mouse thymus. But how could THX mice populate their thymus with huTECs, which supposedly emerge from non-hematopoietic CD34^−^ progenitors? In fact, epithelial cells can differentiate from CD34^+^ stem cells, including cord blood CD34^+^ cells^64–66^, possibly giving rise to huTECs. Interestingly, TECs express ERα and ERβ, consistent with an E2 role in promoting their differentiation^67^.

THX mouse maturation of antibody response involved blood incretion of huAPRIL and huBAFF at human physiological concentrations. APRIL supports B cell proliferation, CSR and PC differentiation, while BAFF supports immature B cell survival, B cell differentiation and antibody production^68^. Flagellin-vaccinated and Pfizer COVID-19 mRNA-vaccinated THX mice displayed comparable concentrations of blood huAPRIL. The former, however, showed higher levels of circulating huBAFF, likely reflecting flagellin induction of this B cell cytokine^69^. In THX mice, huAPRIL and huBAFF occurred together with huTGF-β, huIFN-γ, huIL-2, huIL-4, huIL-6 and huIL-10, all at human physiological levels and, possibly, as promoted by ERα signaling^14,19,20,49^. THX mouse physiological levels of human B cell growth factors and cytokines contrast with the generally dysregulated levels of knock-in or transgenic growth factors and cytokines in other immunized mice^5^, as exemplified by the supraphysiological expression of human granulocyte-macrophage colony-stimulating factor (huGM-CSF) and huIL-3 in huNSG-SGM3 mice, huGM-CSF, huIL-3 and huIL-6 in huMISTRG(6) mice or huBAFF (*TNFS13B)* in huBAFFKI mice^70^.

A shortcoming of humanized mice has been the lack of GCs, contributing to impaired antibody responses^5^. In THX mice, E2 supports differentiation of huT_FH_ cells, which make cytokines, such as IL-4, IL-6, IL-10 and IL-21, and critically promote GC huB cell differentiation, GC formation, BCR affinity maturation and generation of PCs and MBCs^71,72^. E2 promotes expansion of T_FH_ cells via PPARγ, thereby supporting the class-switched antibody response^49,73^. In activated huPBMCs, E2 increases not only PD-1^+^CXCR5^+^ T_FH_ but also ICOS^+^ T_FH_ cells, both important for GC formation^49,72^. In addition, E2 enhances expression of CXCR4 and CXCR5, which are central to GC dark and light zone organization as well as T cell homing by modulating expression of T cell chemokine receptors, such as CCR5 (refs. ^49,74^). Finally, E2 increases CD4^+^ T cell CD154 expression^22^ and upregulates EZH2 histone methyltransferase, which helps T_FH_ cell differentiation^75^.

Another shortcoming of humanized mice is poor development of human myeloid cells, particularly neutrophils^5^. Expression of huGM-CSF and huIL-3 in γ-radiation myeloablated humanized NSG-SGM3 and MISTRG mice as well as additional expression of human granulocyte colony-stimulating factor (hG-CSF), as in humanized MISTRGGR mice, has partially corrected human myeloid cell underrepresentation^5,76^. Neutrophils express both ERα and ERβ^20,25,26^, and estrogen has been shown to increase neutrophils in women’s peripheral blood and in mouse blood, BM and spleen^25^. THX mice reconstituted human neutrophils, to almost one-fourth of spleen huCD45^+^ cells, a proportion comparable to neutrophils in spleen of humans^31^. Finally, human platelets in THX mice accounted for approximately one-third of total platelets, possibly also as a result of direct E2 impact on megakaryocytes, which express ERα and ERβ and whose maturation is boosted by estrogen^77^.

The THX mouse gut microbiome, which consisted of *Muribaculaceae* and other bacterial families found in humans, profoundly differed from NBSGW mice microbiome, which was dominated by the exquisite ‘murine’ *Rikenellaceae*. By contrast, it shared bacteria, including the dominant *Muribaculaceae*, with huNBSGW mice, which, possibly reflecting the lack of E2 conditioning, also harbored remnants of *Rikenellaceae*, not found in THX mice. The human-like gut microbiome together with free and bacteria-bound fecal huIgD and huIgA, likely induced by microbial stimulation of gut lymphoid cells’ TLRs^38,39,78^, suggests that THX mice are suited to model human intestinal mucosa antibody responses. Nevertheless, further investigation is needed to elucidate the mechanisms underpinning E2 contribution to shaping the THX mouse microbiome in gut and airways and, possibly, the potential E2 contribution to support huILCs and peripheral resident T cells, both important in mucosal homoeostasis and defense.

Lupus murine models, such as MRL/*lpr* and genetically modified *Sle1, Sle2* and *Sle3* mice, all share a nonhuman immune system, mediating an autoantibody response that does not faithfully reproduce that of individuals with SLE. Estrogen plays a role in accelerating mouse lupus autoimmunity and may play a role in the development of human lupus^16,17,19,23,43,79^. E2 enhances anti-dsDNA antibody production in lupus huB cells and ERα accelerates lupus development in autoimmune (NZBxNZW)F1 mice in a B cell-intrinsic fashion^17,20,79^. Consistent with B cell clonal expansion in individuals with lupus, Lupus THX mice expanded and intraclonally diversified select huIgG^+^ and huIgA^+^ B cells and made class-switched autoantibodies to cell nuclear components, eventually leading to lupus-like symptoms and immunopathology. By overcoming limitations posed by the differences between mouse and human lupus^43^, Lupus THX mice would lend themselves to testing novel therapeutic approaches with immediate translatability to individuals with lupus. They would also provide a first proof-of-concept of THX mice modeling human disease.

Thus, THX mice achieve sustained human immune system reconstitution and express huBCR and huTCR repertoires as diverse as those of humans. They unveil and leverage a critical estrogen activity to promote human immune cell differentiation as well as maturation of human antibody and autoantibody responses. The mechanisms by which E2 supports these processes and incretion of relevant human cytokines remain to be defined in further detail, as do potential E2 long-term side-effects^16,52^, which, however, were not observed in THX mice. Thus, by overcoming the limitations of current humanized mouse models, THX mice provide an advanced and powerful platform for in vivo studies of human immune responses, particularly, antibody and autoantibody responses, for development of human vaccines and immune therapeutics, including modulators of unwanted human antibody responses.

## Methods

### Mice

C57BL/6J (RRID: IMSR_JAX: 000664), NSG (NOD.Cg-*Prkdc*^scid^*Il2rg*^tm1Wjl^/SzJ, RRID: IMSR_JAX: 005557)^8^, NBSGW (NOD.Cg-*Kit*^W-41J^*Tyr*^+^*Prkdc*^scid^*Il2rg*^tm1Wjl^/ThomJ, RRID: IMSR_JAX:026622)^12^, NSGW41 (NOD.Cg-*Kit*^W-41J^*Prkdc*^scid^*Il2rg*^tm1Wjl^/WaskJ, RRID: IMSR_JAX:026497)^11^ and JAX NSG huCD34 (RRID: IMSR_JAX: 005557) mice (Supplementary Table 8) were purchased from The Jackson Laboratory (JAX NSG huCD34 mice were constructed by grafting γ-irradiated 3-week-old female NSG mice with cord blood huCD34^+^ HSCs). In all experiments, male and female mice were used in virtually equal proportions.

huCD34^+^ HSCs to be used for construction of THX, huNBSGW, huNSGW41 and huNSG mice were isolated from human umbilical cord blood collected immediately after cesarean section from full-term, normally developed male and female newborns (in approximately equal numbers) upon informed consent from healthy puerperae (18–45 years old with no infectious disease or history of cancer) of different ages, races and ethnic backgrounds (Supplementary Table 9; Department of Obstetrics and Gynecology, The University of Texas Long School of Medicine). CD34^+^ cells were purified using EasySep Human CD34 Positive Selection Kit II (17856, STEMCELL Technologies) according to the manufacturer’s instructions, yielding at least 99% huCD34^+^ cell preparations. Freshly purified huCD34^+^ cells were resuspended in PBS supplemented with 2% FBS for immediate grafting or frozen in 10% dimethylsulfoxide, 72% FBS, 18% RPMI medium and kept in liquid nitrogen for later grafting.

huNSG mice (Supplementary Table 10) were constructed by myeloablative conditioning of NSG mice neonates (within 48 h of birth) with (1 Gy) γ-radiation, followed by intracardiac (left ventricle) injection with purified cord blood huCD34^+^ cells (1.5 × 10^5^ freshly isolated or frozen-thawed huCD34^+^cells in 50 μl PBS supplemented with 2.0% FBS) using a 27-gauge needle. huNBSGW (Supplementary Table 11) and huNSGW41 mice were constructed by grafting non-γ-irradiated, genetically myeloablated NBSGW and NSGW41 mice neonates (within 48 h of birth) intracardially (left ventricle) with cord blood huCD34^+^ cells. THX mice were generated by feeding huNBSGW or huNSGW41 mice E2 (3301, Sigma-Aldrich; 1.5 μM in drinking water resulting in a dose of 6.1 × 10^−4^ mg per kg body weight per day) ad libitum starting at 14–18 weeks of age (18 weeks in most cases) and continuing thereafter. After 4 weeks of E2 conditioning, huNBSGW or NSGW41 mice (referred to as THX mice; Supplementary Table 12) were ready for experiments or continued E2 for later use. E2 conditioning of huNBSGW or huNSGW41 mice did not start before 14 weeks of age, as estrogen (albeit at high dose) might inhibit early thymus development by T cell proliferation.

Most THX mice were constructed using NBSGW mice as only a dozen NSGW41 mice were acquired in 2019 from The Jackson Laboratory before the sale of such mice was discontinued. NSGW41-based THX mice were used in the human antibody response to NP-CGG (*n* = 3), in the lupus studies as part of the healthy THX controls (*n* = 4 of 12) as well as for generation of Lupus THX mice (*n* = 5) as described in ‘Lupus THX mice, human autoantibodies, immunopathology and mortality’. huCD45^+^ cells in blood, spleen and BM of humanized mice were identified by flow cytometry using APC-anti-huCD45 monoclonal antibody (304011, BioLegend; 1:100 dilution) and Pacific Blue-anti-moCD45 monoclonal antibody (103125, BioLegend; 1:100 dilution). Generally, THX and huNBSGW mice displayed up to 96.1% and 89.3% huCD45^+^ cells in circulating blood, respectively. huNSG and JAX NSG huCD34 mice displayed, at peak, approximately 45% and 20% huCD45^+^ cells, respectively. Circulating huCD45^+^ mononuclear cell numbers (cells per ml of blood) were measured by complete blood count analysis, in which blood was collected in EDTA-coated microtubes and analyzed using a XT2000iV or XE-5000 blood analyzer (Sysmex). THX, huNBSGW and huNSG mice used in all experiments were 20 to 24 weeks of age, unless indicated otherwise. JAX NSG huCD34 mice were 23 weeks of age. Mice used in all experiments were housed in a pathogen-free barrier animal vivarium facility at The University of Texas Health Science Center at San Antonio and were free of infection or disease. Housing rooms were maintained at a 14-h light/10-h dark cycle and controlled temperature of approximately 22–23 °C with 40–60% humidity. Food (Teklad LM-485 Sterilizable Mouse/Rat Diet, 7912, Inotiv) and water were sterilized.

### Estrogen

Serum estradiol concentrations in non-intentionally immunized THX and huNBSGW mice (18–24 weeks old) were measured using Cayman Estradiol ELISA Kit (501890, Cayman Chemical), according to the manufacturer’s instructions, and compared to mice and human physiological range^80–86^. This platform uses an estradiol acetylcholinesterase conjugate (estradiol acetylcholinesterase Tracer) in an inhibition/competition assay, measuring serum estradiol concentration by OD at 414 nm. High OD readings reflect low estradiol concentrations, while low OD readings reflect high concentrations. Sera were collected from equal numbers of male and female mice, with female mice sera collected generally during proestrus, metestrus and diestrus.

### FACS and CyTOF

For the cell surface FACS analysis, cells from blood of healthy humans or blood, BM (tibia and femur), thymus, spleen, LNs (cervical, mediastinal, axillary, mesenteric) and/or Peyer’s patches of humanized mice (THX, huNBSGW, huNSG or JAX NSG huCD34 mice) were surface stained with fluorochrome-conjugated monoclonal antibodies (Supplementary Table 13) in Hank’s Buffered Salt Solution (HBSS, MT21022CM, Fisher Scientific) plus 0.1% bovine serum albumin (BSA, BP1600-100, Fisher Scientific; BSA-HBSS) for 20 min. After washing, cells were resuspended in BSA-HBSS for flow cytometry. In vitro*-*stimulated and/or ex vivo mononuclear cells were stained with FITC-anti-huCD45 monoclonal antibody (clone 30-F11, 368507, BioLegend; 1:100 dilution), PE-anti-huCD45 monoclonal antibody (clone 2D1, 368509, BioLegend; 1:100 dilution), Pacific Blue-anti-moCD45 monoclonal antibody (clone 2D1, 103125, BioLegend; 1:100 dilution), PE-anti-huCD19 monoclonal antibody (clone HIB19, 302208, BioLegend; 1:100 dilution), PE-Cyanine7-anti-huCD19 monoclonal antibody (clone HIB19, 302216, BioLegend; 1:100 dilution), FITC-anti-huCD20 monoclonal antibody (clone 2H7, 302303, BioLegend; 1:100 dilution), BV510-anti-huCD138 monoclonal antibody (clone MI15, 356517, BioLegend; 1:100 dilution), PE-anti-huIgM monoclonal antibody (clone MHM-88, 314507, BioLegend; 1:100 dilution), BV510-anti-huIgM monoclonal antibody (clone MHM-88, 314521, BioLegend; 1:100 dilution), BV650-anti-huIgM monoclonal antibody (clone MHM-88, 314525, BioLegend; 1:100 dilution), APC-Fire 750-anti-huIgM monoclonal antibody (clone MHM-88, 314545, BioLegend; 1:100 dilution), BV421-anti-huIgD monoclonal antibody (clone IA6-2, 348225, BioLegend; 1:100 dilution), BV785-anti-huIgD monoclonal antibody (clone IA6-2, 348241, BioLegend; 1:100 dilution), BV421-anti-huIgG monoclonal antibody (clone M1310G05, 410703, BioLegend; 1:100 dilution), FITC-anti-huIgG monoclonal antibody (clone G18-145, 555786, BD Pharmingen; 1:100 dilution), APC-anti-huIgA monoclonal antibody (clone IS11-8E10, 130-113-427, Miltenyi Biotec; 1:50 dilution), FITC-anti-huIgA (c31577, Invitrogen; 1:100 dilution), APC-Fire 750-anti-huIgE monoclonal antibody (clone MHE-18, 325515, BioLegend; 1:100 dilution), APC-Cyanine7-anti-huCD11c monoclonal antibody (clone Bu15, 337217, BioLegend; 1:100 dilution), APC-anti-huCD14 monoclonal antibody (clone 63D3, 367117, BioLegend; 1:100 dilution), BV786-anti-huCD56 monoclonal antibody (clone 5.1H11, 362549, Biolegend; 1:100 dilution), PE-anti-huCD27 monoclonal antibody (clone M-T271, 356405, BioLegend; 1:100 dilution), BV650-anti-huCD38 monoclonal antibody (clone HB-7, 356619, BioLegend; 1:100 dilution), PE-Cyanine7-anti-huCD5 monoclonal antibody (clone UCHT2, 300621, BioLegend; 1:100 dilution), Super Bright 600-anti-huCD3 monoclonal antibody (clone OKT3, 63003741, eBioscience; 1:100 dilution), APC-anti-huCD4 monoclonal antibody (clone A161A1, 357407, BioLegend; 1:100 dilution), BV421-anti-huCD4 monoclonal antibody (clone A161A1, 357423, BioLegend; 1:100 dilution), PE-anti-huCD8 monoclonal antibody (clone SK1, 344705, BioLegend; 1:100 dilution), Alexa Fluor 700-anti-huCD8 monoclonal antibody (clone SK1, 344723, BioLegend; 1:100 dilution), PE-anti-huCXCR5 monoclonal antibody (clone J252D4, 356903, BioLegend; 1:100 dilution), FITC-anti-huCXCR5 monoclonal antibody (clone J252D4, 356913, BioLegend; 1:100 dilution), FITC-anti-huPD-1 monoclonal antibody (clone NAT105, 367411, BioLegend; 1:100 dilution), PE-Cyanine7-anti-huPD-1 monoclonal antibody (clone A17188B, 621615, BioLegend; 1:100 dilution), Pacific Blue-anti-huICOS monoclonal antibody (clone C398.4A, 313521, BioLegend; 1:100 dilution), PE-anti-huEpCAM (clone EPR20532-225, ab237397, Abcam; 1:100 dilution), PE-Cyanine7-anti-moEpCAM (clone G8.8, 118216, BioLegend; 1:100 dilution), APC-anti-huHLA-A,B,C (MHC I) monoclonal antibody (clone W6/32, 311409, BioLegend; 1:100 dilution), FITC-anti-huHLA-DR, DP, DQ (MHC II) monoclonal antibody (clone Tü39, 361705, BioLegend; 1:100 dilution) or 7-AAD (A9400, Sigma-Aldrich). For analysis of human red blood cells and platelets, THX mice red blood cells were stained with APC-anti-moTER-119 monoclonal antibody (clone TER-119, 116211, BioLegend; 1:100 dilution) and FITC-anti-huCD235a monoclonal antibody (clone HI264, 349103, BioLegend; 1:100 dilution). THX mice (low forward scatter) platelets were stained with PE-Cyanine7-anti-moCD41 monoclonal antibody (clone MWReg30, 133915, BioLegend; 1:100 dilution) and PerCp-anti-huCD61 monoclonal antibody (clone VI-PL2, 336409, BioLegend; 1:100 dilution).

For the intracellular FACS analysis, AID-expressing and BLIMP-1-expressing huB cells and huPBs/PCs (2.0 × 10^6^ cells) were surface stained with anti-huCD45, anti-huCD19, anti-huCD27, anti-huCD38 and anti-huCD138 monoclonal antibodies, as well as Fixable Viability Dye eFluor 780 (65-0865-14, Fisher Scientific). After washing, cells were fixed in Cytofix/Cytoperm buffer (554655, BD Biosciences, 250 µl) and incubated at 4 °C for 45 min. Cells were washed twice in BD Perm/Wash buffer (554723, BD Biosciences) for permeabilization and stained with Alexa Fluor 647–anti-huAID antibody (bs-7855R-FITC, Bioss; 1:50 dilution) or Alexa Fluor 488-anti-huBLIMP-1 monoclonal antibody (clone 646702, IC36081G, R&D Systems; 1:50 dilution) in BD Perm/Wash buffer for 30 min at 4 °C. Cells were washed again twice in BD Perm/Wash buffer and resuspended in BSA-HBSS for flow cytometry.

Flow cytometry analysis and sorting were performed using single-cell suspensions. Cells were gated by forward and side scattering to exclude dead cells and debris (Supplementary Fig. 1a–c). Cell analysis was performed on pre-gated huCD45^+^ cells using a BD LSR-II or FACS Celesta flow cytometer (BD Biosciences) with FACSDiva software v9.4 (BD Biosciences). Data were acquired and analyzed using FlowJo v10.9 (Tree Star).

To assess human immune lymphoid and myeloid cell reconstitution in THX mice, single-cell suspensions of splenic white cells from non-intentionally immunized THX mice (20–24 weeks old) were incubated for 30 min at 4 °C with a 50 μl cocktail of metal conjugated anti-human monoclonal antibodies (Supplementary Table 2) from the MaxPar Direct Immune Profiling Assay, 30 Marker Kit (201325, Fluidigm), followed by washing for 10 min at room temperature. Cell viability was measured by DNA intercalation (Cell-ID Intercalator-103Rh). Labeled cells were analyzed by Helios mass cytometer (CyTOF software v6.7, Fluidigm) using a flow rate of 0.045 ml min^−1^. Human immune lymphoid and myeloid cell population frequencies, quality-control metrics and data plot displays were acquired using Maxpar Pathsetter software v3.0 (401018, Fluidigm).

Bacteria-bound huIgD and huIgA in THX and huNBSGW mice were detected as we described^38,78^. Briefly, feces (10 mg) were suspended in 100 μl PBS, homogenized and centrifuged at 400*g* for 5 min to remove large particles. Supernatant was centrifuged at 8,000*g* for 10 min, then analyzed for free huIgD and huIgA by ELISA. To detect bacteria-bound huIgD and huIgA, the bacterial pellet was resuspended in 1 ml PBS containing 1.0% (wt/vol) BSA. After fixation with 7.2% formaldehyde for 10 min at room temperature, bacteria were washed with PBS, stained with FITC-anti-huIgD (clone IA6-2, 348205, BioLegend; 1:100 dilution) or APC-anti-huIgA (clone IS11-8E10, 130-113-427, Miltenyi Biotec; 1:50 dilution) monoclonal antibodies on ice for 30 min, washed again then resuspended in PBS containing 0.2 μg ml^−1^ DAPI for flow cytometry analysis. All events revealed by DAPI were considered as bacteria.

### Human mononuclear cells

huPBMCs were isolated from buffy coats obtained from healthy male and female human donors of different ages (18–65 years old), races and ethnic backgrounds (South Texas Blood and Tissue Center; Supplementary Table 14). The buffy coat was diluted at a 1:2 ratio in sterile PBS (pH 7.4, BP3991, Fisher Scientific) and then applied to a Histopaque-1077 density gradient (10771, Sigma-Aldrich) in 50 ml SepMate tubes (85450, STEMCELL Technologies), which were spun at 1,000*g* for 10 min. Recovered huPBMCs were washed in PBS and resuspended in RPMI (10-040-CV, Corning RPMI-1640 medium) supplemented with 10% vol/vol Hyclone FBS (42Q7980K, Gibco) and 1% vol/vol antibiotic-antimycotic solution (penicillin–streptomycin and amphotericin B, SV30079.01, Cytiva Life Sciences; FBS-RPMI).

Human immune cells were isolated from humanized mouse blood, BM, thymus, spleen, LNs and/or Peyer’s patches, and suspended in ACK Lysis Buffer (BP10-548E, Lonza) to lyse erythrocytes. Peripheral blood (approximately 250 μl) was collected from the submandibular vein into microtubes containing heparin (H19, Fisher Scientific; 25 μl, 1,000 units per ml). After quenching with FBS-RPMI and centrifugation, erythrocyte-free cells were resuspended in FBS-RPMI for further preparation or analysis.

### Differentiation of naive human B cells from humans and from THX mice

To analyze CSR, PC and MBC differentiation, naive huCD19^+^IgM^+^IgD^+^B cells were isolated from huPBMCs obtained from healthy participants by negative selection using EasySep Human Naive B Cell Isolation Kit (17254, STEMCELL Technologies), according to the manufacturer’s instructions, yielding at least 98% huCD19^+^IgM^+^IgD^+^B cells. After pelleting, huB cells were resuspended in FBS-RPMI before further analysis or stimulation. Naive huCD19^+^IgM^+^IgD^+^B cells were isolated from THX mouse spleens by negative selection using biotin-anti-huCD43 (9620-08, clone DF-T1, SouthernBiotech; 1:50 dilution) and biotin-anti-huCD3 monoclonal antibodies (300403, clone UCHT1, BioLegend; 1:50 dilution) followed by positive selection using biotin-anti-huIgD monoclonal antibody (348212, clone IA6-2, BioLegend; Supplementary Table 15) and MagniSort Streptavidin Positive Selection Beads (MSPB-6003-74, Thermo Fisher Scientific), yielding at least 98% huCD19^+^IgM^+^IgD^+^B cells. After pelleting, B cells were resuspended in FBS-RPMI. Naive huIgM^+^IgD^+^B cells from humans or THX mice were cultured in FBS-RPMI (5.0 × 10^5^ cells per ml) for 72 h (for RNA transcript analysis) or up to 120 h (for flow cytometry analysis) upon stimulation with: membrane-CD154 (3.0 U ml^−1^)^38,39,78^ or CpG ODN 2395 (Eurofins Genomics, 2.5 μg ml^−1^) plus recombinant huIL-2 (589102, BioLegend, 100 ng ml^−1^), recombinant huIL-4 (574002, BioLegend, 20 ng ml^−1^) and recombinant huIL-21 (571202, BioLegend, 50 ng ml^−1^) for CSR to huIgG. For CSR to huIgA, naive huIgM^+^IgD^+^ B cells were cultured under similar conditions upon stimulation with membrane-CD154 or CpG ODN 2395 plus recombinant huIL-2, recombinant huIL-21, recombinant TGF-β (781802, BioLegend, 4.0 ng ml^−1^) and recombinant retinoic acid (11017, Cayman Chemicals, 4.0 ng ml^−1^). Pre-gated huCD45^+^huCD19^+^ cells were stained with specific human monoclonal antibodies (Supplementary Table 13) to detect huIgM^+^, huIgD^+^, huIgG^+^, huIgA^+^ or huIgE^+^ B cells, huCD27^+^CD38^+^ PBs and class-switched huCD27^+^IgD^−^ memory-like B cells by flow cytometry.

### huBCR IgM^+^ B cell and huTCR repertoires and huIgM^+^ B and T cell clonality

To analyze expressed huV_H_DJ_H_-Cμ, huVκJκ-Cκ and huVλJλ-Cλ or huVαJα-Cα and huVβJβ-Cβ gene repertoires, huIgM^+^ B cells and huT cells were isolated from blood of healthy humans (Supplementary Table 14) and spleens of non-intentionally immunized THX mice (20–24 weeks old). RNA (2 µg) was extracted using RNeasy Mini Kit (74104, Qiagen). huV_H_DJ_H_-Cμ, huVκJκ-Cκ and huVλJλ-Cλ or huVαJα-Cα and huVβJβ-Cβ mRNA transcripts were reverse transcribed from huIgM^+^ B or huT cell RNA by RT-5′ RACE PCR using SuperScript III First-Strand Synthesis System (18080051, Invitrogen) and a huCμ-, huCκ-, huCλ-, huCα- or huCβ-specific reverse primer (Supplementary Table 16). Single-strand cDNA was cleaned up using QIAquick PCR purification kit (28104, QIAGEN) and 3′ poly-dA tailed by TdT and dATP. The dA-tailed cDNA was then amplified by PCR using a forward oligo-dT primer together with a nested huCμ-, huCκ-, huCλ-, huCα- or huCβ-specific reverse primer. Both forward and reverse primers were tagged with Illumina overhang adaptors. PCR amplification conditions were 95 °C for 30 s, 55 °C for 30 s and 72 °C for 40 s for 35 cycles. cDNA amplicons were cleaned up using QIAquick PCR purification kit, further amplified by index PCR involving Illumina clustering adaptors and beads cleanup, quantified and then loaded onto the Illumina MiSeq system using the 300-bp pair-end sequencing module. huV_H_DJ_H_-Cμ, huVκJκ-Cκ, huVλJλ-Cλ, huVαJα-Cα and huVβJβ-Cβ repertoires were analyzed using IMGT/HighV-QUEST v1.9.2 (The International ImMunoGeneTics Information System; http://www.imgt.org/HighV-QUEST/home.action/).

To identify individual huB and huT cell clones and analyze huB or huT cell clonal diversity, huB cell V_H_DJ_H_-Cµ or huT cell VβDJβ-Cβ transcripts (up to 250,000 sequences) of healthy humans and THX mice were analyzed by Illumina MiSeq amplicon sequencing and segregated based on the same huV_H_ or huVβ gene segment, the same and unique huIgH or huTCRβ CDR3 together with the same huJ_H_ or huVβ sequence^87–91^. Each discrete clone was depicted as an individual rectangle or square (unique color), whose area reflects huB or huT cell clone size, as inferred from the sum of identical huV_H_DJ_H_-Cµ or huVβDJβ-Cβ transcripts (TreeMaps, Microsoft Excel v16.83 and IMGT/HighV-QUEST statistic data).

### THX mice huB cell SHM/CSR, clonality and intraclonal diversification

To analyze SHM in the NP_16_-CGG-induced antibody response, RNA (2 µg) was extracted from THX mice total and sorted NP_16_-specific huB cells using the RNeasy Mini Kit (74104, Qiagen), and cDNA was synthesized using the SuperScript III First-Strand Synthesis System (18080051, Invitrogen) with oligo-dT primer. Rearranged huV1DJ_H_-Cγ, huV3DJ_H_-Cγ, huV1DJ_H_-Cα1 and huV3DJ_H_-Cα1 cDNA was amplified using a huV1 or huV3 leader-specific forward primer together with a nested huCγ- or huCα-specific reverse primer tagged with Illumina overhang adaptors (Supplementary Table 16) and Phusion high-fidelity DNA polymerase (M0530S, New England BioLabs)—amplification of huIgH V1 and V3 genes was chosen as these families include gene members of high sequence similarity to mouse V1-72 (V186.2/V3 gene), the gene encoding the most efficient ‘NP-binding’ mouse IgH V segment (https://www.imgt.org/ligmdb/view?id=J00239/)^36,92^. PCR amplification conditions were 98 °C for 10 s, 60 °C for 45 s and 72 °C for 1 min for 30 cycles. The cDNA amplicons were further amplified and sequenced as described in ‘huBCR IgM^+^ B cell and huTCR repertoires and huIgM^+^ B and T cell clonality’. Somatic point mutations in recombined transcripts were analyzed using IMGT/HighV-QUEST v1.9.2 (https://www.imgt.org/HighV-QUEST/login.action/) and corrected for polymerase and sequencing error rates (0.008) to calculate the frequency of somatic point mutations. To analyze huB cell clonality and SHM in the DNP-CpG-, *S*. Typhimurium flagellin-, Pfizer COVID-19 mRNA- and RBD–KLH-induced antibody responses, THX mouse huB cell V_H_DJ_H_-Cμ, V_H_DJ_H_-Cγ, V_H_DJ_H_-Cα, VκJκ-Cκ or VλJλ-Cλ transcripts were reverse transcribed, amplified and sequenced as described in ‘huBCR IgM^+^ B cell and huTCR repertoires and huIgM^+^ B and T cell clonality’, then analyzed for point mutations as described above.

B cell clonal diversity in immunized THX mice was analyzed as described in ‘huBCR IgM^+^ B cell and huTCR repertoires and huIgM^+^ B and T cell clonality’. To analyze intraclonal diversification, shared and unique point mutations in huV_H_DJ_H_-C_H_ transcripts within each huB cell clone were used to construct genealogical trees (phylogenetic maps), revealing sequential multistep accumulation of point mutations from unmutated progenitors, and allowing for detailed intraclonal diversification analysis. Genealogical trees were constructed by uploading FASTA files of all segregated huV_H_DJ_H_-C_H_ transcripts onto PHYLOViZ Online v2.0 (http://www.phyloviz.net/), which uses a JAVA implementation of the Feil’s goeBURST algorithm rules for visualization of multiple phylogenetic inference trees.

To quantify *AICDA*, *PRDM1*, huV_H_DJ_H_-Cμ, huV_H_DJ_H_-Cγ1, huV_H_DJ_H_-Cα1 and huV_H_DJ_H_-Cε transcript expression in huB cells from THX mice in vitro and ex vivo and huB cells from humans in vitro, RNA extraction and cDNA synthesis were performed as described above. Transcript expression was analyzed by SYBR Green dye (IQ SYBR Green Supermix, 115010139, Bio-Rad) incorporation in PCR reactions involving specific forward and reverse primers (Supplementary Table 16). Reactions were performed in an iCycler (Bio-Rad) real-time qPCR system under the following amplification cycles: 95 °C for 15 s, 40 cycles at 94 °C for 10 s, 60 °C for 30 s and 72 °C for 30 s—data acquisition was performed during this 72 °C extension step (Bio-Rad CFX Manager Software v3.1). Melting curve analysis was performed from 72 to 95 °C. The 2^−ΔCt^ method (2^−ΔCt^ = 2^-[Ct(*HPRT1*)-Ct(target gene)]^) was used to determine levels of transcripts, and data were normalized to levels of human *HPRT1*.

### Humanized mice antibody response to conjugated haptens

THX, huNBSGW (20–24-week-old) and JAX NSG huCD34 (23-week-old) mice were injected i.p. with 4-hydroxy-3-nitrophenylacetyl (NP) conjugated to chicken gamma globulin (NP_16_-CGG, 16 NP molecules conjugated with one CGG molecule; N-5055C-5, Biosearch Technologies) or dinitrophenyl conjugated to CpG ODN2395 (DNP-CpG, one DNP molecule conjugated with one CpG molecule, custom synthesized by Eurofins Scientific) on day 0 (100 μg in 100 μl alum, Imject Alum Adjuvant, 77161, Thermo Scientific or 50 μg in 100 μl PBS), boosted (100 μg in 100 μl PBS or 50 μg in 100 μl PBS) on day 14 and euthanized on day 28. Total, NP-specific and DNP-specific human antibodies were analyzed by specific ELISAs, as described in ‘ASCs and titration of human antibodies’. For cell sorting, NP-specific spleen huB cells from NP_16_-CGG-immunized THX mice were single-cell FACS sorted after staining with NP_16_-PE (16 NP molecules conjugated with one PE molecule, sc-396483, Santa Cruz Biotechnology; 1:100 dilution). V_H_DJ_H_-C_H_ transcripts from sorted huB cells were analyzed for SHM/CSR, B cell clonality and intraclonal diversification, as described in ‘huB cell SHM/CSR, clonality and intraclonal diversification’.

### THX mice neutralizing response to *Salmonella* and in vivo protection

THX mice (20–24 weeks old) were injected i.p. with *S*. Typhimurium flagellin (CVD1925 FliC, University of Maryland School of Medicine Center for Vaccine Development, 50 μg in 100 μl alum) or nil (100 μl alum) on day 0, boosted (50 μg in 100 μl PBS or 100 μl PBS alone) on day 14 and euthanized on day 28 (ref. ^39^).

Total human immunoglobulin and flagellin-specific human antibodies were analyzed by specific ELISAs, as described in ‘ASCs and titration of human antibodies’. Bactericidal activity of flagellin-induced antibodies in sera from flagellin-vaccinated and non-vaccinated THX mice was measured by in vitro killing of *S*. Typhimurium^39^. *S*. Typhimurium IR715, a virulent nalidixic acid-resistant derivative of wild-type isolate ATCC 14028 (provided by M. Raffatellu, University of California, San Diego) was grown in LB broth (BP1426-2, Fisher Scientific) overnight at 37 °C. Log-phase cultures were prepared by diluting overnight cultures to an OD_600_ of 0.05 in fresh LB medium and incubating them at 37 °C, with shaking at 250 rpm until an OD_600_ of 0.7 or 0.8 was attained. Stock cultures were prepared by diluting 500 µl of log-phase cultures in 500 µl of 50% sterile filtered glycerol (G33-1, Fisher Scientific) then further diluted in PBS to a cell density of approximately 10^4^ CFUs per ml. Sera from flagellin-vaccinated THX mice, non-vaccinated THX mice and healthy humans were serially twofold diluted in PBS in round-bottom 96-well plates. Diluted sera (50 µl) or PBS (50 µl, negative control) were mixed with 25 µl baby-rabbit complement (CL3441, CEDARLANE, 25% final concentration) and incubated with 25 µl diluted *S*. Typhimurium (250 CFUs). Each sample mixture was shaken (115 rpm) at 37 °C for 1 h and then struck onto LB-agar plates. These were incubated at 37 °C overnight, after which CFUs were enumerated. To assess the protective response induced by flagellin vaccination in vivo, flagellin-vaccinated and non-vaccinated THX mice were infected orally with *S*. Typhimurium (1 × 10^5^ CFUs) by gavage on day 21. The effective dose of bacteria given to mice was verified by plating dilutions of *S*. Typhimurium on LB-agar plates supplemented with nalidixic acid (N8878-25G, Sigma-Aldrich, 0.05 mg ml^−1^). Mice were monitored for 10 days, and Kaplan–Meier survival plots were generated (GraphPad Prism v10.0.3). For cell sorting, flagellin-specific spleen huB cells from flagellin-vaccinated THX mice underwent single-cell FACS after staining with AF647-flagellin (synthesized using iLink Andy Fluor 647 Antibody Labeling Kit, L038, ABP Biosciences). V_H_DJ_H_-C_H_ transcripts from sorted huB cells were analyzed for SHM/CSR, B cell clonality and intraclonal diversification, as described in ‘huB cell SHM/CSR, clonality and intraclonal diversification’.

### THX mice neutralizing antibody response to COVID-19 mRNA or RBD–KLH

THX mice (20–24 weeks old) were injected i.m. with Pfizer-BioNTech 162b2 COVID-19 vaccine (Pfizer COVID-19 mRNA, 5 µg in 50 µl PBS) or nil (50 µl PBS) on day 0, boosted (5 µg in 50 µl PBS or 50 µl PBS alone) on day 21, according to the human vaccination schedule, and euthanized on day 28. ‘Discarded’ vials of Pfizer COVID-19 mRNA vaccine were obtained from The University of Texas Health Science Center at San Antonio vaccination facility within 6 h of opening and contained less than one full vaccine dose, thereby not diverting any amount of vaccine from humans for the purpose of this study. THX mice were injected i.p. with SARS-CoV-2 Spike S1 RBD (47 amino acid peptide containing the core 37 amino acids: FRKSNLKPFERDISTEIYQAGSTPCNGVEGFNCYFPLQSYGFQPTNG, custom synthesized by ABI scientific) conjugated to KLH (RBD–KLH, 50 μg in 100 μl alum) or nil (100 μl alum) on day 0, boosted (50 μg in 100 μl PBS or 100 μl PBS alone) on day 21 and euthanized on day 28.

Total human immunoglobulin and RBD-specific human antibodies or ASCs were analyzed by specific ELISAs or ELISPOTs, as described in ‘ASCs and titration of human antibodies’. The SARS-CoV-2 neutralization power of antibodies induced by COVID-19 mRNA vaccine in THX mice was measured using two different platforms: SARS-CoV-2 Neutralizing Antibody Detection ELISA Kit (502070, Cayman Chemical) and SeroFlash SARS-CoV-2 Neutralizing Antibody Assay Fast Kit (D-1008-96, EpigenTek), according to the manufacturer’s instructions. Sera from COVID-19 mRNA-vaccinated THX mice were serially twofold diluted in PBS-Tween 20 in 96-well plates pre-coated with SARS-CoV-2 Spike S1 RBD peptide (EpigenTek platform), or a recombinant rabbit Fc-tagged SARS-CoV-2 Spike S1 RBD peptide bound to an anti-rabbit Fc-specific antibody (Cayman platform), followed by addition of recombinant His-tagged ACE2 protein to each well. These platforms use a horseradish peroxidase (HRP)-conjugated anti-His antibody in an inhibition/competition assay to measure serum neutralizing human antibody concentration by OD reading at 450 nm. High OD readings reflect a low concentration of neutralizing antibodies, while low OD readings reflect a high concentration. SARS-CoV-2-neutralizing human monoclonal antibodies were provided as a positive control by EpigenTek and Cayman. Extensive controls performed by both Cayman Chemical and EpigenTek have validated measurements of their RBD competition assays with actual virus neutralization in COVID-19-positive and COVID-19-negative human sera (https://www.caymanchem.com/product/502070/sars-cov-2-neutralizing-antibody-detection-elisa-kit; www.epigentek.com/docs/D-1008.pdf).

Sequencing and cloning of original paired heavy-chain V_H_DJ_H_-C_H_ and light-chain VκJκ-Cκ or VλJλ-Cλ gene segments for construction of human antibody-producing cell microcultures was performed by The University of Texas MD Anderson Cancer Center Recombinant Antibody Production Core. Briefly, RBD-specific spleen huB cells of three COVID-19 mRNA-vaccinated THX mice underwent single-cell FACS using biotinylated RBD peptide (47 amino acids) and FITC-streptavidin (405201, BioLegend). huV_H_DJ_H_-C_H_ and light-chain huVκJκ-Cκ or huVλJλ-Cλ gene segments from sorted huB cells were amplified as cDNAs by single-cell RT–PCR and then sequenced. The single B cell huIgH constant region and huIGκ or huIGλ constant regions were determined. The amplified huV_H_DJ_H_ and huVκJκ or huVλJλ cDNAs were sequenced and cloned into pcDNA3.4 vectors that included the coding sequence for either human heavy-chain (γ1) or light-chain (κ or λ) constant regions to transfect ExpiCHO cells (A29127, Thermo Fisher). Transfected ExpiCHO cells were cultured in ExpiCHO Expression Medium (A2910001, Thermo Fisher) in 100 single-cell microcultures to produce recombinant human monoclonal antibodies. After 5 days, media were collected and analyzed for RBD-specific recombinant human antibodies by specific ELISA.

### ASCs and titration of human antibodies

To measure total or specific huIgM, huIgD, huIgG (huIgG1, huIgG2, huIgG3 and huIgG4), huIgA or huIgE in humanized mice, sera were diluted 400-fold or 20-fold in PBS containing 0.05% vol/vol Tween 20 (BP337-500, Fisher Scientific; PBS-Tween 20), followed by serial twofold dilution. Serially diluted samples were incubated at room temperature in 96-well plates pre-coated with goat anti-huIgM antibody (2020-01, SouthernBiotech, 1.0 µg ml^−1^), goat anti-huIgD antibody (2030-01, SouthernBiotech, 1.0 µg ml^−1^), goat anti-huIgG antibody (huIgG1, huIgG2, huIgG3 and huIgG4, 2015-01, SouthernBiotech, 1.0 µg ml^−1^), goat anti-huIgA antibody (2050-01, SouthernBiotech, 1.0 µg ml^−1^), goat anti-huIgE antibody (GE-80A, ICL Labs, 1.0 µg ml^−1^), NP_4_-BSA (four NP molecules per one BSA molecule, Biosearch Technologies, 1.0 µg ml^−1^), DNP_5.6_-BSA (average of 5.6 DNP molecules per one BSA molecule, Cosmo Bio USA, 1.0 µg ml^−1^, referred to as DNP_5_ in the Results and figure legends), BSA (Biosearch Technologies, 1.0 µg ml^−1^), *S*. Typhimurium flagellin (2.0 µg ml^−^^1^) or SARS-CoV-2 Spike S1 RBD peptide (37 amino acid core peptide, FRKSNLKPFERDISTEIYQAGSTPCNGVEGFNCYFPL, ABI Scientific, 2.0 µg ml^−1^) in 0.1 M sodium carbonate/bicarbonate buffer at pH 9.6. After washing plates with PBS-Tween 20, bound human antibodies were detected with biotinylated goat anti-huIgM antibody (2020-08, SouthernBiotech; 1:5,000 dilution), goat anti-huIgD antibody (2030-08, SouthernBiotech; 1:5,000 dilution), goat anti-huIgG antibody (2015-08, SouthernBiotech; 1:5,000 dilution), goat anti-huIgG1 monoclonal antibody (555869, BD Pharmingen; 1:5,000), goat anti-huIgG2 monoclonal antibody (555874, BD Pharmingen; 1:5,000 dilution), goat anti-huIgG3 monoclonal antibody (3853-6-250, MABTECH; 1:5,000 dilution), goat anti-huIgG4 monoclonal antibody (555882, BD Pharmingen; 1:5,000 dilution), goat anti-huIgA antibody (2050-08, SouthernBiotech; 1:5,000 dilution) or goat anti-huIgE antibody (9250-08, SouthernBiotech; 1:5,000 dilution; Supplementary Table 15), followed by reaction with HRP-labeled streptavidin (405210, BioLegend), development with *O*-phenylenediamine substrate (P8806-50TAB, Sigma-Aldrich) or 3, 3′, 5, 5′ tetramethyl benzidine substrate (421101, BioLegend), and measurement of converted substrate absorbance at 492 nm or 450 nm, respectively. Total human antibody concentrations or specific human antibody titers were calculated from OD readings (using a reference curve constructed with known antibody concentrations; BioTek Gen5 Software v2.07) and expressed as µg equivalent per ml (µg eq ml^−1^) or RUs (defined as the dilution factor needed to reach 50% saturation binding) using GraphPad Prism v10.0.3 software or Excel v16.83 (Microsoft) software. To measure BALF human immunoglobulin concentrations, DNP-CpG-immunized THX and huNBSGW mice were euthanized on day 28, and lungs were lavaged with 1 ml PBS containing 0.1 mM EDTA. Human immunoglobulin concentrations were measured from the recovered 1 ml lavage fluids by specific ELISA as described above.

To detect huASCs (huPBs/PCs) by ELISPOT, splenic or BM cells from DNP-CpG-immunized or COVID-19 mRNA-vaccinated THX mice were suspended in FBS-RPMI then cultured at 37 °C overnight in 96-well PVDF Multi-Screen filter plates (activated with 35% ethanol, MAIPS4510, Millipore) coated with goat anti-huIgM antibody, goat anti-huIgG antibody, goat anti-huIgA antibody, DNP_5.6_-BSA or SARS-CoV-2 RBD peptide (all 5 µg ml^−1^). Spleen and BM cells were plated at 1.25 × 10^5^ and 2.5 × 10^5^ cells per well to analyze total and specific huASCs, respectively. After removing supernatants, plates were incubated with biotinylated goat anti-huIgM antibody, goat anti-huIgG or goat anti-huIgA antibody for 2 h, and then, after washing, incubated with HRP-conjugated streptavidin, followed by Vectastain AEC peroxidase substrate (SK-4200, Vector Laboratories). Individual ASC spots were detected using a CTL Immunospot Analyzer and software (CTL ImmunoCapture Software v6.5.7, Cellular Technology).

### Human cytokines

To measure circulating human cytokines, sera were collected from flagellin-vaccinated and COVID-19 mRNA-vaccinated THX mice and analyzed for huAPRIL, huBAFF, huIFN-γ, huIL-2, huIL-4, huIL-6, huIL-10 and huIL-21 by Luminex Human Discovery Assay 8-Plex (LXSAHM-08, R&D Systems). Analysis of huTGF-β1 was performed by TGF-β Premixed Magnetic Luminex Performance Assay (FCSTM17, R&D Systems). Samples and reagents were prepared according to the manufacturer’s instructions. Briefly, sera were diluted at a 1:2.5 (Luminex Human Discovery Assay) or 1:15 (TGF-β Luminex Performance Assay) ratio in Calibrator Diluent RD6-52 or Calibrator Diluent RD6-50, respectively. Next, 50 μl working standards and 50 μl diluted sera were each mixed with 50 μl Human Magnetic Premixed Microparticle Cocktail (color-coded magnetic beads coated with analyte-specific capture antibodies) and incubated in 96-well microplates at room temperature for 2 h with shaking at 800 rpm. After washing plates with 100 μl per well of wash buffer using a Luminex microplate magnet, human cytokines were detected by addition of 50 μl Human Premixed Biotin-Antibody cocktail (biotinylated detection monoclonal antibodies specific for analytes of interest) followed by reaction with 50 μl streptavidin–phycoerythrin and measurement using a dual-laser flow-based detection Luminex FLEXMAP 3D analyzer (Luminex). One laser classifies the beads and determines the analyte that is being detected. The second laser determines the magnitude of the PE-derived signal, which is proportional to the amount of analyte bound. Cytokine concentrations were calculated using Belysa Immunoassay Curve Fitting Software (40–122, MilliporeSigma) and compared to human physiological range^93–98^.

### H&E, immunohistochemistry and immunofluorescence microscopy

#### H&E and immunohistochemistry

To identify GCs in humanized mice, NP_16_-CGG-immunized THX, huNBSGW and JAX NSG huCD34 mouse spleens were fixed in paraformaldehyde (4%) overnight. Spleens were embedded in paraffin, sectioned, then stained with H&E or anti-huCD20 monoclonal antibody (1:200 dilution), anti-huCD3 monoclonal antibody (1:200 dilution), anti-huKi67 monoclonal antibody (1:200 dilution), anti-huBCL6 monoclonal antibody (1:200 dilution), anti-huAID (1:200 dilution) or anti-huBLIMP-1 monoclonal antibody (1:200 dilution), followed by reaction with anti-mouse IgG-HRP and brown precipitating HRP substrate 3,3′-diaminobenzidine (DAB). Spleen sectioning and staining was performed at The University of Texas Health Science Center at San Antonio Histology and Immunohistochemistry Laboratory. Images were captured using a Zeiss Imager-V.1 (ZEN Microscopy Software v3.9, 1× and 20× objective).

#### Immunofluorescence microscopy

To detect gut huB cells, huT cells, and huIgM-, huIgD- and huIgA-producing cells, DNP-CpG-immunized THX mouse intestines were fixed in paraformaldehyde (4%) overnight. Intestines were sectioned, then heated at 80 °C to adhere to glass slides, washed four times in xylene (214736-1L, Millipore Sigma) for 2 min, dehydrated twice with 100% ethanol for 1 min, dehydrated twice with 95% ethanol for 1 min, and washed twice in water for 1 min. Antigens were unmasked using 2 mM EDTA (15-575-020, Fisher Scientific) in 100 °C for 40 min, followed by a cooling step at 25 °C, thrice washing with TBS (15-567-027, Fisher Scientific) and final blocking by 10% BSA (BP1600-100, Fisher Scientific) for 15 min. Slides were washed again thrice with TBS and then stained with PE-Cyanine7-anti-huCD19 monoclonal antibody (clone HIB19, 302216, TONBO; 1:100 dilution), Super Bright 600-anti-huCD3 monoclonal antibody (clone OKT3, 63003741, eBioscience; 1:100 dilution), BV510-anti-huIgM monoclonal antibody (clone MHM-88, 314521, BioLegend; 1:100 dilution), BV421-anti-huIgD monoclonal antibody (clone HB-7, 348225, BioLegend; 1:100 dilution) or APC-anti-huIgA monoclonal antibody (clone IS11-8E10, 130-113-427, Miltenyi Biotec; 1:100 dilution) for 2 h in a dark, moist chamber (Supplementary Table 13). After washing thrice with 0.1% Triton X-100 (T9284, Sigma-Aldrich) in TBS, slides were air-dried, and coverslips were mounted using ProLong Gold Antifade Reagent with DAPI (P36935, Thermo Fisher Scientific). To detect human and mouse TECs, THX and huNBSGW mice thymi were snap frozen in Tissue-Tek O.C.T. Compound (45583, Sakura), sectioned by cryostat, loaded onto positively charged slides, fixed in cold acetone and stained with PE-anti-huEpCAM (ab237397, Abcam; 1:100 dilution) and PE-Cyanine7-anti-moEpCAM (118216, BioLegend; 1:100 dilution) monoclonal antibodies for 2 h at 25 °C in a moist chamber. Cover slips were then mounted on slides using ProLong Gold Antifade Reagent with DAPI. Fluorescent images were captured using a Zeiss Imager-V.1 (ZEN Microscopy Software v3.9, 20x objective).

### Intestinal microbiota

Microbial DNA was extracted from feces of non-intentionally immunized THX, huNBSGW and NBSGW mice (22 weeks old) using Quick-DNA Fecal/Soil Microbe Microprep Kit (Zymo Research) according to the manufacturer’s instructions. To analyze gut bacterial microbiome composition, microbial DNA was tagged and sequenced using the Illumina MiSeq platform. Briefly, the V3–V4 hypervariable region of the bacteria 16S rRNA gene was amplified by PCR using tagged bact-341F primer 5′-TCGTCGGCAGCGTCAGATGTGTATAAGAGACAGCCTACGGGNGGCWGCAG-3′, bact-850R primer 5′-GTCTCGTGGGCTCGGAGATGTGTATAAGAGACAGGACTACHVGGGTATCTAATCC-3′ and Phusion high-fidelity DNA polymerase (M0530S, New England BioLabs). Multiplexing indices and Illumina sequencing adaptors were then added to the amplicons by limited-cycle amplification using the Nextera XT Index Kit (Illumina). Libraries were normalized, pooled and sequenced using the Illumina MiSeq platform. Sequencing and quality assessment were performed by The University of Texas Health Science Center at San Antonio Genome Sequencing Facility. Bacterial taxonomy was assigned using the Ribosomal Database Project (RDP) classifier v2.14 (http://rdp.cme.msu.edu/classifier/). Principle component analysis of gut bacterial composition in THX, huNBSGW and NBSGW mice was performed by ClustVis v1.0 (biit.cs.ut.ee/clustvis/), which uses clustering algorithms to construct plots visualizing similarities and/or differences between groups of samples.

### Lupus THX mice, human autoantibodies, immunopathology and mortality

Lupus THX mice were generated by i.p. injection of 11 male and female THX mice (18 weeks old), constructed by huCD34^+^ cell engraftment of 6 NBSGW (2 males and 4 females) mice and 5 NSGW41 (2 males and 3 females) mice once with pristane (2,6,10,14-tetramethylpentadecane, P2870, Millipore Sigma, 500 μl) and continuing E2 treatment (Supplementary Table 17). Healthy THX controls (18-week-old) were constructed by huCD34^+^ cell engraftment of 8 NBSGW and 4 NSGW41 mice. Three additional healthy THX controls (18 weeks old) constructed by huCD34^+^ cell engraftment of NBSGW mice were used for ex vivo immune cell analyses and immunopathology control experiments and staining.

To measure total human immunoglobulin levels or specific human antibodies, sera from Lupus THX and control THX mice (injected with 500 µl PBS) were collected 6 weeks after pristane or PBS injection, serially twofold diluted then incubated at room temperature in 96-well plates coated with pre-adsorbed goat anti-huIgM antibody (1 µg ml^−1^), goat anti-huIgG antibody (1 µg ml^−1^), goat anti-huIgA antibody (1 µg ml^−1^), dsDNA (15632011, Thermo Fisher Scientific, 10 µg ml^−1^), histone (16736, Cayman Chemicals, 1.0 µg ml^−1^), Sm/RNP (A11600, Surmodics, 1.0 µg ml^−1^) or mouse liver RNA (10 µg ml^−1^). Total human antibody concentrations or specific human autoantibody titers were measured by specific ELISAs, as described in ‘ASCs and titration of human antibodies’.

To detect human antinuclear antibodies, sera from Lupus THX and healthy control THX mice, collected at 6 weeks after pristane injection, were serially diluted (from 1:50 to 1:400) in PBS and incubated on Hep-2 cell-coated slides (ANK-120, MBL-BION). Bound huIgGs were detected with FITC-anti-huIgG monoclonal antibody (clone G18-145, 555786, BD Pharmingen). Analysis of SHM/CSR, huB/huT cell clonality and intraclonal diversification in Lupus THX mice (6 weeks after pristane injection) was performed, as described in ‘huBCR IgM^+^ B cell and huTCR repertoires and huIgM^+^ B and T cell clonality’ and ‘huB cell SHM/CSR, clonality and intraclonal diversification’, in Lupus THX mice euthanized when showing obvious signs of disease and the three ‘additional’ healthy controls at corresponding ages (THX mice). To detect kidney huIgG deposition, Lupus THX and THX mice kidneys were processed for H&E and immunofluorescence staining then imaged as described in ‘H&E, immunohistochemistry and immunofluorescence microscopy’. Mortality of Lupus THX mice and THX mice was analyzed and depicted by Kaplan–Meier survival plots (GraphPad Prism v10.0.3).

### Mouse IACUC and human Institutional Review Board protocols

Buffy coats were obtained upon informed consent from healthy donors, per the protocol of the South Texas Blood and Tissue Center. Human umbilical cord blood was collected from full-term, normally developed male and female newborns from healthy puerperae at the Department of Obstetrics and Gynecology, The University of Texas Long School of Medicine, The University of Texas Health Science Center at San Antonio, and obtained upon informed consent, per Institutional Review Board Protocol 17-653H. All experiments involving mice were performed in compliance with the animal protocol approved by The University of Texas Health Science Center at San Antonio Institutional Animal Care and Use Committee (IACUC protocol 20200019AR).

### Sample size, randomization and statistical analysis

The exact sample size of all experiments is reported in the figure legends. In each experiment, at least five mice per group (except for the experiment of Fig. 1g) were used to ensure proper biological replicates. Sample size calculations were performed using power analysis, which accounts for effect size, standard deviation, type 1 error and 80% power in a two-sample *t*-test with a 5% significance level (two-sided test). G power software version 3.1.9.7 was used for these calculations. To construct humanized mice, immunodeficient mice from one litter were grafted with huCD34^+^ cells from the same donor. In those cases, in which litter sizes were small, multiple litters were combined and grafted with the same donor huCD34^+^ cells, and pups cross-fostered by a single nursing mother.

Replication: biological replicates were used in all experiments.

Randomization: After matching for sex and age, THX, huNBSGW, huNSG and JAX NSG huCD34 mice were randomly assigned to appropriate groups.

Statistical analyses: statistical analyses were performed using Excel v16.83 (Microsoft) or GraphPad Prism v10.0.3. Differences in antibody concentrations, cell proportions or numbers and RNA transcript expression were analyzed by two-sided Student’s unpaired *t*-test. Differences in mouse survival were analyzed by log-rank (Mantel–Cox) test.

Experimenters were blinded to group allocation for both data collection and analysis whenever possible. Experimenters were not blinded to group allocation during experimental sample collection.

Generally, THX and huNBSGW mice used in all experiments displayed up to 96.1% and 89.3% huCD45^+^ cells, respectively, in circulating blood. Generally, 2–3% of the constructed THX and huNBSGW mice at age 20–24 weeks displayed less than 90% and 88% huCD45^+^ cells in circulating blood, respectively, and were excluded from the study. Around 60% of huNSG and JAX NSG huCD34 mice displayed at peak approximately 45% and 20% huCD45^+^ cells, respectively, in circulating blood. huNSG and JAX NSG huCD34 mice displaying lower proportions of peak circulating blood human CD45^+^ cells were excluded from study. No data were excluded from analysis in vivo and in vitro.

### Reporting summary

Further information on research design is available in the Nature Portfolio Reporting Summary linked to this article.

## Online content

Any methods, additional references, Nature Portfolio reporting summaries, source data, extended data, supplementary information, acknowledgements, peer review information; details of author contributions and competing interests; and statements of data and code availability are available at 10.1038/s41590-024-01880-3.

### Supplementary information

Supplementary Information Supplementary Figs. 1 and 2 and Supplementary Tables 1–17.

Reporting Summary

### Source data

Source Data Fig. 1 Statistical source data.

Source Data Fig. 4 Statistical source data.

Source Data Fig. 5 Statistical source data.

Source Data Fig. 6 Statistical source data.

Source Data Fig. 7 Statistical source data.

Source Data Fig. 8 Statistical source data.

Source Data Extended Data Fig. 1 Statistical source data.

Source Data Extended Data Fig. 2 Statistical source data.

Source Data Extended Data Fig. 3 Statistical source data.

Source Data Extended Data Fig. 6 Statistical source data.

Source Data Extended Data Fig. 8 Statistical source data.

Source Data Extended Data Fig. 9 Statistical source data.

Source Data Extended Data Fig. 10 Statistical source data.

## Data Availability

MiSeq amplicon sequencing data have been deposited in NCBI’s Sequence Read Archive under the BioProject code PRJNA1047643. Source data are provided with this paper. All other data supporting the findings of this study are present in the article and Supplementary Information.

## References

[1] Zschaler, J., Schlorke, D. & Arnhold, J. Differences in innate immune response between man and mouse. *Crit. Rev. Immunol.* **34**, 433–454 (2014). 25404048

[2] Allen, T. M. et al. Humanized immune system mouse models: progress, challenges and opportunities. *Nat. Immunol.* **20**, 770–774 (2019). 31160798 10.1038/s41590-019-0416-z PMC7265413

[3] Shultz, L. D. et al. Humanized mouse models of immunological diseases and precision medicine. *Mamm. Genome* **30**, 123–142 (2019). 30847553 10.1007/s00335-019-09796-2 PMC6610695

[4] Stripecke, R. et al. Innovations, challenges, and minimal information for standardization of humanized mice. *EMBO Mol. Med.* **12**, e8662 (2020). 32578942 10.15252/emmm.201708662 PMC7338801

[5] Martinov, T. et al. Building the next generation of humanized hemato-lymphoid system mice. *Front. Immunol.* **12**, 643852 (2021). 33692812 10.3389/fimmu.2021.643852 PMC7938325

[6] Ye, W. & Chen, Q. Potential applications and perspectives of humanized mouse models. *Annu. Rev. Anim. Biosci.* **10**, 395–417 (2022). 34758273 10.1146/annurev-animal-020420-033029

[7] Ito, M. et al. NOD/SCID/γ_c_^null^ mouse: an excellent recipient mouse model for engraftment of human cells. *Blood* **100**, 3175–3182 (2002). 12384415 10.1182/blood-2001-12-0207

[8] Shultz, L. D. et al. Human lymphoid and myeloid cell development in NOD/LtSz-*scid IL2R*γ^null^ mice engrafted with mobilized human hemopoietic stem cells. *J. Immunol.* **174**, 6477–6489 (2005). 15879151 10.4049/jimmunol.174.10.6477

[9] Takenaka, K. et al. Polymorphism in Sirpa modulates engraftment of human hematopoietic stem cells. *Nat. Immunol.* **8**, 1313–1323 (2007). 17982459 10.1038/ni1527

[10] Yu, H. et al. A novel humanized mouse model with significant improvement of class-switched, antigen-specific antibody production. *Blood* **129**, 959–969 (2017). 28077418 10.1182/blood-2016-04-709584 PMC5324713

[11] Cosgun, K. N. et al. Kit regulates HSC engraftment across the human-mouse species barrier. *Cell Stem Cell* **15**, 227–238 (2014). 25017720 10.1016/j.stem.2014.06.001

[12] McIntosh, B. E. et al. Nonirradiated NOD,B6.SCID Il2rγ^-/-^ KitW41/W41 (NBSGW) mice support multilineage engraftment of human hematopoietic cells. *Stem Cell Rep.* **4**, 171–180 (2015). 10.1016/j.stemcr.2014.12.005 PMC4325197 25601207

[13] Nakada, D. et al. Oestrogen increases haematopoietic stem-cell self-renewal in females and during pregnancy. *Nature* **505**, 555–558 (2014). 24451543 10.1038/nature12932 PMC4015622

[14] Kovats, S. Estrogen receptors regulate innate immune cells and signaling pathways. *Cell Immunol.* **294**, 63–69 (2015). 25682174 10.1016/j.cellimm.2015.01.018 PMC4380804

[15] Kumar, R. S. & Goyal, N. Estrogens as regulator of hematopoietic stem cell, immune cells and bone biology. *Life Sci.* **269**, 119091 (2021). 33476629 10.1016/j.lfs.2021.119091

[16] Grimaldi, C. M., Cleary, J., Dagtas, A. S., Moussai, D. & Diamond, B. Estrogen alters thresholds for B cell apoptosis and activation. *J. Clin. Invest.* **109**, 1625–1633 (2002). 12070310 10.1172/JCI14873 PMC151010

[17] Venkatesh, J., Peeva, E., Xu, X. & Diamond, B. Cutting edge: hormonal milieu, not antigenic specificity, determines the mature phenotype of autoreactive B cells. *J. Immunol.* **176**, 3311–3314 (2006). 16517697 10.4049/jimmunol.176.6.3311

[18] Cohen-Solal, J. F. et al. Hormonal regulation of B-cell function and systemic lupus erythematosus. *Lupus* **17**, 528–532 (2008). 18539705 10.1177/0961203308089402

[19] Hill, L., Jeganathan, V., Chinnasamy, P., Grimaldi, C. & Diamond, B. Differential roles of estrogen receptors α and β in control of B-cell maturation and selection. *Mol. Med* **17**, 211–220 (2011). 21107497 10.2119/molmed.2010.00172 PMC3060981

[20] Khan, D. & Ansar Ahmed, S. The immune system is a natural target for estrogen action: opposing effects of estrogen in two prototypical autoimmune diseases. *Front. Immunol.* **6**, 635 (2016). 26779182 10.3389/fimmu.2015.00635 PMC4701921

[21] Klein, S. L. & Flanagan, K. L. Sex differences in immune responses. *Nat. Rev. Immunol.* **16**, 626–638 (2016). 27546235 10.1038/nri.2016.90

[22] Moulton, V. R. Sex hormones in acquired immunity and autoimmune disease. *Front. Immunol.* **9**, 2279 (2018). 30337927 10.3389/fimmu.2018.02279 PMC6180207

[23] Graham, J. H., Yoachim, S. D. & Gould, K. A. Estrogen receptor alpha signaling is responsible for the female sex bias in the loss of tolerance and immune cell activation induced by the lupus susceptibility locus *Sle1b*. *Front Immunol.* **11**, 582214 (2020). 33240270 10.3389/fimmu.2020.582214 PMC7683613

[24] Fananas-Baquero, S. et al. Natural estrogens enhance the engraftment of human hematopoietic stem and progenitor cells in immunodeficient mice. *Haematologica* **106**, 1659–1670 (2021). 32354868 10.3324/haematol.2019.233924 PMC8168497

[25] Chakraborty, B. et al. Estrogen receptor signaling in the immune system. *Endocr. Rev.* **44**, 117–141 (2023). 35709009 10.1210/endrev/bnac017

[26] Hoffmann, J. P., Liu, J. A., Seddu, K. & Klein, S. L. Sex hormone signaling and regulation of immune function. *Immunity* **56**, 2472–2491 (2023). 37967530 10.1016/j.immuni.2023.10.008

[27] Park, S. R. et al. HoxC4 binds to the promoter of the cytidine deaminase AID gene to induce AID expression, class-switch DNA recombination and somatic hypermutation. *Nat. Immunol.* **10**, 540–550 (2009). 19363484 10.1038/ni.1725 PMC2753990

[28] Pauklin, S., Sernandez, I. V., Bachmann, G., Ramiro, A. R. & Petersen-Mahrt, S. K. Estrogen directly activates AID transcription and function. *J. Exp. Med.* **206**, 99–111 (2009). 19139166 10.1084/jem.20080521 PMC2626679

[29] Mai, T. et al. Estrogen receptors bind to and activate the *HOXC4/HoxC4* promoter to potentiate *HoxC4*-mediated activation-induced cytosine deaminase induction, immunoglobulin class switch DNA recombination, and somatic hypermutation. *J. Biol. Chem.* **285**, 37797–37810 (2010). 20855884 10.1074/jbc.M110.169086 PMC2988384

[30] Casali, P. et al. Estrogen reverses HDAC inhibitor-mediated repression of *Aicda* and class-switching in antibody and autoantibody responses by downregulation of miR-26a. *Front. Immunol.* **11**, 491 (2020). 32265934 10.3389/fimmu.2020.00491 PMC7105609

[31] Gualdron-Lopez, M. et al. Multiparameter flow cytometry analysis of the human spleen applied to studies of plasma-derived EVs from *Plasmodium vivax* patients. *Front. Cell Infect. Microbiol.* **11**, 596104 (2021). 33732657 10.3389/fcimb.2021.596104 PMC7957050

[32] Lefranc, M. P. Immunoglobulin and T cell receptor genes: IMGTI and the birth and rise of immunoinformatics. *Front. Immunol.* **5**, 22 (2014). 24600447 10.3389/fimmu.2014.00022 PMC3913909

[33] Kubinak, J. L. & Round, J. L. Do antibodies select a healthy microbiota? *Nat. Rev. Immunol.* **16**, 767–774 (2016). 27818504 10.1038/nri.2016.114 PMC9004535

[34] King, C. H. et al. Baseline human gut microbiota profile in healthy people and standard reporting template. *PLoS ONE* **14**, e0206484 (2019). 31509535 10.1371/journal.pone.0206484 PMC6738582

[35] Moroney, J. B., Vasudev, A., Pertsemlidis, A., Zan, H. & Casali, P. Integrative transcriptome and chromatin landscape analysis reveals distinct epigenetic regulations in human memory B cells. *Nat. Commun.* **11**, 5435 (2020). 33116135 10.1038/s41467-020-19242-6 PMC7595102

[36] Chang, B. & Casali, P. The CDR1 sequences of a major proportion of human germline Ig V_H_ genes are inherently susceptible to amino acid replacement. *Immunol. Today* **15**, 367–373 (1994). 7916950 10.1016/0167-5699(94)90175-9 PMC4665105

[37] Pone, E. J. et al. BCR-signalling synergizes with TLR-signalling for induction of AID and immunoglobulin class-switching through the non-canonical NF-κB pathway. *Nat. Commun.* **3**, 767 (2012). 22473011 10.1038/ncomms1769 PMC3337981

[38] Sanchez, H. N. et al. B cell-intrinsic epigenetic modulation of antibody responses by dietary fiber-derived short-chain fatty acids. *Nat. Commun.* **11**, 60 (2020). 31896754 10.1038/s41467-019-13603-6 PMC6940392

[39] Rivera, C. E. et al. Intrinsic B cell TLR-BCR linked coengagement induces class-switched, hypermutated, neutralizing antibody responses in absence of T cells. *Sci. Adv.* **9**, eade8928 (2023). 37115935 10.1126/sciadv.ade8928 PMC10146914

[40] Takaba, H. & Takayanagi, H. The mechanisms of T cell selection in the thymus. *Trends Immunol.* **38**, 805–816 (2017). 28830733 10.1016/j.it.2017.07.010

[41] Wang, H. X. et al. Thymic epithelial cells contribute to thymopoiesis and T cell development. *Front. Immunol.* **10**, 3099 (2019). 32082299 10.3389/fimmu.2019.03099 PMC7005006

[42] Castaneda, J. et al. The multifaceted roles of B cells in the thymus: from immune tolerance to autoimmunity. *Front. Immunol.* **12**, 766698 (2021). 34790201 10.3389/fimmu.2021.766698 PMC8591215

[43] Richard, M. L. & Gilkeson, G. Mouse models of lupus: what they tell us and what they don’t. *Lupus Sci. Med* **5**, e000199 (2018). 29387435 10.1136/lupus-2016-000199 PMC5786947

[44] Li, Y. et al. A human immune system mouse model with robust lymph node development. *Nat. Methods* **15**, 623–630 (2018). 30065364 10.1038/s41592-018-0071-6

[45] Flanagan, K. L., Fink, A. L., Plebanski, M. & Klein, S. L. Sex and gender differences in the outcomes of vaccination over the life course. *Annu. Rev. Cell Dev. Biol.* **33**, 577–599 (2017). 28992436 10.1146/annurev-cellbio-100616-060718

[46] Fischinger, S., Boudreau, C. M., Butler, A. L., Streeck, H. & Alter, G. Sex differences in vaccine-induced humoral immunity. *Semin. Immunopathol.* **41**, 239–249 (2019). 30547182 10.1007/s00281-018-0726-5 PMC6373179

[47] Wilkinson, N. M., Chen, H. C., Lechner, M. G. & Su, M. A. Sex differences in immunity. *Annu. Rev. Immunol.* **40**, 75–94 (2022). 34985929 10.1146/annurev-immunol-101320-125133 PMC9805670

[48] Notta, F., Doulatov, S. & Dick, J. E. Engraftment of human hematopoietic stem cells is more efficient in female NOD/SCID/IL-2Rg_c_-null recipients. *Blood* **115**, 3704–3707 (2010). 20207983 10.1182/blood-2009-10-249326

[49] Monteiro, C. et al. Human pregnancy levels of estrogen and progesterone contribute to humoral immunity by activating T_FH_/B cell axis. *Eur. J. Immunol.* **51**, 167–179 (2021). 33012073 10.1002/eji.202048658

[50] Weill, J. C., Weller, S. & Reynaud, C. A. Human marginal zone B cells. *Annu. Rev. Immunol.* **27**, 267–285 (2009). 19302041 10.1146/annurev.immunol.021908.132607

[51] Jones, B. G. et al. Binding of estrogen receptors to switch sites and regulatory elements in the immunoglobulin heavy chain locus of activated B cells suggests a direct influence of estrogen on antibody expression. *Mol. Immunol.* **77**, 97–102 (2016). 27494228 10.1016/j.molimm.2016.07.015 PMC5010968

[52] Aguilar-Pimentel, J. A. et al. Increased estrogen to androgen ratio enhances immunoglobulin levels and impairs B cell function in male mice. *Sci. Rep.* **10**, 18334 (2020). 33110090 10.1038/s41598-020-75059-9 PMC7591566

[53] Pauklin, S. & Petersen-Mahrt, S. K. Progesterone inhibits activation-induced deaminase by binding to the promoter. *J. Immunol.* **183**, 1238–1244 (2009). 19553525 10.4049/jimmunol.0803915

[54] Hall, O. J. & Klein, S. L. Progesterone-based compounds affect immune responses and susceptibility to infections at diverse mucosal sites. *Mucosal Immunol.* **10**, 1097–1107 (2017). 28401937 10.1038/mi.2017.35

[55] Hall, O. J. et al. Progesterone-based contraceptives reduce adaptive immune responses and protection against sequential influenza A virus infections. *J. Virol.* **91**, e02160-16 (2017). 28179523 10.1128/JVI.02160-16 PMC5375688

[56] Jalkanen, P. et al. COVID-19 mRNA vaccine induced antibody responses against three SARS-CoV-2 variants. *Nat. Commun.* **12**, 3991 (2021). 34183681 10.1038/s41467-021-24285-4 PMC8239026

[57] Sheikh-Mohamed, S. et al. Systemic and mucosal IgA responses are variably induced in response to SARS-CoV-2 mRNA vaccination and are associated with protection against subsequent infection. *Mucosal Immunol.* **15**, 799–808 (2022). 35468942 10.1038/s41385-022-00511-0 PMC9037584

[58] Mastroeni, P. & Rossi, O. Antibodies and protection in systemic *Salmonella* infections: do we still have more questions than answers? *Infect. Immun.* **88**, e00219–e00220 (2020). 32601109 10.1128/IAI.00219-20 PMC7504966

[59] Fraley, E. R. et al. Effects of prior infection with SARS-CoV-2 on B cell receptor repertoire response during vaccination. *Vaccines* **10**, 1477 (2022). 36146555 10.3390/vaccines10091477 PMC9506540

[60] Turner, J. S. et al. SARS-CoV-2 mRNA vaccines induce persistent human germinal centre responses. *Nature* **596**, 109–113 (2021). 34182569 10.1038/s41586-021-03738-2 PMC8935394

[61] Wang, Z. et al. mRNA vaccine-elicited antibodies to SARS-CoV-2 and circulating variants. *Nature* **592**, 616–622 (2021). 33567448 10.1038/s41586-021-03324-6 PMC8503938

[62] Yang, J. et al. A vaccine targeting the RBD of the S protein of SARS-CoV-2 induces protective immunity. *Nature* **586**, 572–577 (2020). 32726802 10.1038/s41586-020-2599-8

[63] Dai, L. & Gao, G. F. Viral targets for vaccines against COVID-19. *Nat. Rev. Immunol.* **21**, 73–82 (2021). 33340022 10.1038/s41577-020-00480-0 PMC7747004

[64] Sidney, L. E., Branch, M. J., Dunphy, S. E., Dua, H. S. & Hopkinson, A. Concise review: evidence for CD34 as a common marker for diverse progenitors. *Stem Cells* **32**, 1380–1389 (2014). 24497003 10.1002/stem.1661 PMC4260088

[65] Boisson-Vidal, C., Benslimane-Ahmim, Z., Lokajczyk, A., Heymann, D. & Smadja, D. M. Osteoprotegerin induces CD34^+^ differentiation in endothelial progenitor cells. *Front. Med.* **5**, 331 (2018). 10.3389/fmed.2018.00331 PMC6277572 30538990

[66] Hassanpour, M., Salybekov, A. A., Kobayashi, S. & Asahara, T. CD34 positive cells as endothelial progenitor cells in biology and medicine. *Front. Cell Dev. Biol.* **11**, 1128134 (2023). 37138792 10.3389/fcell.2023.1128134 PMC10150654

[67] Lee, H., Kim, H., Chung, Y., Kim, J. & Yang, H. Thymocyte differentiation is regulated by a change in estradiol levels during the estrous cycle in mouse. *Dev. Reprod.* **17**, 441–449 (2013). 25949161 10.12717/DR.2013.17.4.441 PMC4382948

[68] Vincent, F. B., Saulep-Easton, D., Figgett, W. A., Fairfax, K. A. & Mackay, F. The BAFF/APRIL system: emerging functions beyond B cell biology and autoimmunity. *Cytokine Growth Factor Rev.* **24**, 203–215 (2013). 23684423 10.1016/j.cytogfr.2013.04.003 PMC7108297

[69] Kuley, R. et al. B cell activating factor (BAFF) from neutrophils and dendritic cells is required for protective B cell responses against *Salmonella* Typhimurium infection. *PLoS ONE* **16**, e0259158 (2021). 34705890 10.1371/journal.pone.0259158 PMC8550399

[70] Lang, J. et al. Replacing mouse BAFF with human BAFF does not improve B-cell maturation in hematopoietic humanized mice. *Blood Adv.* **1**, 2729–2741 (2017). 29296925 10.1182/bloodadvances.2017010090 PMC5745134

[71] Crotty, S. T follicular helper cell biology: a decade of discovery and diseases. *Immunity* **50**, 1132–1148 (2019). 31117010 10.1016/j.immuni.2019.04.011 PMC6532429

[72] Mintz, M. A. & Cyster, J. G. T follicular helper cells in germinal center B cell selection and lymphomagenesis. *Immunol. Rev.* **296**, 48–61 (2020). 32412663 10.1111/imr.12860 PMC7817257

[73] Park, H. J., Park, H. S., Lee, J. U., Bothwell, A. L. & Choi, J. M. Gender-specific differences in PPARγ regulation of follicular helper T cell responses with estrogen. *Sci. Rep.* **6**, 28495 (2016). 27335315 10.1038/srep28495 PMC4917844

[74] Cyster, J. G. & Allen, C. D. C. B cell responses: cell interaction dynamics and decisions. *Cell* **177**, 524–540 (2019). 31002794 10.1016/j.cell.2019.03.016 PMC6538279

[75] Bhan, A. et al. Histone methyltransferase EZH2 is transcriptionally induced by estradiol as well as estrogenic endocrine disruptors bisphenol-A and diethylstilbestrol. *J. Mol. Biol.* **426**, 3426–3441 (2014). 25088689 10.1016/j.jmb.2014.07.025

[76] Zheng, Y. et al. Human neutrophil development and functionality are enabled in a humanized mouse model. *Proc. Natl Acad. Sci. USA* **119**, e2121077119 (2022). 36269862 10.1073/pnas.2121077119 PMC9618085

[77] Dupuis, M. et al. Effects of estrogens on platelets and megakaryocytes. *Int. J. Mol. Sci.* **20**, 3111 (2019). 31242705 10.3390/ijms20123111 PMC6627332

[78] Xu, Y., Zhou, H., Post, G., Zan, H. & Casali, P. Rad52 mediates class-switch DNA recombination to IgD. *Nat. Commun.* **13**, 980 (2022). 35190531 10.1038/s41467-022-28576-2 PMC8861003

[79] Tabor, D. E. & Gould, K. A. Estrogen receptor alpha promotes lupus in (NZBxNZW)F1 mice in a B cell intrinsic manner. *Clin. Immunol.* **174**, 41–52 (2017). 27989899 10.1016/j.clim.2016.10.011 PMC5316311

[80] Soldin, O. P. et al. Steroid hormone levels in pregnancy and 1 year postpartum using isotope dilution tandem mass spectrometry. *Fertil. Steril.* **84**, 701–710 (2005). 16169406 10.1016/j.fertnstert.2005.02.045 PMC3640374

[81] Stricker, R. et al. Establishment of detailed reference values for luteinizing hormone, follicle stimulating hormone, estradiol, and progesterone during different phases of the menstrual cycle on the Abbott ARCHITECT analyzer. *Clin. Chem. Lab. Med.* **44**, 883–887 (2006). 16776638 10.1515/CCLM.2006.160

[82] Sluss, P. M. et al. Mass spectrometric and physiological validation of a sensitive, automated, direct immunoassay for serum estradiol using the Architect. *Clin. Chim. Acta* **388**, 99–105 (2008). 18023274 10.1016/j.cca.2007.10.020

[83] Ingberg, E., Theodorsson, A., Theodorsson, E. & Strom, J. O. Methods for long-term 17β-estradiol administration to mice. *Gen. Comp. Endocrinol.* **175**, 188–193 (2012). 22137913 10.1016/j.ygcen.2011.11.014

[84] Zenclussen, M. L., Casalis, P. A., Jensen, F., Woidacki, K. & Zenclussen, A. C. Hormonal fluctuations during the estrous cycle modulate heme oxygenase-1 expression in the uterus. *Front. Endocrinol.* **5**, 32 (2014). 10.3389/fendo.2014.00032 PMC3952397 24659985

[85] Verdonk, S. J. E. et al. Estradiol reference intervals in women during the menstrual cycle, postmenopausal women and men using an LC-MS/MS method. *Clin. Chim. Acta* **495**, 198–204 (2019). 30981845 10.1016/j.cca.2019.04.062

[86] Varghese, M. et al. Sex hormones regulate metainflammation in diet-induced obesity in mice. *J. Biol. Chem.* **297**, 101229 (2021). 34599964 10.1016/j.jbc.2021.101229 PMC8526779

[87] Ueki, Y. et al. Clonal analysis of a human antibody response. Quantitation of precursors of antibody-producing cells and generation and characterization of monoclonal IgM, IgG, and IgA to rabies virus. *J. Exp. Med.* **171**, 19–34 (1990). 2153188 10.1084/jem.171.1.19 PMC2187652

[88] Ikematsu, H., Harindranath, N., Ueki, Y., Notkins, A. L. & Casali, P. Clonal analysis of a human antibody response. II. Sequences of the VH genes of human IgM, IgG, and IgA to rabies virus reveal preferential utilization of VHIII segments and somatic hypermutation. *J. Immunol.* **150**, 1325–1337 (1993). 8432980 PMC4667541

[89] Kasaian, M. T., Ikematsu, H., Balow, J. E. & Casali, P. Structure of the VH and VL segments of monoreactive and polyreactive IgA autoantibodies to DNA in patients with systemic lupus erythematosus. *J. Immunol.* **152**, 3137–3151 (1994). 8144908 PMC4631053

[90] Ikematsu, H., Ichiyoshi, Y., Schettino, E. W., Nakamura, M. & Casali, P. VH and VL segment structure of anti-insulin IgG autoantibodies in patients with insulin-dependent diabetes mellitus. Evidence for somatic selection. *J. Immunol.* **152**, 1430–1441 (1994). 8301143 PMC4631048

[91] Ichiyoshi, Y. & Casali, P. Analysis of the structural correlates for antibody polyreactivity by multiple reassortments of chimeric human immunoglobulin heavy and light chain V segments. *J. Exp. Med.* **180**, 885–895 (1994). 8064239 10.1084/jem.180.3.885 PMC2191637

[92] Lefranc, M. P. Antibody Informatics: IMGT, the International ImMunoGeneTics Information System. *Microbiol. Spectr.*10.1128/microbiolspec.AID-0001-2012 (2014). 10.1128/microbiolspec.AID-0001-2012 26105823

[93] Grainger, D. J. et al. The serum concentration of active transforming growth factor-β is severely depressed in advanced atherosclerosis. *Nat. Med.* **1**, 74–79 (1995). 7584958 10.1038/nm0195-74

[94] Koyama, T. et al. Raised serum APRIL levels in patients with systemic lupus erythematosus. *Ann. Rheum. Dis.* **64**, 1065–1067 (2005). 15576416 10.1136/ard.2004.022491 PMC1755547

[95] Kim, H. O., Kim, H. S., Youn, J. C., Shin, E. C. & Park, S. Serum cytokine profiles in healthy young and elderly population assessed using multiplexed bead-based immunoassays. *J. Transl. Med* **9**, 113 (2011). 21774806 10.1186/1479-5876-9-113 PMC3146842

[96] Poorbaugh, J. et al. Measurement of IL-21 in human serum and plasma using ultrasensitive MSD S-PLEX(R) and Quanterix SiMoA methodologies. *J. Immunol. Methods* **466**, 9–16 (2019). 30590020 10.1016/j.jim.2018.12.005

[97] Han, H. et al. Profiling serum cytokines in COVID-19 patients reveals IL-6 and IL-10 are disease severity predictors. *Emerg. Microbes Infect.* **9**, 1123–1130 (2020). 32475230 10.1080/22221751.2020.1770129 PMC7473317

[98] Eslami, M. et al. BAFF 60-mer, and differential BAFF 60-mer dissociating activities in human serum, cord blood and cerebrospinal fluid. *Front. Cell Dev. Biol.* **8**, 577662 (2020). 33240880 10.3389/fcell.2020.577662 PMC7677505

