# Supplementary material for: A humanized mouse that mounts mature class-switched, hypermutated and neutralizing antibody responses
Source: Nat Immunol. 2024 Jun 25;25(8):1489–506. doi: 10.1038/s41590-024-01880-3 (PMC11291283; doi:10.1038/s41590-024-01880-3)
Supplement: Supplementary file 1 — Supplementary Figs. 1 and 2 and Supplementary Tables 1–17. [file 41590_2024_1880_MOESM1_ESM.pdf]

nature immunology

Supplementary information https://doi.org/10.1038/s41590-024-01880-3

A humanized mouse that mounts mature class-switched, hypermutated and neutralizing antibody responses

In the format provided by the authors and unedited

Supplementary Information
Table-of-contents

Supplementary Fig. 1 | Flow cytometry gating of human and mouse CD45$^+$ cells, RBCs and platelets

Supplementary Fig. 2 | Human B and T cells in blood, spleen and lymph nodes in humanized mice.

Supplementary Table 1 | Serum 17β-estradiol concentrations in female and male humanized mice.

Supplementary Table 2 | CyTOF analysis: Maxpar Direct Immune Profiling Assay Panel.

Supplementary Table 3 | CyTOF analysis: Human immune lymphoid and myeloid cell markers.

Supplementary Table 4a | CyTOF analysis: THX mouse 354 spleen cells (human markers).

Supplementary Table 4b | CyTOF analysis: THX mouse 355 spleen cells (human markers).

Supplementary Table 4c | CyTOF analysis: THX mouse 356 spleen cells (human markers).

Supplementary Table 4d | CyTOF analysis: THX mouse 357 spleen cells (human markers).

Supplementary Table 4e | CyTOF analysis: THX mouse 358 spleen cells (human markers).

Supplementary Table 4f | CyTOF analysis: THX mice spleen cells (human markers mean values)

Supplementary Table 4g | CyTOF analysis: Human immune cells in THX mice spleens and humans spleens.

Supplementary Table 5a | Circulating human and mouse platelets in THX mice.

Supplementary Table 5b | Circulating human and mouse RBCs in THX mice.

Supplementary Table 6 | Serum human cytokines in flagellin-vaccinated THX mice.

Supplementary Table 7 | Serum human cytokines in COVID-19 mRNA-vaccinated THX mice.

Supplementary Table 8 | JAX NSG huCD34™ mice.

Supplementary Table 9 | Origins of cord bloods source of huCD34$^+$ cells.

Supplementary Table 10 | Humanized NSG mice.

Supplementary Table 11 | Non-grafted NBSGW mice and humanized NBSGW mice.

Supplementary Table 12 | THX mice.

Supplementary Table 13 | Antibodies used in fluorescence flow cytometry and microscopy.

Supplementary Table 14 | Healthy human subjects source of PBMCs, B cells, T cells and sera.

Supplementary Table 15 | Antibodies used in ELISAs, ELISPOTs and cell isolation.

Supplementary Table 16 | PCR primers used in RT-PCR, qRT-PCR and RT-5'RACE PCR.

Supplementary Table 17 | Lupus THX mice.

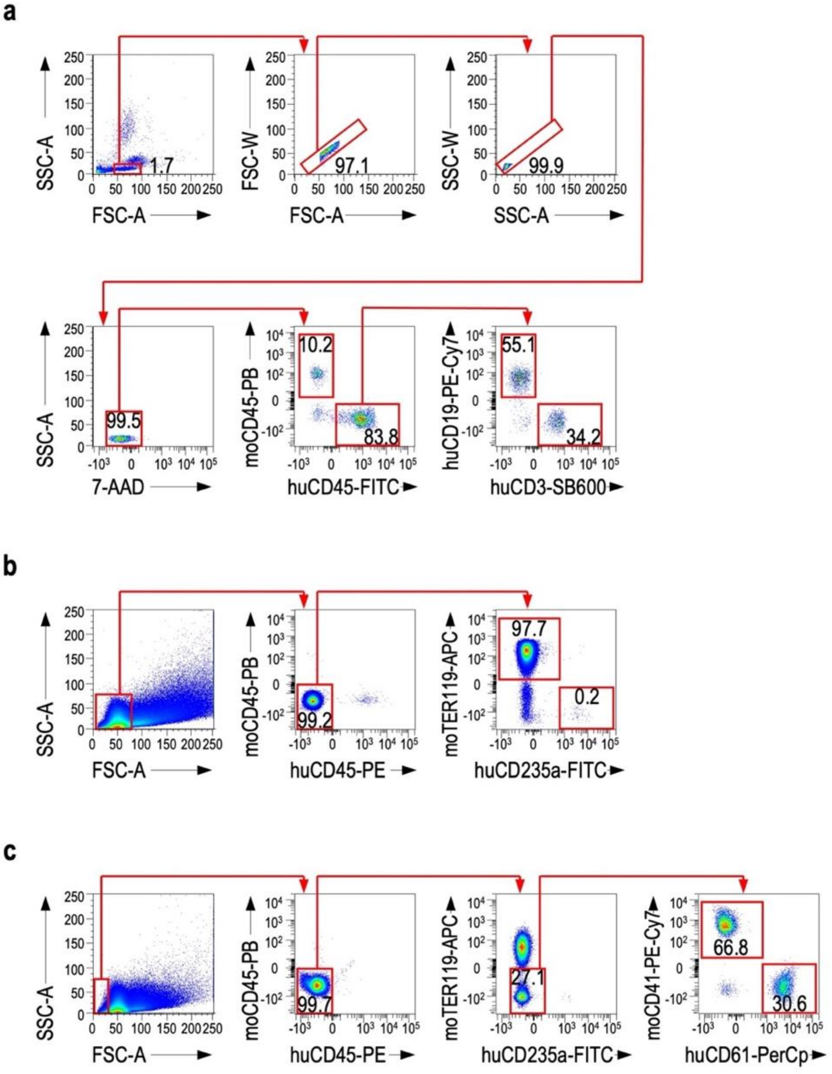

Supplementary Fig. 1 | Flow cytometry gating of human and mouse CD45$^+$ cells, RBCs and platelets. a, huCD45$^+$ and moCD45$^+$ cells, as identified in erythrocyte-depleted peripheral blood of one 24-week-old THX mouse. Erythrocyte-depleted cells were gated for PBMCs, followed by doublet exclusion, and analyzed for live huCD45$^+$ and moCD45$^+$ cells. huCD45$^+$ cells account for 89.1% of total CD45$^+$ cells (huCD45$^+$ plus moCD45$^+$ cells). huCD45$^+$ cells were then analyzed for identification of huCD19$^+$ and huCD3$^+$ cells. **b**,**c** huCD235a$^+$ and moTER119$^+$ RBCs (**b**) and huCD45$^-$CD235a$^-$CD61$^+$ and moCD45$^-$TER119$^-$CD41$^+$ platelets (**c**), as identified in 5 mM EDTA blood of one 20-week-old THX mouse. Flow cytometry plots are from one THX mouse representative of 15 THX mice.

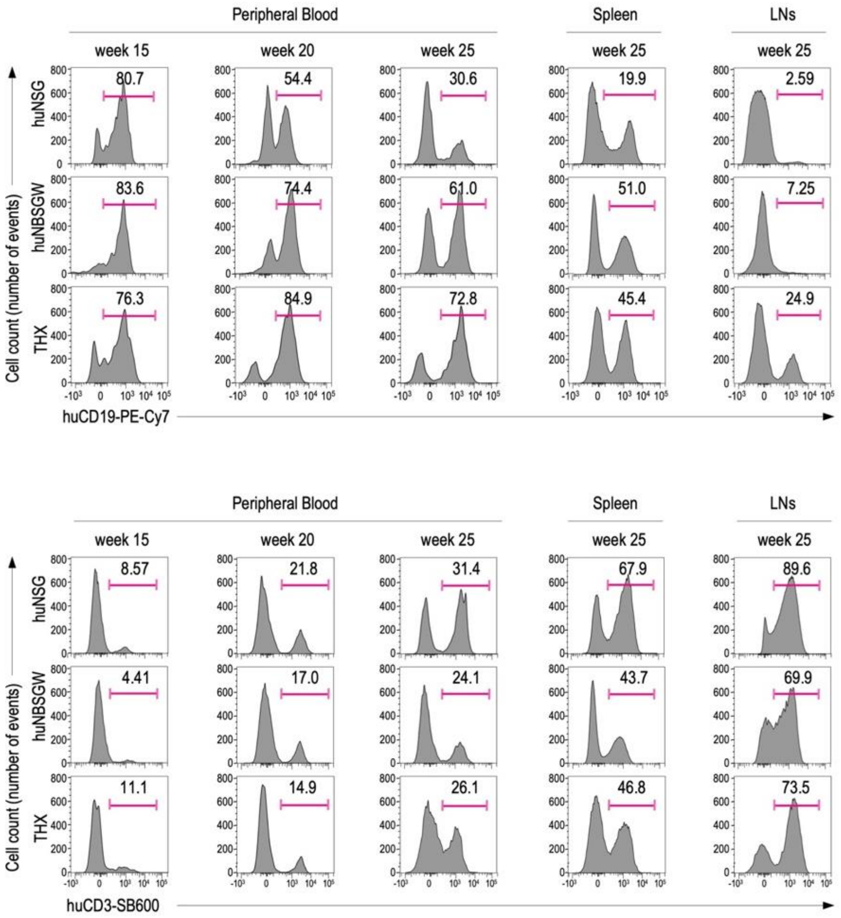

Supplementary Fig. 2 | Human B and T cells in blood, spleen and lymph nodes in humanized mice. huCD19^{+}B cells and huCD3^{+}T cells in blood, spleen and mesenteric LNs of non-intentionally immunized THX, huNBSGW and huNSG mice. Numbers are percentage of huCD45^{+} mononuclear cells. Flow cytometry plots are from one THX, one huNBSGW and one huNSG mouse, each representative of 3 mice.

Supplementary Table 1 | Serum 17β-estradiol concentrations in female and male humanized mice.

Female huNBSGW mice		Female THX mice		Male huNBSGW		Male THX mice	
Estradiol (pg/ml)		Estradiol (pg/ml)		Estradiol (pg/ml)		Estradiol (pg/ml)	
huNBSGW 264	28.85	THX 372	102.95	huNBSGW 276	< 5.00	THX 384	125.74
huNBSGW 265	26.23	THX 373	64.84	huNBSGW 277	< 5.00	THX 385	91.50
huNBSGW 266	13.11	THX 374	111.09	huNBSGW 278	< 5.00	THX 386	94.81
huNBSGW 267	10.62	THX 375	109.70	huNBSGW 279	< 5.00	THX 387	105.60
huNBSGW 268	30.47	THX 376	169.09	huNBSGW 280	< 5.00	THX 388	79.40
huNBSGW 269	26.29	THX 377	51.01	huNBSGW 281	< 5.00	THX 389	62.15
huNBSGW 270	25.01	THX 378	85.99	huNBSGW 282	< 5.00	THX 390	72.75
huNBSGW 271	18.21	THX 379	53.86	huNBSGW 283	< 5.00	THX 391	82.64
huNBSGW 272	19.45	THX 380	54.48	huNBSGW 284	< 5.00	THX 392	88.29
huNBSGW 273	21.21	THX 381	55.12	huNBSGW 285	< 5.00	THX 393	59.61
huNBSGW 274	13.29	THX 382	50.93	huNBSGW 286	< 5.00	THX 394	63.89
huNBSGW 275	18.49	THX 383	76.96	huNBSGW 287	< 5.00	THX 395	66.59
20.94 ± 1.88<br>mean ± s.e.m.		82.17 ± 10.36<br>mean ± s.e.m.		< 5.00<br>mean		82.75 ± 5.72<br>mean ± s.e.m.	

The normal blood estradiol concentration in mice can vary depending on factors such as age, sex and stage of the estrous cycle in females. In female mice, blood estradiol concentration range is as follows: Proestrus (the stage just before estrus), 5–60 pg/ml; Estrus (the stage when ovulation occurs), 15–200 pg/ml; Metestrus (the stage just after estrus), 5–50 pg/ml; Diestrus (the stage between metestrus and proestrus), 5–40 pg/ml. In male mice, blood estradiol concentrations are lower (< 5.0 pg/ml). In the titration method used, estradiol concentrations lower than 5.0 pg/ml were not reliable enough for meaningful measurement. In women, blood estradiol concentration range is: Follicular phase (days 1–14 of the menstrual cycle), 35–400 pg/ml; Mid-cycle (around day 14 of the menstrual cycle), 100–500 pg/ml; Luteal phase (days 14–28 of the menstrual cycle), 35–400 pg/ml; Postmenopausal women, less than 10–30 pg/ml. In pregnant women, blood estradiol concentration range is as follows: First trimester, 300–3,000 pg/ml; Second trimester, 1,900–10,000 pg/ml; Third trimester, 2,000–14,000 pg/ml. In men, blood estradiol concentration range is 10–30 pg/ml. It is important to note that estradiol concentration ranges may vary depending on the laboratory that performs the test and the assay used for measurement. Blood estradiol concentration ranges reported here were derived from multiple sources, as detailed below.

Ingberg, E., Theodorsson, A., Theodorsson, E. & Strom, J.O. Methods for long-term 17β-estradiol administration to mice. *Gen Comp Endocrinol* **175**, 188-193 (2012).

Zenclussen, M.L., Casalis, P.A., Jensen, F., Woidacki, K. & Zenclussen, A.C. Hormonal fluctuations during the estrous cycle modulate heme oxygenase-1 expression in the uterus. *Front Endocrinol* **5**, 32 (2014).

Varghese, M. et al. Sex hormones regulate metainflammation in diet-induced obesity in mice. *J Biol Chem* 297, 101229 (2021).

Soldin, O.P., Guo, T., Weiderpass, E., Tractenberg, R., Hilakivi-Clarke, L. & Soldin, S.J. Steroid hormone levels in pregnancy and 1 year postpartum using isotope dilution tandem mass spectrometry. *Fertil Steril* **84**, 701-710 (2005).

Stricker, R., Eberhart, R., Chevailler, M.C., Quinn, F., Bischof, P. & Stricker, R. Establishment of detailed reference values for luteinizing hormone, follicle stimulating hormone, estradiol, and progesterone during different phases of the menstrual cycle on the Abbot ARCHITECT® analyzer. *Clin Chem Lab Med* **44**, 883-887 (2006).

Sluss, P.M., Hayes, F.J., Adams, J.M., Barnes, W., Williams, G., Frost, S., Ramp, J., Pacenti, D., Lehotay, D.C., George, S., Ramsay, C., Doss, R.C. & Crowley Jr, W.F. Mass spectrometric and physiological validation of a sensitive, automated, direct immunoassay for serum estradiol using the Architect®. *Clin Chim Acta* **388**, 99-105 (2008).

Verdonk, S.J.E., Vesper, H.W., Martens, F., Sluss, P.M., Hillebrand, J.J. & Heijboer, A.C. Estradiol reference intervals in women during the menstrual cycle, postmenopausal women and men using an LC-MS/MS method. *Clin Chim Acta* **495**, 198-204 (2019).

Supplementary Table 2 | CyTOF analysis: Maxpar Direct Immune Profiling Assay Panel.

Antibody	Clone	Mass and Metal Tag
anti–huCD45 mAb	HI30	89Y
anti–huCD196/CCR6 mAb	G034E3	141Pr
anti–huCD123/IL-3R mAb	6H6	143Nd
anti–huCD19 mAb	HIB19	144Nd
anti–huCD4 mAb	RPA-T4	145Nd
anti–huCD8a mAb	RPA-T8	146Nd
anti–huCD11c mAb	Bu15	147Sm
anti–huCD16 mAb	3G8	148Nd
anti–huCD45RO mAb	UCHL1	149Sm
anti–huCD45RA mAb	HI100	150Nd
anti–huCD161 mAb	HP-3G10	151Eu
anti–huCD194 (CCR4) mAb	L291H4	152Sm
anti–huCD25/IL-2Ra mAb	BC96	153Eu
anti–huCD27 mAb	O323	154Sm
anti–huCD57 mAb	HCD57	155Gd
anti–huCD183/CXCR3 mAb	G025H7	156Gd
anti–huCD185/CXCR5 mAb	J252D4	158Gd
anti–huCD28 mAb	CD28.2	160Gd
anti–huCD38 mAb	HB7	161Dy
anti–huCD56/NCAM mAb	NCAM16.2	163Dy
anti–huTCRgd mAb	B1	164Dy
anti–huCD294 mAb	BM16	166Er
anti–huCD197/CCR7 mAb	G043H7	167Er
anti–huCD14 mAb	63D3	168Er
anti–huCD3 mAb	UCHT1	170Er
anti–huCD20 mAb	2H7	171Yb
anti–huCD66b mAb	G10F5	172Yb
anti–huHLA-DR mAb	LN3	173Yb
anti–huIgD mAb	IA6-2	174Yb
anti–huCD127/IL-27Ra mAb	A019D5	176Yb
Live/dead intercalator-103Rh	N/A	103 Rh

The Maxpar Direct Immune Profiling Assay (Fluidigm) system utilizes a 30-marker antibody panel to quantify 37 human immune cell populations in huPBMCs and whole blood by CyTOF. Labeled cells are analyzed by a Helios™ mass cytometer and human immune cell population frequencies, QC metrics and data plot displays are acquired using Maxpar Pathsetter software (The University of Texas Health Science Center at San Antonio Bioanalytics and Single-Cell Core Facility).

Supplementary Table 3 | CyTOF analysis: Human immune lymphoid and myeloid cell markers.

Human cells	Human surface markers
**Lymphocytes**	
CD3 T cells	CD3^{+}CD19^{-}
CD8 T cells	CD3^{+}CD4^{-}CD8^{+}CD19^{-}CD66b^{-}CD14^{-}CD161^{-}TCR$\gamma\delta^{-}$CD123^{-}CD11c^{-}
Naïve (T8nv)	CD3^{+}CD8^{+}CCR7^{+}CD27^{+}CD28^{+}CD45RA^{+}CD45RO^{-}CD16^{-}
Central memory (T8cm)	CD3^{+}CD8^{+}CCR7^{+}CD27^{+}CD16^{-}
Effector memory (T8em)	CD3^{+}CD8^{+}CCR7^{-}CD27^{-}CD38^{-}
Terminal effector (T8te)	CD3^{+}CD8^{+}CCR7^{-}CD27^{-}CD38^{-}
CD4 T cells	CD3^{+}CD4^{+}CD8^{-}CD19^{-}CD66b^{-}CD14^{-}TCR$\gamma\delta^{-}$CD11c^{-}
Naïve (T4nv)	CD3^{+}CD4^{+}CCR7^{+}CD45RA^{+}CD45RO^{-}CCR6^{-}HLA-DR^{-}
Central memory (T4cm)	CD3^{+}CD4^{+}CD127^{+}
Effector memory (T4em)	CD3^{+}CD4^{+}CD45RA^{-}CD45RO^{+}CXCR3^{+}
Terminal effector (T4te)	CD3^{+}CD4^{+}CD45RA^{-}CD45RO^{+}CXCR3^{+}
Treg	CD4^{+}CD25^{+}CCR4^{+}CD127^{-}
Th1-like	CD4^{+}CXCR3^{+}CCR6^{-}CXCR5^{-}
Th2-like	CD4^{+}CCR4^{+}CXCR3^{-}CCR6^{-}CXCR5^{-}
Th17-like	CD4^{+}CCR4^{+}CXCR3^{-}CCR6^{+}CXCR5^{-}
Gamma Delta T cells (GDs)	CD3^{+}CD4^{-}CD8^{lo}TCR$\gamma\delta^{+}$CD66b^{-}CD14^{-}
NKT & MAIT cells	CD3^{+}CD4^{-}CD28^{+}CD161^{+}TCR$\gamma\delta^{+}$CD66b^{-}CD14^{-}CD16^{-}
B cells	CD19^{+}CD20^{+}HLA-DR^{+}
Naïve (B Naïve)	CD19^{+}IgD^{+}
Memory (B Mem)	CD19^{+}CD27^{+}IgD^{-}
Plasmablasts (PB)	CD19^{+}CD27^{+}CD38^{++}
NK cells	
Early NK	CD16^{+}CD56^{+}CD57^{-}CD45RA^{+}CD3^{-}CD14^{-}CD28^{-}CD66b^{-}CD123^{-}
Late NK	CD16^{+}CD56^{+}CD57^{+}CD45RA^{+}CD3^{-}CD14^{-}CD28^{-}CD66b^{-}CD123^{-}
**Monocytes**	
Classical (Class Mono)	CD11c^{+}HLA-DR^{+}CD14^{+}CD38^{+}CD16^{-}CD3^{-}CD19^{-}CD56^{-}CD66b^{-}
Transitional (Int)	CD11c^{+}HLA-DR^{+}CD14^{hi}CD16^{+}CD3^{-}CD19^{-}CD56^{-}CD66b^{-}
Non-classical (NC Mono)	CD11c^{+}HLA-DR^{+}CD14^{lo}CD16^{+}CD3^{-}CD19^{-}CD56^{-}CD66b^{-}
**Dendritic cells**	
plasmacytoid (pDCs)	CD123^{+}HLA-DR^{+}CD3^{-}CD11c^{-}CD14^{-}CD19^{-}CD20^{-}CD66b^{-}
myeloid (mDCs)	CD123^{-}HLA-DR^{+}CD3^{-}CD11c^{+}CD14^{-}CD16^{lo}CD38^{lo}CD19^{-}CD20^{-}CD294^{-}
**Granulocytes**	
Neutrophils (activated)	CD33^{+}CD66b^{+}CD16^{+}HLA-DR^{-}
Basophils	CD33^{-}CD66b^{-}CD16^{+}HLA-DR^{-}CD38^{+}CD123^{+}CD294^{+}
Eosinophils	CD33^{-}CD66b^{+}HLA-DR^{-}CD3^{-}CD14^{-}CD19^{-}CD294^{+}
Neutrophils (resting)	CD33^{+}CD66b^{-}CD16^{+}HLA-DR^{-}

NKT (Natural killer T cells, CD4^{-}) and MAIT (mucosal-associated invariant T cells, CD4^{-}).

Supplementary Table 4a | CyTOF analysis: THX mouse 354 spleen cells (human markers).

	Cell count	% Intact live cells	Parent cells	% Parent cells
Intact live cells	167262	100	All events	92.35
**Lymphocytes**	129130	77.20	Intact live	**77.20**
CD3 T cells	65134	38.94	Lymphocytes	50.44
CD8 T cells	8598	5.14	CD3 T cells	13.20
Naïve (T8nv)	2558	1.53	CD8 T cells	29.75
Central memory (T8cm)	1188	0.71	CD8 T cells	13.82
Effector memory (T8em)	4688	2.80	CD8 T cells	54.52
Terminal effector (T8te)	164	0.10	CD8 T cells	1.91
CD4 T cells	55490	33.18	CD3 T cells	85.19
Naïve (T4nv)	1705	1.02	CD4 T cells	3.07
Central memory (T4cm)	10233	6.12	CD4 T cells	18.44
Effector memory (T4em)	15758	9.42	CD4 T cells	28.40
Terminal effector (T4te)	27794	16.62	CD4 T cells	50.09
Treg	3352	2.00	CD4 T cells	6.04
Th1-like	4622	2.76	CD4 T cells	8.33
Th2-like	3572	2.14	CD4 T cells	6.44
Th17-like	905	0.54	CD4 T cells	1.63
Gamma Delta T cells (GDs)	345	0.21	CD3 T cells	0.53
NKT & MAIT cells	701	0.42	CD3 T cells	1.08
B cells	56385	33.71	Lymphocytes	43.67
Naïve (B Naïve)	54870	32.80	B cells	97.31
Memory (B Mem)	783	0.47	B cells	1.39
Plasmablasts (PB)	732	0.44	B cells	1.30
NK cells	7611	4.55	Lymphocytes	5.89
Early NK	7607	4.55	NK cells	99.95
Late NK	4	0.00	NK cells	0.05
**Monocytes**	500	0.30	Intact live	**0.30**
Classical (Class Mono)	378	0.23	Monocytes	75.60
Transitional (Int)	67	0.04	Monocytes	13.40
Non-classical (NC Mono)	55	0.03	Monocytes	11
**Dendritic cells**	423	0.25	Intact live	**0.25**
plasmacytoid (pDCs)	12	0.01	Dendritic cells	2.84
myeloid (mDCs)	411	0.25	Dendritic cells	97.16
**Granulocytes**	24420	14.60	Intact live	**14.60**
Neutrophils (activated)	180	0.11	Granulocytes	0.74
Basophils	0	0.00	Granulocytes	0
Eosinophils	0	0.00	Granulocytes	0
Neutrophils (resting)	24240	14.49	Granulocytes	99.26
**Others**	12789	N/A	All events	**7.65**

NKT (Natural killer T cells, $CD4^-$) and MAIT (mucosal-associated invariant T cells, $CD4^-$).

Supplementary Table 4b | CyTOF analysis: THX mouse 355 spleen cells (human markers).

	Cell count	% Intact live cells	Parent cells	% Parent cells
Intact live cells	193923	100	All events	88.16
**Lymphocytes**	144220	74.37	Intact live	**74.37**
CD3 T cells	67709	34.92	Lymphocytes	46.95
CD8 T cells	17237	8.89	CD3 T cells	25.46
Naïve (T8nv)	6237	3.22	CD8 T cells	36.18
Central memory (T8cm)	1462	0.75	CD8 T cells	8.48
Effector memory (T8em)	8897	4.59	CD8 T cells	51.62
Terminal effector (T8te)	641	0.33	CD8 T cells	3.72
CD4 T cells	49309	25.43	CD3 T cells	72.82
Naïve (T4nv)	2987	1.54	CD4 T cells	6.06
Central memory (T4cm)	11495	5.93	CD4 T cells	23.31
Effector memory (T4em)	27862	14.37	CD4 T cells	56.50
Terminal effector (T4te)	6965	3.59	CD4 T cells	14.13
Treg	4584	2.36	CD4 T cells	9.30
Th1-like	2062	1.06	CD4 T cells	4.18
Th2-like	2773	1.43	CD4 T cells	5.62
Th17-like	855	0.44	CD4 T cells	1.73
Gamma Delta T cells (GDs)	439	0.23	CD3 T cells	0.65
NKT & MAIT cells	724	0.37	CD3 T cells	1.07
B cells	67149	34.63	Lymphocytes	46.56
Naïve (B Naïve)	64761	33.40	B cells	96.44
Memory (B Mem)	1141	0.59	B cells	1.70
Plasmablasts (PB)	1247	0.64	B cells	1.86
NK cells	9362	4.83	Lymphocytes	6.49
Early NK	9354	4.82	NK cells	99.91
Late NK	8	0.00	NK cells	0.09
**Monocytes**	1628	0.84	Intact live	**0.84**
Classical (Class Mono)	1317	0.68	Monocytes	80.90
Transitional (Int)	213	0.11	Monocytes	13.08
Non-classical (NC Mono)	98	0.05	Monocytes	6.02
**Dendritic cells**	388	0.20	Intact live	**0.20**
plasmacytoid (pDCs)	113	0.06	Dendritic cells	29.12
myeloid (mDCs)	275	0.14	Dendritic cells	70.88
**Granulocytes**	24731	12.75	Intact live	**12.75**
Neutrophils (activated)	536	0.28	Granulocytes	2.17
Basophils	6	0.00	Granulocytes	0.02
Eosinophils	13	0.01	Granulocytes	0.05
Neutrophils (resting)	24176	12.47	Granulocytes	97.76
**Others**	22956	N/A	All events	**11.84**

NKT (Natural killer T cells, $CD4^{-}$) and MAIT (mucosal-associated invariant T cells, $CD4^{-}$).

Supplementary Table 4c | CyTOF analysis: THX mouse 356 spleen cells (human markers).

	Cell count	% Intact live cells	Parent cells	% Parent cells
Intact live cells	167317	100	All events	85.51
**Lymphocytes**	88726	53.03	Intact live	**53.03**
CD3 T cells	39116	23.38	Lymphocytes	44.09
CD8 T cells	10666	6.37	CD3 T cells	27.27
Naïve (T8nv)	6462	3.86	CD8 T cells	60.59
Central memory (T8cm)	1502	0.90	CD8 T cells	14.08
Effector memory (T8em)	2300	1.37	CD8 T cells	21.56
Terminal effector (T8te)	402	0.24	CD8 T cells	3.77
CD4 T cells	27652	16.53	CD3 T cells	70.69
Naïve (T4nv)	5710	3.41	CD4 T cells	20.65
Central memory (T4cm)	4637	2.77	CD4 T cells	16.77
Effector memory (T4em)	13004	7.77	CD4 T cells	47.03
Terminal effector (T4te)	4301	2.57	CD4 T cells	15.55
Treg	2280	1.36	CD4 T cells	8.25
Th1-like	1198	0.72	CD4 T cells	4.33
Th2-like	3765	2.25	CD4 T cells	13.62
Th17-like	1800	1.08	CD4 T cells	6.51
Gamma Delta T cells (GDs)	74	0.04	CD3 T cells	0.19
NKT & MAIT cells	724	0.43	CD3 T cells	1.85
B cells	46663	27.89	Lymphocytes	52.59
Naïve (B Naïve)	44986	26.89	B cells	96.41
Memory (B Mem)	1553	0.93	B cells	3.33
Plasmablasts (PB)	124	0.07	B cells	0.27
NK cells	2947	1.76	Lymphocytes	3.32
Early NK	2936	1.75	NK cells	99.63
Late NK	11	0.01	NK cells	0.37
**Monocytes**	2412	1.44	Intact live	**1.44**
Classical (Class Mono)	2175	1.30	Monocytes	90.17
Transitional (Int)	190	0.11	Monocytes	7.88
Non-classical (NC Mono)	47	0.03	Monocytes	1.95
**Dendritic cells**	452	0.27	Intact live	**0.27**
plasmacytoid (pDCs)	88	0.05	Dendritic cells	19.47
myeloid (mDCs)	364	0.22	Dendritic cells	80.53
**Granulocytes**	51481	30.77	Intact live	**30.77**
Neutrophils (activated)	54	0.03	Granulocytes	0.10
Basophils	0	0.00	Granulocytes	0.00
Eosinophils	0	0.00	Granulocytes	0.00
Neutrophils (resting)	51427	30.74	Granulocytes	99.90
**Others**	24246	N/A	All events	**14.49**

NKT (Natural killer T cells, $CD4^{-}$) and MAIT (mucosal-associated invariant T cells, $CD4^{-}$).

Supplementary Table 4d | CyTOF analysis: THX mouse 357 spleen cells (human markers).

	Cell count	% Intact live cells	Parent cells	% Parent cells
Intact live cells	145249	100	All events	83.73
**Lymphocytes**	63075	43.43	Intact live	**43.43**
CD3 T cells	36792	25.33	Lymphocytes	58.36
CD8 T cells	9273	6.38	CD3 T cells	25.20
Naïve (T8nv)	332	0.23	CD8 T cells	3.58
Central memory (T8cm)	667	0.46	CD8 T cells	7.19
Effector memory (T8em)	8186	5.64	CD8 T cells	88.28
Terminal effector (T8te)	88	0.06	CD8 T cells	0.95
CD4 T cells	26156	18.01	CD3 T cells	71.09
Naïve (T4nv)	406	0.28	CD4 T cells	1.55
Central memory (T4cm)	767	0.53	CD4 T cells	2.93
Effector memory (T4em)	17008	11.71	CD4 T cells	65.03
Terminal effector (T4te)	7975	5.49	CD4 T cells	30.49
Treg	1714	1.18	CD4 T cells	6.55
Th1-like	1014	0.70	CD4 T cells	3.88
Th2-like	6942	4.78	CD4 T cells	26.54
Th17-like	659	0.45	CD4 T cells	2.52
Gamma Delta T cells (GDs)	86	0.06	CD3 T cells	0.23
NKT & MAIT cells	1277	0.88	CD3 T cells	3.47
B cells	24523	16.88	Lymphocytes	38.88
Naïve (B Naïve)	24292	16.79	B cells	99.47
Memory (B Mem)	70	0.05	B cells	0.29
Plasmablasts (PB)	60	0.04	B cells	0.24
NK cells	1760	1.21	Lymphocytes	2.79
Early NK	1699	1.17	NK cells	95.53
Late NK	61	0.04	NK cells	3.47
**Monocytes**	1072	0.74	Intact live	**0.74**
Classical (Class Mono)	867	0.60	Monocytes	80.88
Transitional (Int)	127	0.09	Monocytes	11.85
Non-classical (NC Mono)	78	0.05	Monocytes	7.28
**Dendritic cells**	498	0.34	Intact live	**0.34**
plasmacytoid (pDCs)	53	0.04	Dendritic cells	10.64
myeloid (mDCs)	445	0.31	Dendritic cells	89.36
**Granulocytes**	56970	39.22	Intact live	**39.22**
Neutrophils (activated)	143	0.10	Granulocytes	0.25
Basophils	55	0.04	Granulocytes	0.10
Eosinophils	57	0.04	Granulocytes	0.10
Neutrophils (resting)	567715	39.05	Granulocytes	99.55
**Others**	23634	N/A	All events	**16.27**

NKT (Natural killer T cells, CD4$^-$) and MAIT (mucosal-associated invariant T cells, CD4$^-$).

Supplementary Table 4e | CyTOF analysis: THX mouse 358 spleen cells (human markers).

	Cell count	% Intact live cells	Parent cells	% Parent cells
Intact live cells	197262	100	All events	85.68
**Lymphocytes**	154666	78.41	Intact live	**78.41**
CD3 T cells	71174	36.08	Lymphocytes	46.02
CD8 T cells	14224	7.21	CD3 T cells	19.98
Naïve (T8nv)	4411	2.24	CD8 T cells	31.01
Central memory (T8cm)	1502	0.76	CD8 T cells	10.56
Effector memory (T8em)	6306	3.20	CD8 T cells	44.33
Terminal effector (T8te)	2005	1.02	CD8 T cells	14.10
CD4 T cells	56207	28.49	CD3 T cells	78.97
Naïve (T4nv)	2144	1.09	CD4 T cells	3.81
Central memory (T4cm)	9791	4.96	CD4 T cells	17.42
Effector memory (T4em)	8885	4.50	CD4 T cells	15.81
Terminal effector (T4te)	35387	17.94	CD4 T cells	62.96
Treg	2728	1.38	CD4 T cells	4.85
Th1-like	6223	3.15	CD4 T cells	11.07
Th2-like	6889	3.49	CD4 T cells	12.26
Th17-like	915	0.46	CD4 T cells	1.63
Gamma Delta T cells (GDs)	569	0.29	CD3 T cells	0.80
NKT & MAIT cells	174	0.09	CD3 T cells	0.24
B cells	75792	38.42	Lymphocytes	49.00
Naïve (B Naïve)	75018	38.03	B cells	98.98
Memory (B Mem)	590	0.30	B cells	0.78
Plasmablasts (PB)	184	0.09	B cells	0.24
NK cells	7700	3.90	Lymphocytes	4.98
Early NK	7693	3.90	NK cells	99.91
Late NK	7	0.00	NK cells	0.09
**Monocytes**	2760	1.40	Intact live	**1.40**
Classical (Class Mono)	2094	1.06	Monocytes	75.87
Transitional (Int)	365	0.19	Monocytes	13.22
Non-classical (NC Mono)	301	0.15	Monocytes	10.91
**Dendritic cells**	150	0.08	Intact live	**0.08**
plasmacytoid (pDCs)	62	0.03	Dendritic cells	41.33
myeloid (mDCs)	88	0.04	Dendritic cells	58.67
**Granulocytes**	11432	5.80	Intact live	**5.80**
Neutrophils (activated)	30	0.02	Granulocytes	0.26
Basophils	3	0.00	Granulocytes	0.03
Eosinophils	9	0.00	Granulocytes	0.08
Neutrophils (resting)	11390	5.77	Granulocytes	99.63
**Others**	28254	N/A	All events	**14.32**

NKT (Natural killer T cells, $CD4^{-}$) and MAIT (mucosal-associated invariant T cells, $CD4^{-}$).

Supplementary Table 4f | CyTOF analysis: THX mouse 354, 355. 356, 357, 358 spleen cells (human markers mean values).

	THX 354	THX 355	THX 356	THX 357	THX 358	mean ± s.e.m.
Intact live cells	92.35	88.16	85.51	83.73	85.68	87.09 ± 1.49
**Lymphocytes**	77.20	74.37	53.03	43.43	78.41	**65.29 ± 7.16**
CD3 T cells	50.44	46.95	44.09	58.36	46.02	49.17 ± 2.52
CD8 T cells	13.20	25.46	27.27	25.20	19.98	22.22 ± 2.56
Naïve (T8nv)	29.75	36.18	60.59	3.58	31.01	32.22 ± 9.08
Central memory (T8cm)	13.82	8.48	14.08	7.19	10.56	10.83 ± 1.39
Effector memory (T8em)	54.52	51.62	21.56	88.28	44.33	52.06 ± 10.74
Terminal effector (T8te)	1.91	3.72	3.77	0.95	14.10	4.89 ± 2.37
CD4 T cells	85.19	72.82	70.69	71.09	78.97	75.75 ± 2.79
Naïve (T4nv)	3.07	6.06	20.65	1.55	3.81	7.03 ± 3.48
Central memory (T4cm)	18.44	23.31	16.77	2.93	17.42	15.77 ± 3.41
Effector memory (T4em)	28.40	56.50	47.03	65.03	15.81	42.55 ± 9.04
Terminal effector (T4te)	50.09	14.13	15.55	30.49	62.96	34.64 ± 9.60
Treg	6.04	9.30	8.25	6.55	4.85	6.99 ± 0.79
Th1-like	8.33	4.18	4.33	3.88	11.07	6.36 ± 1.43
Th2-like	6.44	5.62	13.62	26.54	12.26	12.90 ± 3.75
Th17-like	1.63	1.73	6.51	2.52	1.63	2.80 ± 0.94
Gamma Delta T cells	0.53	0.65	0.19	0.23	0.80	0.48 ± 0.12
NKT & MAIT cells	1.08	1.07	1.85	3.47	0.24	1.54 ± 0.55
B cells	43.67	46.56	52.59	38.88	49.00	46.14 ± 2.33
Naïve	97.31	96.44	96.41	99.47	98.98	97.72 ± 0.64
Memory	1.39	1.70	3.33	0.29	0.78	1.50 ± 0.52
Plasmablasts (PB)	1.30	1.86	0.27	0.24	0.24	0.78 ± 0.34
NK cells	5.89	6.49	3.32	2.79	4.98	4.69 ± 0.72
Early NK	99.95	99.91	99.63	95.53	99.91	98.99 ± 0.87
Late NK	0.05	0.09	0.37	3.47	0.09	0.81 ± 0.67
**Monocytes**	0.30	0.84	1.44	0.74	1.40	**0.94 ± 0.22**
Classical (Class Mono)	75.60	80.90	90.17	80.88	75.87	80.68 ± 2.64
Transitional (Int)	13.40	13.08	7.88	11.85	13.22	11.89 ± 1.04
Non-classical (NC Mono)	11	6.02	1.95	7.28	10.91	7.43 ± 1.69
**Dendritic cells**	0.25	0.20	0.27	0.34	0.08	**0.23 ± 0.04**
plasmacytoid (pDCs)	2.84	29.12	19.47	10.64	41.33	20.68 ± 6.78
myeloid (mDCs)	97.16	70.88	80.53	89.36	58.67	79.32 ± 6.78
**Granulocytes**	14.60	12.75	30.77	39.22	5.80	**20.63 ± 6.19**
Neutrophils (activated)	0.74	2.17	0.10	0.25	0.26	0.70 ± 0.38
Basophils	0	0.02	0.00	0.10	0.03	0.03 ± 0.02
Eosinophils	0	0.05	0.00	0.10	0.08	0.05 ± 0.02
Neutrophils (resting)	99.26	97.76	99.90	99.55	99.63	99.22 ± 0.38
**Others**	7.65	11.84	14.49	16.27	14.32	**12.91 ± 1.49**

NKT (Natural killer T cells, $CD4^-$) and MAIT (mucosal-associated invariant T cells, $CD4^-$).

Supplementary Table 4g | CyTOF analysis: THX mice spleen human immune (lymphoid and myeloid) cells are comparable in proportion to humans spleen human lymphoid and myeloid cells.

$huCD45^+$ white cells (%)	THX 354	THX 355	THX 356	THX 357	THX 358	THX mice (n=5) spleen	Adult humans (n=6) spleen*
huB cells	36.50	39.28	32.62	20.16	44.84	34.68 ± 4.14	37.15 ± 2.14
$huCD4^+$ T cells	35.93	28.86	19.33	21.51	33.25	27.78 ± 3.23	20.16 ± 1.90
$huCD8^+$ T cells	5.56	10.08	7.45	7.62	8.42	7.83 ± 0.73	11.49 ± 2.27
huNK cells	4.93	5.48	2.06	1.45	4.55	3.69 ± 0.81	5.63 ± 1.42
huNKT cells	0.68	0.66	0.56	1.12	0.45	0.70 ± 0.12	1.62 ± 0.39
huDCs	0.27	0.23	0.32	0.41	0.09	0.26 ± 0.05	0.32 ± 0.02
Human monocytes	0.32	0.95	1.68	0.88	1.63	1.10 ± 0.25	1.88 ± 0.72
Human neutrophils (resting and activated)	15.81	14.46	35.98	46.76	6.76	23.95 ± 7.47	21.70 ± 4.31

Proportions of lymphoid and myeloid $huCD45^+$ cells in spleen of non-intentionally immunized THX mice (*n*=5, same mice as in **Fig. 1f**) and humans (*n*=6, 25 to 66-year-old)* who died from accidental death, as analyzed by CyTOF and flow cytometry, respectively. Data are presented as mean ± s.e.m. *Proportions of huB cells, huT cells, huNK cells, huDCs, human monocytes and neutrophils in spleen of humans are reported in Gualdron-Lopez, M., *et al.* Multiparameter flow cytometry analysis of the human spleen applied to studies of plasma-derived EVs from *Plasmodium vivax* patients and healthy controls. *Front Cell Infect Microbiol* **11**, 596104 (2021). Approximate mean ± s.e.m. values were derived from Fig. 1c of the publication.

Supplementary Table 5a | Circulating human and mouse platelets in THX mice.

THX mouse	Sex	huCD61$^+$ platelets (% total platelets)		moCD41$^+$ platelets (% total platelets)	
THX mouse 514	M	31.48		68.52	
THX mouse 515	F	23.79		76.21	
THX mouse 516	M	31.48		68.52	
THX mouse 517	F	22.38		77.62	
THX mouse 518	F	41.41		58.59	
THX mouse 519	F	23.96		76.04	
THX mouse 520	F	32.56		67.44	
THX mouse 521	M	36.34	mean ± s.e.m. 32.60 ± 2.25	63.66	mean ± s.e.m. 67.40 ± 2.25
THX mouse 522	M	27.50		72.50	
THX mouse 523	M	24.59		75.41	
THX mouse 524	M	29.41		70.59	
THX mouse 525	F	30.33		69.67	
THX mouse 526	F	41.41		58.59	
THX mouse 527	F	37.36		62.64	
THX mouse 528	F	55.07		44.93	

Identification of huCD45$^-$CD235a$^-$CD61$^+$ and moCD45$^-$TER119$^-$ CD41$^+$ platelets in 5mM EDTA blood of THX mice (*n*=15) by flow cytometry. Numbers are percentage of total (human plus mouse) platelets.

Supplementary Table 5b | Circulating human and mouse RBCs in THX mice.

THX mouse	Sex	huCD235a$^+$ RBCs (% total RBCs)		moTER119$^+$ RBCs (% total RBCs)	
THX mouse 514	M	0.19		99.81	
THX mouse 515	F	0.25		99.75	
THX mouse 516	M	0.18		99.82	
THX mouse 517	F	0.27		99.73	
THX mouse 518	F	0.13		99.87	
THX mouse 519	F	0.24		99.76	
THX mouse 520	F	0.24		99.76	
THX mouse 521	M	0.13	mean ± s.e.m. 0.20 ± 0.01	99.87	mean ± s.e.m. 99.8 ± 0.01
THX mouse 522	M	0.15		99.85	
THX mouse 523	M	0.14		99.86	
THX mouse 524	M	0.16		99.84	
THX mouse 525	F	0.19		99.81	
THX mouse 526	F	0.22		99.78	
THX mouse 527	F	0.18		99.82	
THX mouse 528	F	0.16		99.84	

Identification of huCD235a$^+$ and moTER119$^+$ RBCs in 5mM EDTA blood of THX mice (*n*=15) by flow cytometry. Numbers are percentage of total (human plus mouse) RBCs.

Supplementary Table 6 | Serum human cytokines in flagellin-vaccinated THX mice.

THX mouse	Sex	Vaccine	huAPRIL (pg/ml)	huBAFF (pg/ml)	huTGF-β1 (pg/ml)	huIFN-γ (pg/ml)	huIL-2 (pg/ml)	huIL-4 (pg/ml)	huIL-6 (pg/ml)	huIL-10 (pg/ml)	huIL-21 (pg/ml)
THX 450	M	Flagellin	383.7	372.8	9151	24.74	2.16	8.80	4.58	4.17	< 0.10
THX 451	M	Flagellin	112.0	121.9	2966	0.17	0.50	3.55	1.71	1.67	< 0.10
THX 452	F	Flagellin	141.2	133.3	8678	3.22	1.94	N.D.	6.06	2.29	< 0.10
THX 453	F	Flagellin	129.1	172.8	12345	0.12	0.99	6.90	1.78	1.15	< 0.10
THX 454	M	Flagellin	219.7	244.7	7636	4.16	0.66	7.58	0.40	2.34	< 0.10
THX 460	M	Flagellin	231.9	335.5	8535	0.19	0.44	8.12	1.71	2.53	< 0.10
THX 461	M	Flagellin	238.0	275.8	3451	4.00	0.57	6.13	0.62	1.21	< 0.10
THX 462	F	Flagellin	210.4	207.4	5383	2.27	0.88	6.44	1.93	1.96	< 0.10
THX 463	F	Flagellin	169.0	184.1	5179	N.D.	N.D.	3.55	0.40	0.84	< 0.10
THX 464	F	Flagellin	217.9	212.5	9839	7.30	0.68	6.13	3.62	0.38	< 0.10
THX 529	M	Flagellin	211.6	199.4	9900	0.16	0.41	4.69	1.16	1.36	< 0.10
THX 530	F	Flagellin	197.9	246.1	10744	13.55	0.77	4.69	21.61	4.47	< 0.10
THX 531	F	Flagellin	223.1	269.7	2629	3.22	0.60	7.58	0.62	0.49	< 0.10
THX 532	F	Flagellin	173.4	209.0	2825	N.D.	N.D.	4.69	1.42	0.62	< 0.10
THX 533	M	Flagellin	215.3	256.7	7902	N.D.	N.D.	6.76	0.40	2.80	< 0.10
THX 534	M	Flagellin	206.3	247.8	10455	2.27	1.30	8.80	1.78	1.26	< 0.10
Mean ± s.e.m.			205 ± 15.20	231 ± 16.50	7351 ± 794	5.03 ± 1.94	0.91 ± 0.16	6.29 ± 0.45	3.11 ± 1.30	1.85 ± 0.30	< 0.10

Serum human cytokine concentrations in THX mice vaccinated with flagellin (*n*=16). In healthy adult humans, the approximate concentrations (range) of circulating cytokines are as follows: huAPRIL, 100–400 pg/ml; huBAFF, 50–400 pg/ml; huTGF-β1, 1000–10,000 pg/ml; huIFN-γ, 0.1–4.2 pg/ml; huIL-2, 0.1–2.0 pg/ml; huIL-4, 0.5–4.0 pg/ml; huIL-6, 0.1–5.0 pg/ml; huIL-10, 0.1–2.8 pg/ml; huIL-21, < 0.1 pg/ml. N.D., not determined. It is important to note that human cytokine concentration ranges may vary depending on the type of assay used for measurement. The human cytokine concentration ranges reported here were derived from multiple sources, as detailed below.

Grainger, D.J., Kemp, P.R., Metcalfe, J.C., Liu, A.C., Lawn, R.M., Williams, N.R., Grace, A.A., Schofield, P.M. & Chauhan, A. The serum concentration of active transforming growth factor-β is severely depressed in advanced atherosclerosis. *Nat Med* **1**, 74-79 (1995).

Koyama, T., Tsukamoto, H., Miyagi, Y., Himeji, D., Otsuka, J., Miyagawa, H., Harada, M. & Horiuchi, T. Raised serum APRIL levels in patients with systemic lupus erythematosus. *Ann Rheum Dis* **64**, 1065-1067 (2005).

Kim, H.O., Kim, H.S., Youn, J.C., Shin, E.C. & Park, S. Serum cytokine profiles in healthy young and elderly population assessed using multiplexed bead-based immunoassays. *J Transl Med* **9**, 113 (2011).

Poorbaugh, J., Samanta, T., Bright, S.W., Sissons, S.E., Chang, C.Y., Oberoi, P., MacDonald, A.J., Martin, A.P., Cox, K.L. & Benschop, R.J. Measurement of IL-21 in human serum and plasma using ultrasensitive MSD S-PLEX(R) and Quanterix SiMoA methodologies. *J Immunol Methods* **466**, 9-16 (2019).

Han, H., Ma, Q., Li, C., Liu, R., Zhao, L., Wang, W., Zhang, P., Liu, X., Gao, G., Liu, F., Jiang, Y., Cheng, X., Zhu, C. & Xia, Y. Profiling serum cytokines in COVID-19 patients reveals IL-6 and IL-10 are disease severity predictors. *Emerg Microbes Infect* **9**, 1123-1130 (2020).

Eslami, M., Meinl, E., Eibel, H., Willen, L., Donze, O., Distl, O., Schneider, H., Speiser, D.E., Tsiantoulas, D., Yalkinoglu, O., Samy, E. & Schneider, P. BAFF 60-mer, and differential BAFF 60-mer dissociating activities in human serum, cord blood and cerebrospinal fluid. *Front Cell Dev Biol* **8**, 577662 (2020).

All above THX mice were vaccinated with flagellin according to the vaccination schedule described in Fig. 6a and used as a source of serum for titration of cytokines. THX mice 450–454 were used for titration of antibodies, in vitro *S*. Typhimurium neutralization, cell analyses and molecular genetic analysis. THX mice 460–464 were used for *S*. Typhimurium infection survival analysis. THX mice 529–534 were used for titration of antibodies.

Supplementary Table 7 | Serum human cytokines in COVID-19 mRNA-vaccinated THX mice.

THX mouse	Sex	Pfizer COVID-19 Vaccine	huAPRIL (pg/ml)	huBAFF (pg/ml)	huTGF-β1 pg/ml)	huIFN-γ (pg/ml)	huIL-2 (pg/ml)	huIL-4 (pg/ml)	huIL-6 (pg/ml)	huIL-10 (pg/ml)	huIL-21 (pg/ml)
THX 473	M	mRNA	295.5	288.4	7544	9.49	2.05	8.80	2.15	2.15	< 0.10
THX 474	F	mRNA	97.6	121.0	10495	0.15	0.41	2.42	0.41	1.06	< 0.10
THX 475	M	mRNA	243.0	231.5	6606	11.36	1.09	8.12	3.69	5.44	< 0.10
THX 476	F	mRNA	96.4	101.4	8063	0.12	0.66	2.42	0.40	0.73	< 0.10
THX 477	M	mRNA	272.5	302.2	7572	6.52	1.52	6.13	2.70	5.72	< 0.10
THX 478	F	mRNA	105.5	114.2	7712	0.19	0.88	2.42	0.33	0.49	< 0.10
THX 479	M	mRNA	281.7	317.9	7646	5.42	0.88	10.90	4.91	5.31	< 0.10
THX 480	F	mRNA	117.4	132.1	7037	0.13	0.51	6.13	0.47	0.84	< 0.10
THX 493	M	mRNA	121.8	126.7	8403	N.D.	0.56	3.55	N.D.	0.62	< 0.10
THX 494	M	mRNA	81.2	93.8	7441	0.17	0.61	2.42	0.25	1.57	< 0.10
THX 495	F	mRNA	137.4	165.6	7782	3.22	0.58	3.55	13.06	2.80	< 0.10
THX 496	F	mRNA	174.8	193.7	10231	127.4	0.42	N.D.	1.93	N.D.	< 0.10
THX 497	F	mRNA	192.5	202.3	6922	92.55	1.09	N.D.	1.38	0.49	< 0.10
THX 498	F	mRNA	173.8	168.4	3963	53.24	N.D.	2.42	1.93	N.D.	< 0.10
THX 535	F	mRNA	164.0	157.4	1053	26.14	0.49	3.55	23.15	4.09	< 0.10
THX 536	F	mRNA	265.0	258.2	4919	0.11	0.63	3.55	0.62	0.90	< 0.10
THX 537	F	mRNA	110.5	116.1	10187	0.16	0.51	4.69	0.10	0.38	< 0.10
THX 538	F	mRNA	99.7	111.9	7754	2.90	0.64	2.64	0.23	0.49	< 0.10
THX 539	M	mRNA	108.5	104.8	7339	7.77	0.43	2.42	0.10	0.62	< 0.10
THX 540	M	mRNA	130.1	126.7	7426	10.27	N.D.	N.D.	N.D.	N.D.	< 0.10
THX 541	M	mRNA	162.3	137.0	16446	N.D.	N.D.	N.D.	N.D.	0.25	< 0.10
THX 542	F	mRNA	195.0	220.4	5241	2.74	2.05	3.55	0.32	N.D.	< 0.10
Mean ± s.e.m.			164 ± 14.45	172 ± 14.86	7627 ± 610	18.00 ± 7.66	0.84 ± 0.12	4.43 ± 0.60	3.06 ± 1.31	1.89 ± 0.45	< 0.10

Serum human cytokine concentrations in THX mice vaccinated with Pfizer-BioNTech 162b2 COVID-19 mRNA (*n*=22). In healthy adult humans, the approximate concentrations (range) of circulating cytokines are as follows: huAPRIL, 100–400 pg/ml; huBAFF, 50–400 pg/ml; huTGF-β1, 1000–10,000 pg/ml; huIFN-γ, 0.1–4.2 pg/ml; huIL-2, 0.1–2.0 pg/ml; huIL-4, 0.5–4.0 pg/ml; huIL-6, 0.1–5.0 pg/ml; huIL-10, 0.1–2.8 pg/ml; huIL-21, < 0.1 pg/ml. N.D., not determined. It is important to note that human cytokine concentration ranges may vary depending on the type of assay used for measurement. The human cytokine concentration ranges reported here were derived from multiple sources, as detailed in Supplementary Table 6.

All above THX mice were vaccinated with Pfizer-BioNTech 162b2 COVID-19 mRNA according to human vaccination schedule and used as a source of serum for titration of cytokines. THX mice 473–480 were used for titration of antibodies, in vitro RBD competition assays, cell analyses and molecular genetic analysis. THX mice 493–498 and 535–542 were used for titration of antibodies.

Supplementary Table 8 | JAX NSG huCD34™ mice.

huMouse		Sex	Age	Immunization	Mouse or mouse cells used in experiments of:
JAX NSG huCD34™	601	F	23 weeks	NP-CGG	Fig. 4a,b and Fig. 5a–c,g,h
JAX NSG huCD34™	602	F	23 weeks	NP-CGG	Fig. 4a,b and Fig. 5a–c,g,h
JAX NSG huCD34™	603	F	23 weeks	NP-CGG	Fig. 4a,b and Fig. 5a–c,g,h
JAX NSG huCD34™	604	F	23 weeks	NP-CGG	Fig. 4a and Fig. 5a,f
JAX NSG huCD34™	605	F	23 weeks	DNP-CpG	Fig. 4g,h
JAX NSG huCD34™	606	F	23 weeks	DNP-CpG	Fig. 4g,h
JAX NSG huCD34™	607	F	23 weeks	DNP-CpG	Fig. 4g,h
JAX NSG huCD34™	608	F	23 weeks	DNP-CpG	Fig. 4g

JAX NSG huCD34™ mice were purchased from The Jackson Laboratory (JAX NSG huCD34™ mice were constructed by grafting γ-irradiated female NSG mice at 3 weeks of age with cord blood huCD34$^+$ cells).

Supplementary Table 9 | Origins of cord bloods source of huCD34⁺ cells.

Newborn	Sex	Race	Mother's age range	Cord blood volume (ml)	huCD34⁺ cells
01	M	Latino	31–35	19	1.25×10^5
02	M	Latino	41–45	21	8.25×10^5
03	F	Latino	20–25	24	2.60×10^6
04	F	Latino	20–25	18	7.50×10^5
05	M	Latino	36–40	36	1.20×10^6
06	F	White	31–35	15	3.10×10^5
07	F	Latino	31–35	25	1.24×10^6
08	F	Latino	26–30	28	9.72×10^5
09	M	Black	20–25	40	8.50×10^5
10	F	Latino	31–35	36	2.15×10^6
11	F	Latino	26–30	18	1.22×10^6
12	M	Latino	31–35	34	2.74×10^6
13	M	White	36–40	21	4.83×10^5
14	M	Latino	26–30	24	7.84×10^5
15	M	Latino	36–40	30	1.67×10^6
16	M	Latino	26–30	34	1.76×10^6
17	F	Latino	31–35	51	1.76×10^6
18	M	Latino	36–40	15	6.95×10^5
19	F	Black	31–35	32	1.22×10^6
21	M	Latino	26–30	34	2.63×10^6
22	F	Latino	31–35	15	5.65×10^5
23	F	White	26–30	39	1.74×10^6
24	F	Latino	36–40	25	6.45×10^5
25	F	Black	26–30	40	2.60×10^6
26	F	Latino	31–35	24	9.13×10^5
27	F	Latino	26–30	42	3.50×10^6
28	F	Latino	35–40	20	1.12×10^6
29	F	White	20–25	62	8.00×10^6
30	M	Latino	31–35	42	1.76×10^6
31	M	Latino	36–40	14	6.30×10^5
32	M	Latino	26–30	19	1.05×10^6
33	F	Latino	31–35	30	0.80×10^6
34	M	White	31–35	28	1.42×10^6
35	F	Black	31–35	42	2.47×10^6
36	F	Latino	20–25	22	8.60×10^5
37	F	Latino	31–35	38	9.40×10^5
38	F	Latino	20–25	49	2.2×10^6
39	F	Latino	26–30	45	1.40×10^6
40	F	Latino	31–35	18	0.50×10^6
41	F	Latino	31–35	45	1.80×10^6
43	M	Latino	26–30	40	9.63×10^5
44	F	Latino	41–45	46	2.19×10^6
45	F	Latino	26–30	51	2.34×10^6
46	F	Asian	36–40	36	1.08×10^6
47	F	Black	26–30	42	3.05×10^6
48	M	Black	26–30	44	3.35×10^6
49	M	White	20–25	30	2.78×10^6
50	M	Latino	31–35	45	2.28×10^6

51	M	Latino	20–25	30	2.44×10^6
52	M	White	36–40	26	1.34×10^6
53	F	Latino	46–50	28	1.90×10^6
54	M	Asian	31–35	33	1.84×10^6
55	M	Latino	41–45	35	2.23×10^6
56	M	Latino	26–30	40	2.74×10^6
57	M	White	31–35	42	2.96×10^6
58	F	Latino	26–30	37	2.55×10^6
59	F	Latino	31–35	39	2.88×10^6
60	M	White	31–35	43	3.16×10^6
61	M	Latino	31–35	34	2.38×10^6
62	M	Latino	41–45	38	3.01×10^6
63	M	Latino	26–30	42	1.61×10^6
64	M	Latino	31–35	49	3.36×10^6
65	F	White	31–35	37	1.90×10^6
66	M	Black	31–35	44	2.40×10^6
67	M	Latino	20–25	34	2.01×10^6
68	M	Latino	31–35	32	3.15×10^6
69	F	Latino	31–35	38	3.78×10^6
70	F	Latino	31–35	49	5.54×10^6
71	F	Latino	31–35	46	3.60×10^6
72	M	Latino	26–30	41	2.24×10^6
73	M	Latino	20–25	33	1.35×10^6
74	F	Latino	26–30	28	9.92×10^5
75	M	Latino	31–35	35	2.81×10^6
76	F	Latino	26–30	30	1.67×10^6
77	M	Latino	31–35	36	2.64×10^6
78	F	Latino	26–30	39	2.72×10^6
79	F	Latino	31–35	35	1.44×10^6
80	M	Latino	26–30	32	1.11×10^6
81	F	Latino	36–40	33	1.38×10^6
82	M	Latino	31–35	43	2.57×10^6
83	F	Black	26–30	39	2.29×10^6
84	F	White	26–30	44	2.67×10^6
85	M	Latino	26–30	35	1.98×10^6
86	F	Latino	20–25	40	2.71×10^6
87	M	Latino	26–30	33	1.88×10^6
88	F	Latino	31–35	26	1.13×10^6

The most recent United States census recognized the following racial categories: White, Black, Latino, Asian, Native American or Alaskan Native, and Native Hawaiian or other Pacific Islander.

Supplementary Table 10 | Humanized NSG mice.

huMouse		Sex	Age	Immunization	Mouse or mouse cells used in experiments of:
huNSG	101	F	40 weeks	Non-int. immunized	Fig. 1a,b,e
huNSG	102	F	40 weeks	Non-int. immunized	Fig. 1a,b,e
huNSG	103	M	40 weeks	Non-int. immunized	Fig. 1a,b,e
huNSG	104	M	40 weeks	Non-int. immunized	Fig. 1a,b,e
huNSG	105	M	40 weeks	Non-int. immunized	Fig. 1a,b,e
huNSG	106	M	26 weeks	Non-int. immunized	Fig. 1c,d
huNSG	107	F	26 weeks	Non-int. immunized	Fig. 1d
huNSG	108	F	27 weeks	Non-int. immunized	Fig. 1d
huNSG	109	F	27 weeks	Non-int. immunized	Fig. 1c,d
huNSG	110	M	33 weeks	Non-int. immunized	Fig. 1d
huNSG	111	F	33 weeks	Non-int. immunized	Fig. 1d
huNSG	112	M	34 weeks	Non-int. immunized	Fig. 1d
huNSG	113	M	34 weeks	Non-int. immunized	Fig. 1c,d
huNSG	114	M	34 weeks	Non-int. immunized	Fig. 1d
huNSG	115	F	34 weeks	Non-int. immunized	Fig. 1d
huNSG	116	M	47 weeks	Non-int. immunized	Fig. 1d
huNSG	117	F	47 weeks	Non-int. immunized	Fig. 1c,d
huNSG	118	F	47 weeks	Non-int. immunized	Fig. 1d
huNSG	119	F	53 weeks	Non-int. immunized	Fig. 1c,d
huNSG	120	M	53 weeks	Non-int. immunized	Fig. 1d
huNSG	121	M	55 weeks	Non-int. immunized	Fig. 1c,d
huNSG	122	M	55 weeks	Non-int. immunized	Fig. 1c,d
huNSG	123	F	55 weeks	Non-int. immunized	Fig. 1d
huNSG	124	M	18 weeks	Non-int. immunized	Fig. 1g
huNSG	125	F	18 weeks	Non-int. immunized	Fig. 1g
huNSG	126	M	18 weeks	Non-int. immunized	Fig. 1g
huNSG	127	M	25 weeks	Non-int. immunized	Fig. 1g
huNSG	128	F	25 weeks	Non-int. immunized	Fig. 1g
huNSG	129	M	25 weeks	Non-int. immunized	Fig. 1g
huNSG	130	F	15, 20, 25 weeks	Non-int. immunized	Supplementary Fig. 2
huNSG	131	F	15, 20, 25 weeks	Non-int. immunized	Supplementary Fig. 2
huNSG	132	F	15, 20, 25 weeks	Non-int. immunized	Supplementary Fig. 2

huNSG mice were constructed by preconditioning myeloablation of NSG mouse neonates (48 h of birth) with sublethal (1 Gy) γ-irradiation, followed by intracardiac injection of purified cord blood huCD34^{+} cells (1.5 x 10^{5} freshly isolated of frozen-thawed huCD34^{+} cells in 50 μl PBS supplemented with 2.0% FBS) using a 27-gauge needle.

Supplementary Table 11 | Non-grafted NBSGW mice and humanized NBSGW mice.

Mouse or huMouse		Sex	Age	Immunization	Mouse or mouse cells used in experiments of:
NBSGW	201	M	20 weeks	Non-int. immunized	Ext. Data Fig. 3
NBSGW	202	M	20 weeks	Non-int. immunized	Ext. Data Fig. 3
NBSGW	203	M	20 weeks	Non-int. immunized	Ext. Data Fig. 3
NBSGW	204	F	20 weeks	Non-int. immunized	Ext. Data Fig. 3
NBSGW	205	F	20 weeks	Non-int. immunized	Ext. Data Fig. 3
NBSGW	206	F	20 weeks	Non-int. immunized	Ext. Data Fig. 3
huNBSGW	201	F	40 weeks	Non-int. immunized	Fig. 1a,b,e
huNBSGW	202	F	40 weeks	Non-int. immunized	Fig. 1a,b,e
huNBSGW	203	M	40 weeks	Non-int. immunized	Fig. 1a,b,e
huNBSGW	204	M	40 weeks	Non-int. immunized	Fig. 1a,b,e
huNBSGW	205	M	40 weeks	Non-int. immunized	Fig. 1a,b,e
huNBSGW	206	F	33 weeks	Non-int. immunized	Fig. 1c,d
huNBSGW	207	F	33 weeks	Non-int. immunized	Fig. 1d
huNBSGW	208	M	33 weeks	Non-int. immunized	Fig. 1c,d
huNBSGW	209	M	47 weeks	Non-int. immunized	Fig. 1d
huNBSGW	210	M	47 weeks	Non-int. immunized	Fig. 1d
huNBSGW	211	F	47 weeks	Non-int. immunized	Fig. 1c,d
huNBSGW	212	M	50 weeks	Non-int. immunized	Fig. 1d
huNBSGW	213	M	50 weeks	Non-int. immunized	Fig. 1d
huNBSGW	214	M	50 weeks	Non-int. immunized	Fig. 1c,d
huNBSGW	215	F	50 weeks	Non-int. immunized	Fig. 1c,d
huNBSGW	216	F	50 weeks	Non-int. immunized	Fig. 1d
huNBSGW	217	F	50 weeks	Non-int. immunized	Fig. 1d
huNBSGW	218	F	55 weeks	Non-int. immunized	Fig. 1d
huNBSGW	219	M	55 weeks	Non-int. immunized	Fig. 1d
huNBSGW	220	M	55 weeks	Non-int. immunized	Fig. 1d
huNBSGW	221	M	55 weeks	Non-int. immunized	Fig. 1d
huNBSGW	222	F	55 weeks	Non-int. immunized	Fig. 1d
huNBSGW	223	F	55 weeks	Non-int. immunized	Fig. 1d
huNBSGW	224	M	55 weeks	Non-int. immunized	Fig. 1d
huNBSGW	225	F	55 weeks	Non-int. immunized	Fig. 1d
huNBSGW	226	F	55 weeks	Non-int. immunized	Fig. 1d
huNBSGW	227	M	55 weeks	Non-int. immunized	Fig. 1d
huNBSGW	228	M	55 weeks	Non-int. immunized	Fig. 1d
huNBSGW	229	F	18 weeks	Non-int. immunized	Fig. 1g
huNBSGW	230	M	18 weeks	Non-int. immunized	Fig. 1g
huNBSGW	231	F	18 weeks	Non-int. immunized	Fig. 1g
huNBSGW	232	F	25 weeks	Non-int. immunized	Fig. 1g
huNBSGW	233	M	25 weeks	Non-int. immunized	Fig. 1g

huNBSGW	234	F	25 weeks	Non-int. immunized	Fig. 1g
huNBSGW	235	F	20 weeks	NP-CGG	Fig. 4a–c
huNBSGW	236	F	20 weeks	NP-CGG	Fig. 4a–c
huNBSGW	237	F	20 weeks	NP-CGG	Fig. 4a
huNBSGW	238	M	24 weeks	NP-CGG	Fig. 4a–c
huNBSGW	239	M	24 weeks	NP-CGG	Fig. 4a
huNBSGW	240	M	24 weeks	NP-CGG	Fig. 4a
huNBSGW	241	M	24 weeks	NP-CGG	Fig. 4a
huNBSGW	242	F	20 weeks	NP-CGG	Fig. 5b,c,d,f,g,h
huNBSGW	243	M	20 weeks	NP-CGG	Fig. 5b,c,d,f,g,h
huNBSGW	244	F	20 weeks	NP-CGG	Fig. 5b,c,d,f,g,h
huNBSGW	245	F	20 weeks	NP-CGG	Fig. 5b,g,h
huNBSGW	246	F	20 weeks	NP-CGG	Fig. 5b,g,h,i
huNBSGW	247	M	20 weeks	NP-CGG	Fig. 5b,g,h,i
huNBSGW	248	M	20 weeks	NP-CGG	Fig. 5b,g,h,i
huNBSGW	249	F	20 weeks	NP-CGG	Fig. 5e
huNBSGW	250	M	20 weeks	NP-CGG	Fig. 5e
huNBSGW	251	F	24 weeks	NP-CGG	Fig. 5e
huNBSGW	252	F	24 weeks	DNP-CpG	Fig. 4g,h
huNBSGW	253	F	24 weeks	DNP-CpG	Fig. 4g,h
huNBSGW	254	F	24 weeks	DNP-CpG	Fig. 4g,h
huNBSGW	255	M	20 weeks	DNP-CpG	Fig. 4g
huNBSGW	256	M	20 weeks	DNP-CpG	Fig. 4g
huNBSGW	257	M	20 weeks	DNP-CpG	Fig. 4g
huNBSGW	258	M	20 weeks	DNP-CpG	Fig. 4g,j and Ext. Data Fig. 2b
huNBSGW	259	M	20 weeks	DNP-CpG	Fig. 4j,l,m,o,p and Ext. Data Fig. 2b
huNBSGW	260	F	20 weeks	DNP-CpG	Fig. 4j,l,m,o,p and Ext. Data Fig. 2b
huNBSGW	261	F	20 weeks	DNP-CpG	Fig. 4j,l,m,o,p and Ext. Data Fig. 2b
huNBSGW	262	M	20 weeks	DNP-CpG	Fig. 4j,l,m,o,p and Ext. Data Fig. 2b
huNBSGW	263	F	20 weeks	DNP-CpG	Fig. 4j,l,m,o,p and Ext. Data Fig. 2b
huNBSGW	264	F	18 weeks	Non-int. immunized	Ext. Data Fig. 1 and Supplementary Table 1
huNBSGW	265	F	18 weeks	Non-int. immunized	Ext. Data Fig. 1 and Supplementary Table 1
huNBSGW	266	F	18 weeks	Non-int. immunized	Ext. Data Fig. 1 and Supplementary Table 1
huNBSGW	267	F	18 weeks	Non-int. immunized	Ext. Data Fig. 1 and Supplementary Table 1
huNBSGW	268	F	20 weeks	Non-int. immunized	Ext. Data Fig. 1 and Supplementary Table 1
huNBSGW	269	F	20 weeks	Non-int. immunized	Ext. Data Fig. 1 and Supplementary Table 1
huNBSGW	270	F	20 weeks	Non-int. immunized	Ext. Data Fig. 1 and Supplementary Table 1
huNBSGW	271	F	20 weeks	Non-int. immunized	Ext. Data Fig. 1 and Supplementary Table 1
huNBSGW	272	F	21 weeks	Non-int. immunized	Ext. Data Fig. 1 and Supplementary Table 1
huNBSGW	273	F	21 weeks	Non-int. immunized	Ext. Data Fig. 1 and Supplementary Table 1
huNBSGW	274	F	21 weeks	Non-int. immunized	Ext. Data Fig. 1 and Supplementary Table 1
huNBSGW	275	F	21 weeks	Non-int. immunized	Ext. Data Fig. 1 and Supplementary Table 1

huNBSGW	276	M	23 weeks	Non-int. immunized	Ext. Data Fig. 1 and Supplementary Table 1
huNBSGW	277	M	23 weeks	Non-int. immunized	Ext. Data Fig. 1 and Supplementary Table 1
huNBSGW	278	M	23 weeks	Non-int. immunized	Ext. Data Fig. 1 and Supplementary Table 1
huNBSGW	279	M	23 weeks	Non-int. immunized	Ext. Data Fig. 1 and Supplementary Table 1
huNBSGW	280	M	24 weeks	Non-int. immunized	Ext. Data Fig. 1 and Supplementary Table 1
huNBSGW	281	M	24 weeks	Non-int. immunized	Ext. Data Fig. 1 and Supplementary Table 1
huNBSGW	282	M	24 weeks	Non-int. immunized	Ext. Data Fig. 1 and Supplementary Table 1
huNBSGW	283	M	24 weeks	Non-int. immunized	Ext. Data Fig. 1 and Supplementary Table 1
huNBSGW	284	M	22 weeks	Non-int. immunized	Ext. Data Fig. 1 and Supplementary Table 1
huNBSGW	285	M	22 weeks	Non-int. immunized	Ext. Data Fig. 1 and Supplementary Table 1
huNBSGW	286	M	22 weeks	Non-int. immunized	Ext. Data Fig. 1 and Supplementary Table 1
huNBSGW	287	M	22 weeks	Non-int. immunized	Ext. Data Fig. 1 and Supplementary Table 1
huNBSGW	288	M	15, 20, 25 weeks	Non-int. immunized	Supplementary Fig. 2
huNBSGW	289	F	15, 20, 25 weeks	Non-int. immunized	Supplementary Fig. 2
huNBSGW	290	F	15, 20, 25 weeks	Non-int. immunized	Supplementary Fig. 2
huNBSGW	291	M	20 weeks	Non-int. immunized	Ext. Data Fig. 3
huNBSGW	292	F	20 weeks	Non-int. immunized	Ext. Data Fig. 3
huNBSGW	293	M	20 weeks	Non-int. immunized	Ext. Data Fig. 3
huNBSGW	294	F	20 weeks	Non-int. immunized	Ext. Data Fig. 3
huNBSGW	295	F	20 weeks	Non-int. immunized	Ext. Data Fig. 3
huNBSGW	296	M	20 weeks	Non-int. immunized	Ext. Data Fig. 3
huNBSGW	297	F	18 weeks	Non-int. immunized	Fig. 1a

huNBSGW mice were constructed by grafting non-irradiated NBSGW mouse neonates (48 h of birth) with umbilical cord blood huCD34$^+$ cells (1.5 x 10^5 freshly isolated of frozen-thawed huCD34$^+$ cells in 50 μl PBS supplemented with 2.0% FBS) via intracardiac injection.

Supplementary Table 12 | THX mice.

THX mouse		Sex	Age	Immunization	Mouse or mouse cells used in experiments of:
THX	301	M	40 weeks	Non-int. immunized	Fig. 1a,b,e
THX	302	F	40 weeks	Non-int. immunized	Fig. 1a,b,e
THX	303	F	40 weeks	Non-int. immunized	Fig. 1a,b,e
THX	304	M	40 weeks	Non-int. immunized	Fig. 1a,b,e
THX	305	F	40 weeks	Non-int. immunized	Fig. 1a,b,e
THX	306	M	29 weeks	Non-int. immunized	Fig. 1c,d
THX	307	M	29 weeks	Non-int. immunized	Fig. 1d
THX	308	M	29 weeks	Non-int. immunized	Fig. 1d
THX	309	M	29 weeks	Non-int. immunized	Fig. 1d
THX	310	F	29 weeks	Non-int. immunized	Fig. 1c,d
THX	311	F	29 weeks	Non-int. immunized	Fig. 1d
THX	312	F	55 weeks	Non-int. immunized	Fig. 1d
THX	313	F	55 weeks	Non-int. immunized	Fig. 1d
THX	314	F	55 weeks	Non-int. immunized	Fig. 1d
THX	315	M	55 weeks	Non-int. immunized	Fig. 1d
THX	316	M	55 weeks	Non-int. immunized	Fig. 1d
THX	317	M	55 weeks	Non-int. immunized	Fig. 1d
THX	318	F	55 weeks	Non-int. immunized	Fig. 1c,d
THX	319	M	55 weeks	Non-int. immunized	Fig. 1d
THX	320	M	55 weeks	Non-int. immunized	Fig. 1d
THX	321	F	55 weeks	Non-int. immunized	Fig. 1d
THX	322	M	55 weeks	Non-int. immunized	Fig. 1c,d
THX	323	M	55 weeks	Non-int. immunized	Fig. 1d
THX	324	M	55 weeks	Non-int. immunized	Fig. 1d
THX	325	F	55 weeks	Non-int. immunized	Fig. 1d
THX	326	M	55 weeks	Non-int. immunized	Fig. 1c,d
THX	327	F	55 weeks	Non-int. immunized	Fig. 1d
THX	328	F	55 weeks	Non-int. immunized	Fig. 1d
THX	329	F	55 weeks	Non-int. immunized	Fig. 1d
THX	330	F	55 weeks	Non-int. immunized	Fig. 1c,d
THX	331	M	55 weeks	Non-int. immunized	Fig. 1d
THX	332	M	55 weeks	Non-int. immunized	Fig. 1d
THX	333	F	55 weeks	Non-int. immunized	Fig. 1d
THX	334	F	55 weeks	Non-int. immunized	Fig. 1d
THX	335	M	55 weeks	Non-int. immunized	Fig. 1d
THX	336	M	55 weeks	Non-int. immunized	Fig. 1d
THX	337	M	55 weeks	Non-int. immunized	Fig. 1d
THX	338	F	55 weeks	Non-int. immunized	Fig. 1d
THX	339	M	55 weeks	Non-int. immunized	Fig. 1d
THX	340	F	55 weeks	Non-int. immunized	Fig. 1d
THX	341	F	55 weeks	Non-int. immunized	Fig. 1d
THX	342	F	55 weeks	Non-int. immunized	Fig. 1d
THX	343	M	55 weeks	Non-int. immunized	Fig. 1d
THX	344	M	55 weeks	Non-int. immunized	Fig. 1d
THX	345	F	55 weeks	Non-int. immunized	Fig. 1d

THX	346	M	55 weeks	Non-int. immunized	Fig. 1d
THX	347	F	55 weeks	Non-int. immunized	Fig. 1d
THX	348	F	55 weeks	Non-int. immunized	Fig. 1d
THX	349	M	55 weeks	Non-int. immunized	Fig. 1d
THX	350	M	55 weeks	Non-int. immunized	Fig. 1d
THX	351	F	55 weeks	Non-int. immunized	Fig. 1d
THX	352	M	55 weeks	Non-int. immunized	Fig. 1d
THX	353	F	55 weeks	Non-int. immunized	Fig. 1d
THX	354	M	20 weeks	Non-int. immunized	Fig. 1f and Ext. Data Table 1
THX	355	F	20 weeks	Non-int. immunized	Fig. 1f and Ext. Data Table 1
THX	356	M	20 weeks	Non-int. immunized	Fig. 1f and Ext. Data Table 1
THX	357	F	20 weeks	Non-int. immunized	Fig. 1f and Ext. Data Table 1
THX	358	M	20 weeks	Non-int. immunized	Fig. 1f and Ext. Data Table 1
THX	359	F	18 weeks	Non-int. immunized	Fig. 1g
THX	360	F	18 weeks	Non-int. immunized	Fig. 1g
THX	361	F	18 weeks	Non-int. immunized	Fig. 1g
THX	362	F	25 weeks	Non-int. immunized	Fig. 1g
THX	363	F	25 weeks	Non-int. immunized	Fig. 1g
THX	364	F	25 weeks	Non-int. immunized	Fig. 1g
THX	365	M	20 weeks	Non-int. immunized	Fig. 2a–f and Ext. Data Fig. 3
THX	366	F	20 weeks	Non-int. immunized	Fig. 2a–f and Ext. Data Fig. 3
THX	367	M	20 weeks	Non-int. immunized	Fig. 2a–f and Ext. Data Fig. 3
THX	368	F	20 weeks	Non-int. immunized	Not used
THX	369	F	20 weeks	Non-int. immunized	Fig. 3a–e
THX	370	F	20 weeks	Non-int. immunized	Fig. 3a–e
THX	371	M	20 weeks	Non-int. immunized	Fig. 3a–e
THX	372	F	20 weeks	Non-int. immunized	Ext. Data Fig. 1 and Supplementary Table 1
THX	373	F	20 weeks	Non-int. immunized	Ext. Data Fig. 1 and Supplementary Table 1
THX	374	F	20 weeks	Non-int. immunized	Ext. Data Fig. 1 and Supplementary Table 1
THX	375	F	20 weeks	Non-int. immunized	Ext. Data Fig. 1 and Supplementary Table 1
THX	376	F	20 weeks	Non-int. immunized	Ext. Data Fig. 1 and Supplementary Table 1
THX	377	F	20 weeks	Non-int. immunized	Ext. Data Fig. 1 and Supplementary Table 1
THX	378	F	20 weeks	Non-int. immunized	Ext. Data Fig. 1 and Supplementary Table 1
THX	379	F	20 weeks	Non-int. immunized	Ext. Data Fig. 1 and Supplementary Table 1
THX	380	F	20 weeks	Non-int. immunized	Ext. Data Fig. 1 and Supplementary Table 1
THX	381	F	20 weeks	Non-int. immunized	Ext. Data Fig. 1 and Supplementary Table 1
THX	382	F	20 weeks	Non-int. immunized	Ext. Data Fig. 1 and Supplementary Table 1
THX	383	F	20 weeks	Non-int. immunized	Ext. Data Fig. 1 and Supplementary Table 1
THX	384	M	20 weeks	Non-int. immunized	Ext. Data Fig. 1 and Supplementary Table 1
THX	385	M	20 weeks	Non-int. immunized	Ext. Data Fig. 1 and Supplementary Table 1
THX	386	M	21 weeks	Non-int. immunized	Ext. Data Fig. 1 and Supplementary Table 1
THX	387	M	21 weeks	Non-int. immunized	Ext. Data Fig. 1 and Supplementary Table 1
THX	388	M	21 weeks	Non-int. immunized	Ext. Data Fig. 1 and Supplementary Table 1
THX	389	M	21 weeks	Non-int. immunized	Ext. Data Fig. 1 and Supplementary Table 1
THX	390	M	21 weeks	Non-int. immunized	Ext. Data Fig. 1 and Supplementary Table 1
THX	391	M	22 weeks	Non-int. immunized	Ext. Data Fig. 1 and Supplementary Table 1
THX	392	M	22 weeks	Non-int. immunized	Ext. Data Fig. 1 and Supplementary Table 1

THX	393	M	22 weeks	Non-int. immunized	Ext. Data Fig. 1 and Supplementary Table 1
THX	394	M	22 weeks	Non-int. immunized	Ext. Data Fig. 1 and Supplementary Table 1
THX	395	M	22 weeks	Non-int. immunized	Ext. Data Fig. 1 and Supplementary Table 1
THX	396	M	15, 20, 25 weeks	Non-int. immunized	Supplementary Fig. 2
THX	397	M	15, 20, 25 weeks	Non-int. immunized	Supplementary Fig. 2
THX	398	F	15, 20, 25 weeks	Non-int. immunized	Supplementary Fig. 2
THX	399	F	20 weeks	Non-int. immunized	Ext. Data Fig. 3
THX	400	F	20 weeks	Non-int. immunized	Not used
THX	401	F	20 weeks	Non-int. immunized	Not used
THX	402	F	20 weeks	Non-int. immunized	Ext. Data Fig. 3
THX	403	M	20 weeks	Non-int. immunized	Ext. Data Fig. 3
THX	404	M	20 weeks	Non-int. immunized	Not used
THX	405	M	20 weeks	Non-int. immunized	Not used
THX	406	F	20 weeks	NP-CGG	Fig. 4a–f, Fig. 5a and Ext. Data Fig. 4a,b
THX	407	F	20 weeks	NP-CGG	Fig. 4a–f, Fig. 5a and Ext. Data Fig. 4a,b
THX	408	M	20 weeks	NP-CGG	Fig. 4a–f, Fig. 5a and Ext. Data Fig. 4a,b
THX	409	M	20 weeks	NP-CGG	Fig. 4a,d–f and Ext. Data Fig. 4a,b
THX	410	F	20 weeks	NP-CGG	Fig. 4a
THX	411	M	20 weeks	NP-CGG	Fig. 4a
THX	412	M	24 weeks	NP-CGG	Fig. 4a
THX	413	F	24 weeks	NP-CGG	Fig. 5b–d,f
THX	414	F	24 weeks	NP-CGG	Fig. 5b–d,f
THX	415	M	24 weeks	NP-CGG	Fig. 5b–d,f
THX	416	F	20 weeks	NP-CGG	Fig. 5b
THX	417	M	20 weeks	NP-CGG	Fig. 5b
THX	418	M	20 weeks	NP-CGG	Fig. 5g,h
THX	419	M	20 weeks	NP-CGG	Fig. 5g,h
THX	420	M	20 weeks	NP-CGG	Fig. 5g,h–k
THX	421	F	20 weeks	NP-CGG	Fig. 5g,h–k
THX	422	F	20 weeks	NP-CGG	Fig. 5g,h–k
THX	423	F	20 weeks	NP-CGG	Fig. 5g,h
THX	424	M	20 weeks	DNP-CpG	Fig. 4g,h and Ext. Data Fig. 5a,b
THX	425	F	20 weeks	DNP-CpG	Fig. 4g,h and Ext. Data Fig. 5a,b
THX	426	F	20 weeks	DNP-CpG	Fig. 4g,h and Ext. Data Fig. 5a,b
THX	427	M	24 weeks	DNP-CpG	Fig. 4g
THX	428	F	24 weeks	DNP-CpG	Fig. 4g
THX	429	M	24 weeks	DNP-CpG	Fig. 4g,
THX	430	M	24 weeks	DNP-CpG	Fig. 4g,j and Ext. Data Fig. 2b
THX	431	F	20 weeks	DNP-CpG	Fig. 4i–p and Ext. Data Fig. 2b
THX	432	M	20 weeks	DNP-CpG	Fig. 4i–p and Ext. Data Fig. 2b
THX	433	F	20 weeks	DNP-CpG	Fig. 4i–p and Ext. Data Fig. 2b
THX	434	F	20 weeks	DNP-CpG	Fig. 4i–p and Ext. Data Fig. 2b
THX	435	M	20 weeks	DNP-CpG	Fig. 4i–p and Ext. Data Fig. 2b
THX	436	F	20 weeks	Non-int. immunized	Ext. Data Fig. 5b
THX	437	F	20 weeks	Non-int. immunized	Ext. Data Fig. 5b
THX	438	M	20 weeks	Non-int. immunized	Ext. Data Fig. 5b
THX	439	M	20 weeks	NP-CGG	Fig. 5e

THX	440	F	20 weeks	NP-CGG	Fig. 5e
THX	441	M	20 weeks	NP-CGG	Fig. 5e
THX	442	F	20 weeks	Non-int. immunized	Ext. Data Fig. 6
THX	443	M	20 weeks	Non-int. immunized	Ext. Data Fig. 6
THX	444	F	20 weeks	Non-int. immunized	Ext. Data Fig. 6
THX	445	F	22 weeks	Non-vaccinated	Fig. 6a–c
THX	446	F	22 weeks	Non-vaccinated	Fig. 6a–c
THX	447	F	22 weeks	Non-vaccinated	Fig. 6a,c
THX	448	M	22 weeks	Non-vaccinated	Fig. 6a,c
THX	449	M	22 weeks	Non-vaccinated	Fig. 6a,c
THX	450	M	22 weeks	Flagellin	Fig. 6a–c, e,g–i, Ext. Data Fig. 7b–e, Ext. Data Fig. 8
THX	451	M	22 weeks	Flagellin	Fig. 6a–c,g,h, Ext. Data Fig. 7a–e and Ext. Data Fig. 8
THX	452	F	22 weeks	Flagellin	Fig. 6a,c,g,h, Ext. Data Fig. 7a–d and Ext. Data Fig. 8
THX	453	F	22 weeks	Flagellin	Fig. 6a,c, Ext. Data Fig. 7a,c and Ext. Data Fig. 8
THX	454	M	22 weeks	Flagellin	Fig. 6a,c and Ext. Data Fig. 8
THX	455	M	22 weeks	Non-vaccinated	Fig. 6d
THX	456	M	22 weeks	Non-vaccinated	Fig. 6d
THX	457	F	22 weeks	Non-vaccinated	Fig. 6d
THX	458	F	22 weeks	Non-vaccinated	Fig. 6d
THX	459	F	22 weeks	Non-vaccinated	Fig. 6d
THX	460	M	22 weeks	Flagellin	Fig. 6d and Ext. Data Fig. 8
THX	461	M	22 weeks	Flagellin	Fig. 6d and Ext. Data Fig. 8
THX	462	F	22 weeks	Flagellin	Fig. 6d and Ext. Data Fig. 8
THX	463	F	22 weeks	Flagellin	Fig. 6d and Ext. Data Fig. 8
THX	464	F	22 weeks	Flagellin	Fig. 6d and Ext. Data Fig. 8
THX	465	M	22 weeks	Non-vaccinated	Fig. 7a
THX	466	M	22 weeks	Non-vaccinated	Fig. 7a
THX	467	M	22 weeks	Non-vaccinated	Fig. 7a
THX	468	M	22 weeks	Non-vaccinated	Fig. 7a
THX	469	F	22 weeks	Non-vaccinated	Fig. 7a
THX	470	F	22 weeks	Non-vaccinated	Fig. 7a
THX	471	F	22 weeks	Non-vaccinated	Fig. 7a
THX	472	F	22 weeks	Non-vaccinated	Fig. 7a
THX	473	M	22 weeks	Pfizer mRNA	Fig. 7a–c, Ext. Data Fig. 8 and Ext. Data Fig. 9a
THX	474	F	22 weeks	Pfizer mRNA	Fig. 7a–d, Ext. Data Fig. 8 and Ext. Data Fig. 9a
THX	475	M	22 weeks	Pfizer mRNA	Fig. 7a–d, Ext. Data Fig. 8 and Ext. Data Fig. 9a
THX	476	F	22 weeks	Pfizer mRNA	Fig. 7a and Ext. Data Fig. 8
THX	477	M	22 weeks	Pfizer mRNA	Fig. 7a,d–g, Ext. Data Fig. 8 and Ext. Data Fig. 9b
THX	478	F	22 weeks	Pfizer mRNA	Fig. 7a,d–g, Ext. Data Fig. 8 and Ext. Data Fig. 9b
THX	479	M	22 weeks	Pfizer mRNA	Fig. 7a,e–g, Ext. Data Fig. 8 and Ext. Data Fig. 9b
THX	480	F	22 weeks	Pfizer mRNA	Fig. 7a and Ext. Data Fig. 8
THX	481	F	22 weeks	Non-vaccinated	Ext. Data Fig. 10a,b
THX	482	F	22 weeks	Non-vaccinated	Ext. Data Fig. 10a,b
THX	483	M	22 weeks	Non-vaccinated	Ext. Data Fig. 10a,b
THX	484	F	22 weeks	Non-vaccinated	Ext. Data Fig. 10a,b
THX	485	M	22 weeks	Non-vaccinated	Ext. Data Fig. 10a,b
THX	486	M	22 weeks	Non-vaccinated	Ext. Data Fig. 10a,b

THX	487	F	22 weeks	RBD-KLH	Ext. Data Fig. 10a,b
THX	488	F	22 weeks	RBD-KLH	Ext. Data Fig. 10a,b–f
THX	489	M	22 weeks	RBD-KLH	Ext. Data Fig. 10a,b
THX	490	M	22 weeks	RBD-KLH	Ext. Data Fig. 10a,b–f
THX	491	M	22 weeks	RBD-KLH	Ext. Data Fig. 10a,b
THX	492	F	22 weeks	RBD-KLH	Ext. Data Fig. 10a,b–f
THX	493	M	22 weeks	Pfizer mRNA	Ext. Data Fig. 8
THX	494	M	22 weeks	Pfizer mRNA	Ext. Data Fig. 8
THX	495	F	22 weeks	Pfizer mRNA	Ext. Data Fig. 8
THX	496	F	22 weeks	Pfizer mRNA	Ext. Data Fig. 8
THX	497	F	22 weeks	Pfizer mRNA	Ext. Data Fig. 8
THX	498	F	22 weeks	Pfizer mRNA	Ext. Data Fig. 8
THX	499	F	18 weeks	Non-int. immunized	Fig. 8a,b
THX	500	M	18 weeks	Non-int. immunized	Fig. 8a,b
THX	501	F	18 weeks	Non-int. immunized	Fig. 8a,b
THX	502	F	18 weeks	Non-int. immunized	Fig. 8a,b
THX	503	F	18 weeks	Non-int. immunized	Fig. 8a,b
THX	504	M	18 weeks	Non-int. immunized	Fig. 8a,b
THX	505	F	18 weeks	Non-int. immunized	Fig. 8a,b
THX	506	M	18 weeks	Non-int. immunized	Fig. 8a
THX	507	M	18 weeks	Non-int. immunized	Fig. 8a
THX	508	F	18 weeks	Non-int. immunized	Fig. 8a
THX	509	M	18 weeks	Non-int. immunized	Fig. 8a
THX	510	M	18 weeks	Non-int. immunized	Fig. 8a
THX	511	F	18 weeks	Non-int. immunized	Fig. 8c,e
THX	512	M	18 weeks	Non-int. immunized	Fig. 8c,e
THX	513	F	18 weeks	Non-int. immunized	Fig. 8c,e
THX	514	M	20 weeks	Non-int. immunized	Supplementary Table 5a,b
THX	515	F	20 weeks	Non-int. immunized	Supplementary Table 5a,b
THX	516	M	20 weeks	Non-int. immunized	Supplementary Table 5a,b
THX	517	F	20 weeks	Non-int. immunized	Supplementary Table 5a,b
THX	518	F	20 weeks	Non-int. immunized	Supplementary Table 5a,b
THX	519	F	20 weeks	Non-int. immunized	Supplementary Table 5a,b
THX	520	F	20 weeks	Non-int. immunized	Supplementary Table 5a,b
THX	521	M	20 weeks	Non-int. immunized	Supplementary Table 5a,b
THX	522	M	20 weeks	Non-int. immunized	Supplementary Table 5a,b
THX	523	M	20 weeks	Non-int. immunized	Supplementary Table 5a,b
THX	524	M	20 weeks	Non-int. immunized	Supplementary Table 5a,b
THX	525	F	20 weeks	Non-int. immunized	Supplementary Table 5a,b
THX	526	F	20 weeks	Non-int. immunized	Supplementary Table 5a,b
THX	527	F	20 weeks	Non-int. immunized	Supplementary Table 5a,b
THX	528	F	20 weeks	Non-int. immunized	Supplementary Table 5a,b
THX	529	M	22 weeks	Flagellin	Ext. Data Fig. 8 and Supplementary Table 6
THX	530	F	22 weeks	Flagellin	Ext. Data Fig. 8 and Supplementary Table 6
THX	531	F	22 weeks	Flagellin	Ext. Data Fig. 8 and Supplementary Table 6
THX	532	F	22 weeks	Flagellin	Ext. Data Fig. 8 and Supplementary Table 6
THX	533	M	22 weeks	Flagellin	Ext. Data Fig. 8 and Supplementary Table 6

THX	534	M	22 weeks	Flagellin	Ext. Data Fig. 8 and Supplementary Table 6
THX	535	F	22 weeks	Pfizer mRNA	Ext. Data Fig. 8 and Supplementary Table 7
THX	536	F	22 weeks	Pfizer mRNA	Ext. Data Fig. 8 and Supplementary Table 7
THX	537	F	22 weeks	Pfizer mRNA	Ext. Data Fig. 8 and Supplementary Table 7
THX	538	F	22 weeks	Pfizer mRNA	Ext. Data Fig. 8 and Supplementary Table 7
THX	539	M	22 weeks	Pfizer mRNA	Ext. Data Fig. 8 and Supplementary Table 7
THX	540	M	22 weeks	Pfizer mRNA	Ext. Data Fig. 8 and Supplementary Table 7
THX	541	M	22 weeks	Pfizer mRNA	Ext. Data Fig. 8 and Supplementary Table 7
THX	542	F	22 weeks	Pfizer mRNA	Ext. Data Fig. 8 and Supplementary Table 7
THX	543	M	18 weeks	Non-int. immunized	Fig. 1a
THX	544	F	18 weeks	Non-int. immunized	Fig. 1a
THX	545	F	18 weeks	Non-int. immunized	Fig. 1a
THX	546	F	18 weeks	Non-int. immunized	Fig. 1a
THX	547	F	18 weeks	Non-int. immunized	Fig. 1a
THX	548	F	18 weeks	Non-int. immunized	Fig. 1a
THX	549	M	20 weeks	Non-int. immunized	Ext. Data Fig. 2a
THX	550	M	20 weeks	Non-int. immunized	Ext. Data Fig. 2a
THX	551	M	20 weeks	Non-int. immunized	Ext. Data Fig. 2a
THX	552	M	20 weeks	Non-int. immunized	Ext. Data Fig. 2a
THX	553	F	20 weeks	Non-int. immunized	Ext. Data Fig. 2a
THX	554	F	20 weeks	Non-int. immunized	Ext. Data Fig. 2a
THX	555	F	20 weeks	Non-int. immunized	Ext. Data Fig. 2a
THX	556	M	22 weeks	Flagellin	Fig. 6f
THX	557	M	22 weeks	Flagellin	Fig. 6f
THX	558	F	22 weeks	Flagellin	Fig. 6f
THX	559	F	22 weeks	Flagellin	Fig. 6f

THX mice were constructed by feeding huNBSGW mice with 17β-estradiol (1.5 μM in drinking water) *ad libitum* starting at 14–18 weeks of age and continuing thereafter. After 4 weeks, E2-conditioned huNBSGW mice (referred to as THX mice) were ready for experimental use or continued on E2 for use at a later time.

Supplementary Table 13 | Antibodies used in fluorescence flow cytometry and microscopy.

Species	Antibody	Vendor	Catalog number
Mouse	anti–huCD45-APC mAb	BioLegend	Cat. # 304011 (Clone HI30)
Mouse	anti–huCD45-FITC mAb	BioLegend	Cat. # 368507 (Clone 30-F11)
Mouse	anti-huCD45-PE mAb	BioLegend	Cat. # 368509 (Clone 2D1)
Rat	anti–moCD45-Pacific Blue™ mAb	BioLegend	Cat. # 103125 (Clone 2D1)
Mouse	anti-huIgM-PE mAb	BioLegend	Cat. # 314507 (Clone MHM-88)
Mouse	anti–huIgM-BV510™ mAb*	BioLegend	Cat. # 314521 (Clone MHM-88)
Mouse	anti-huIgM-BV650™ mAb*	BioLegend	Cat. # 314525 (Clone MHM-88)
Mouse	anti–huIgM-APC-Fire™ 750 mAb	BioLegend	Cat. # 314545 (Clone MHM-88)
Mouse	anti–huIgD-BV421™mAb*	BioLegend	Cat. # 348225 (Clone IA6-2)
Mouse	anti–huIgD-BV785™ mAb	BioLegend	Cat. # 348241 (Clone IA6-2)
Mouse	anti–huIgD-FITC mAb	BioLegend	Cat. # 348205 (Clone IA6-2)
Rat	anti-huIgG-BV421 mAb	BioLegend	Cat. # 410703 (Clone M1310G05)
Mouse	anti–huIgG-FITC mAb*	BD Pharmingen	Cat. # 555786 (Clone G18-145)
Goat	anti-huIgA-FITC Ab	Invitrogen	Cat. # 31577
Mouse	anti–huIgA-APC mAb*	Miltenyi Biotec	Cat. # 130-113-472 (Clone IS11-8E10)
Mouse	anti–huIgE-APC-Fire™ mAb	BioLegend	Cat. # 325515 (Clone MHE-18)
Mouse	anti–huCD27-PE mAb	BioLegend	Cat. # 356405 (Clone M-T271)
Mouse	anti–huCD27-APC-Cyanine7 mAb	TONBO	Cat. # 25-0279-T100 (Clone O323)
Mouse	anti-huCD19-PE mAb	BioLegend	Cat. # 302208 (Clone HIB19)
Mouse	anti–huCD19-PE-Cyanine7 mAb*	BioLegend	Cat. # 302216 (Clone HIB19)
Mouse	anti-huCD20-FITC mAb	BioLegend	Cat. # 302303 (Clone 2H7)
Mouse	anti–huCD138-BV510 mAb	BioLegend	Cat. # 356517 (Clone MI15)
Mouse	anti–huCD38-BV650 mAb	BioLegend	Cat. # 356619 (Clone HB-7)
Mouse	anti–huCD11c- APC-Cyanine7 mAb	BioLegend	Cat. # 337217 (Clone Bu15)
Mouse	anti–huCD14-APC mAb	BioLegend	Cat. # 367117 (Clone 63D3)
Mouse	anti–huCD56-BV786 mAb	BioLegend	Cat. # 362549 (Clone 5.1H11)
Mouse	anti–huCD5-PE-Cyanine7 mAb	BioLegend	Cat. # 300621 (Clone UCHT2)
Mouse	anti–huCD3-Super Bright 600 mAb*	eBioscience	Cat. # 63003741 (Clone OKT3)
Rat	anti–huCD4-APC mAb	BioLegend	Cat. # 357407 (Clone A161A1)
Rat	anti-huCD4-BV421™mAb*	BioLegend	Cat. # 357423 (Clone A161A1)
Mouse	anti–huCD8-PE mAb	BioLegend	Cat. # 344705 (Clone SK1)
Mouse	anti–huCD8-Alexa Fluor® 700 mAb	BioLegend	Cat. # 344723 (Clone SK1)
Mouse	anti–huCXCR5-PE mAb	BioLegend	Cat. # 356903 (Clone J252D4)
Mouse	anti–huCXCR5-FITC mAb	BioLegend	Cat. # 356913 (Clone J252D4)
Hamster	anti–huICOS-Pacific Blue™ mAb	BioLegend	Cat. # 313521 (Clone C398.4A)
Mouse	anti–huPD-1-FITC mAb	BioLegend	Cat. # 367411 (Clone NAT105)
Mouse	anti–huPD-1-PE-Cyanine7 mAb	BioLegend	Cat. # 621615 (Clone A17188B)
Mouse	anti–huHLA-A,B,C (MHC I)-APC mAb	BioLegend	Cat. # 311409 (Clone W6/32)
Mouse	anti–huHLA-DR, DP, DQ (MHC II)-FITC mAb	BioLegend	Cat. # 361705 (Clone Tü39)
Rat	anti–moEpCAM-PE-Cyanine7 mAb	BioLegend	Cat. # 118216 (Clone G8.8)

Rabbit	anti–huEpCAM-PE mAb	Abcam	Cat. # ab237397 (Clone EPR20532-225)
Rat	anti–moTER-119-APC mAb	BioLegend	Cat. # 116211 (Clone TER-119)
Mouse	anti–huCD235a-FITC mAb	BioLegend	Cat. # 349103 (Clone HI264)
Rat	anti–moCD41-PE-Cyanine7 mAb	BioLegend	Cat. # 133915 (Clone MWReg30)
Mouse	anti–huCD61-PerCp mAb	BioLegend	Cat. # 336409 (Clone VI-PL2)
Rabbit	anti–huAID-Alexa Fluor® 647 Ab	Bioss	Cat. # bs-7855R-FITC
Mouse	anti–huBLIMP1-Alexa Fluor® 488 mAb	R&D Systems	Cat. # IC36081G (Clone 646702)

*Antibodies used in both fluorescence flow cytometry and fluorescence microscopy.

Supplementary Table 14 | Healthy adult human subjects source of PBMCs, B cells, T cells and sera.

Donor		Sex	Age range	Experiments
1	HS 01	F	61–65	Naïve IgM^+IgD^+ B cells used in Fig. 2a–f
2	HS 02	M	36–40	Naïve IgM^+IgD^+ B cells used in Fig. 2a–f
3	HS 03	M	20–25	Naïve IgM^+IgD^+ B cells used in Fig. 2a–f
4	HS 04	M	26–30	Naïve IgM^+IgD^+ B cells not used
5	HS 05	F	36–40	T cells used in Fig. 3a–e
6	HS 06	F	20–25	T cells used in Fig. 3a–e
7	HS 07	M	36–40	T cells used in Fig. 3a–e
8	HS 08	F	20–25	Sera used in Fig. 6c
9	HS 09	F	36–40	Sera used in Fig. 6c
10	HS 10	M	26–30	Sera used in Fig. 6c
11	HS 11	F	36–40	Sera used in Fig. 6c
12	HS 12	M	31–35	Sera used in Fig. 6c
13	HS 13	F	31–35	PBMCs used in Fig. 6e
14	HS 14	F	41–45	Naïve IgM^+IgD^+ B cells used in Extended Data Fig. 6a,d
15	HS 15	M	51–55	Naïve IgM^+IgD^+ B cells used in Extended Data Fig. 6b,d
16	HS 16	F	31–35	Naïve IgM^+IgD^+ B cells used in Extended Data Fig. 6c,d
17	HS 17	M	36–40	PBMCs used in Ext. Data Fig. 7a

HuPBMCs, naïve $huIgM^+IgD^+$ B cells, huT cells and sera were acquired from healthy male and female human subjects of different ages, races and ethnic backgrounds.

Supplementary Table 15 | Antibodies used in ELISAs, ELISPOTs and cell isolation

Species	Antibody	Vendor	Catalog number
Goat	anti–huIgM Ab	SouthernBiotech	Cat. # 2020-01
Goat	anti–huIgG Ab	SouthernBiotech	Cat. # 2015-01
Goat	anti-huIgD Ab	SouthernBiotech	Cat. # 2030-01
Goat	anti–huIgA Ab	SouthernBiotech	Cat. # 2050-01
Goat	anti–huIgE Ab	ICL Labs	Cat. # GE-80A
Goat	anti–huIgM Ab-biotin	SouthernBiotech	Cat. # 2020-08
Goat	anti-huIgD Ab-biotin	SouthernBiotech	Cat. # 2030-08
Goat	anti–huIgG Ab-biotin	SouthernBiotech	Cat. # 2015-08
Mouse	anti–huIgG1 mAb-biotin (Clone G17-1)	BD Pharmingen	Cat. # 555869
Mouse	anti–huIgG2 mAb-biotin (Clone G18-2)	BD Pharmingen	Cat. # 555874
Mouse	anti–huIgG3 mAb-biotin (Clone MTG34)	MABTECH	Cat. # 3853-6-250
Mouse	anti–huIgG4 mAb-biotin (Clone G17-4)	BD Pharmingen	Cat. # 555882
Goat	anti–huIgA Ab-biotin	SouthernBiotech	Cat. # 2050-08
Mouse	anti–huIgE mAb-biotin (Clone HP6029)	SouthernBiotech	Cat. # 9250-08
Mouse	anti–huCD43 mAb-biotin (Clone DF-T1)	SouthernBiotech	Cat. # 9620-08
Mouse	anti–huCD3 mAb-biotin (Clone UCHT1)	BioLegend	Cat. # 300403
Mouse	anti-huIgD mAb-biotin (Clone IA6-2)	BioLegend	Cat. # 348212

mAb, monoclonal antibody

Supplementary Table 16 | PCR primers used in RT-PCR, qRT-PCR and RT-5'RACE PCR

	Forward Primer	Reverse Primer
qRT-PCR: Human genes		
AICDA	5'-GTCACCTGGTTCACCTCCTG-3'	5'-CTTGCGGTCCTCACAGAAGT-3'
PRDM1	5'-ATCTTGGGGTAAAAGCGGGT-3'	5'-TCCTGCACTACTGGACACAC-3'
β-ACTIN	5'-AGAGCTACGAGCTGCCTGAC-3'	5'-AGCACTGTGTTGGCGTACAG-3'
HPRT1	5'-TGCTCGAGATGTGATGAAGG-3'	5'-CCCCTGTTGACTGGTCATT-3'
qRT-PCR: Human mature transcripts		
V_HDJ_H-Cμ	5'-GACACGGCYGTRTATTACTGTGCG-3'	5'-CTTTCGCTCCAGGTCACACT-3'
V_HDJ_H-Cγ	5'-GACACGGCYGTRTATTACTGTGCG-3'	5′-CGGGGAAGTAGTCCTTGACC-3′
V_HDJ_H-Cα	5'-GACACGGCYGTRTATTACTGTGCG-3'	5'-GTGGGAAGTTTCTGGCGGT-3'
V_HDJ_H-Cε	5'-GACACGGCYGTRTATTACTGTGCG-3'	5'-CGGAGGTGGCATTGGAGG-3'
Somatic mutations and VDJ repertoires		
RT-PCR: V_H family specific		
V1DJ_H-Cμ		
First round	5'-TCHTCACCATGGACTGGACCTGGAG-3'	5'-GTGATGGAGTCGGGAAGGAAG-3'
Second round	5'-TCGTCGGCAGCGTCAGATGTGTATA<br>AGAGACAGTCHTCACCATGGACTGGACCT<br>GGAG-3'	5'-GTCTCGTGGGCTCGGAGATGTGTATA<br>AGAGACAGGGGTGATGGAGTCGGGAAG<br>GAAG-3'
V3DJ_H-Cμ		
First round	5'-CACCATGGAGTTTGGGCTGAGCT-3'	5'-GTGATGGAGTCGGGAAGGAAG-3'
Second round	5'-TCGTCGGCAGCGTCAGATGTGTATAAG<br>AGACAG]CACCATGGAGTTTGGGCTGAGCT-3'	5'-GTCTCGTGGGCTCGGAGATGTGTATA<br>AGAGACAGGGGTGATGGAGTCGGGAAG<br>GAAG-3'
V1DJ_H-Cγ		
First round	5'-TCHTCACCATGGACTGGACCTGGAG-3'	5'-GAAGTAGTCCTTGACCAGGCA-3'
Second round	5'-TCGTCGGCAGCGTCAGATGTGTATA<br>AGAGACAGTCHTCACCATGGACTGGACCT<br>GGAG-3'	5'-GTCTCGTGGGCTCGGAGATGTGTATA<br>AGAGACAGGGGAAGTAGTCCTTGACCA-3'
V3DJ_H-Cγ		
First round	5'-CACCATGGAGTTTGGGCTGAGCT-3'	5'-GAAGTAGTCCTTGACCAGGCA-3'
Second round	5'-TCGTCGGCAGCGTCAGATGTGTATAAG<br>AGACAG]CACCATGGAGTTTGGGCTGAGCT-3'	5'-GTCTCGTGGGCTCGGAGATGTGTATA<br>AGAGACAGGGGAAGTAGTCCTTGACCA-3'
V1DJ_H-Cα		
First round	5'-TCHTCACCATGGACTGGACCTGGAG-3'	5'-GCGACGACCACGTTCCCATCT-3'
Second round	5'-TCGTCGGCAGCGTCAGATGTGTATAAGAGACA<br>GTCHTCACCATGGACTGGACCTGGAG-3'	5'-GTCTCGTGGGCTCGGAGATGTGTATA<br>AGAGACAGGGGGAAGAAGCCCTGGAC-3'
V3DJ_H-Cα		
First round	5'-CACCATGGAGTTTGGGCTGAGCT-3'	5'-GCGACGACCACGTTCCCATCT-3'
Second round	5'TCGTCGGCAGCGTCAGATGTGTATAAGA<br>GACAG]CACCATGGAGTTTGGGCTGAGCT-3'	5'-GTCTCGTGGGCTCGGAGATGTGTATA<br>AGAGACAGGGGGAAGAAGCCCTGGAC-3'

RT-5' RACE PCR: All V_H families		
V_HDJ_H-Cμ		
RT		5'-GTGATGGAGTCGGGAAGGAAG-3'
First round	5'CCAGTGAGCAGAGTGACGAGGACTCGAGCTC AAGCTTTTTTTTTTTTTTTTT-3'	5'-GTGATGGAGTCGGGAAGGAAG-3'
Second round	5'-CCAGTGAGCAGAGTGACG-3'	5'-GTCTCGTGGGCTCGGAGATGTGTATA AGAGACAGGGGTGATGGAGTCGGGAAG GAAG-3'
V_HDJ_H-Cγ		
RT		5'-GAAGTAGTCCTTGACCAGGCA-3'
First round	5'CCAGTGAGCAGAGTGACGAGGACTCGAGCTCAAG CTTTTTTTTTTTTTTTTT-3'	5'-GAAGTAGTCCTTGACCAGGCA-3'
Second round	5'-CCAGTGAGCAGAGTGACG-3'	5'-GTCTCGTGGGCTCGGAGATGTGTATA AGAGACAGGGGAAGTAGTCCTTGACCA-3'
V_HDJ_H-Cα		
RT		5'-GTGGGAAGTTTCTGGCGGT-3'
First round	5'CCAGTGAGCAGAGTGACGAGGACTCGAGCTCAAG CTTTTTTTTTTTTTTTTT-3'	5'-GCGACGACCACGTTCCCATCT-3'
Second round	5'-CCAGTGAGCAGAGTGACG-3'	5'-GTCTCGTGGGCTCGGAGATGTGTATAA GAGACAGGGGGAAGAAGCCCTGGAC-3'
VκJκ-Cκ		
RT		5'-TTCGCAGGCGTAGACTTTGT-3'
PCR	5'-CCAGTGAGCAGAGTGACGAGGACTCGA GCTCAAGCTTTTTTTTTTTTTTTTT-3'	5'-GTCTCGTGGGCTCGGAGATGTGTATA AGAGACAGCTCTCCTGGGAGTTACCCGA-3'
VλJλ-Cλ		
RT		5'-ACTAATGCGTGACCTGGCAGCTGT-3'
PCR	5'-CCAGTGAGCAGAGTGACGAGGACTCGA GCTCAAGCTTTTTTTTTTTTTTTTT-3'	5'-GTCTCGTGGGCTCGGAGATGTGTATAAGAG ACAGCAGGCTCAGATAGCTGCTGG-3'
TCRα		
RT		5'-GATTTGTTGCTCCAGGCCAC-3'
PCR	5'-CCAGTGAGCAGAGTGACGAGGACTCGA GCTCAAGCTTTTTTTTTTTTTTTTT-3'	5'-GTCTCGTGGGCTCGGAGATGTGTATA AGAGACAGTCTCAGCTGGTACACGGCAG-3'
TCRβ		
RT		5'-CTCCTTCCCATTCACCCACC-3'
PCR	5'-CCAGTGAGCAGAGTGACGAGGACTCGA GCTCAAGCTTTTTTTTTTTTTTTTT-3'	5'-GTCTCGTGGGCTCGGAGATGTGTATA AGAGACAGAGATCTCTGCTTCTGATGGCTC-3'

Supplementary Table 17 | Lupus THX mice.

Lupus THX mouse		Constructed using	Sex	Age	Injection	Mouse or mouse cells used in experiments of:
Lupus THX	701	NBSGW mouse	F	18 weeks	Pristane	Fig. 8a,b
Lupus THX	702	NBSGW mouse	F	18 weeks	Pristane	Fig. 8a,b
Lupus THX	703	NBSGW mouse	M	18 weeks	Pristane	Fig. 8a–g
Lupus THX	704	NBSGW mouse	F	18 weeks	Pristane	Fig. 8a
Lupus THX	705	NBSGW mouse	M	18 weeks	Pristane	Fig. 8a
Lupus THX	706	NSGW41 mouse	M	18 weeks	Pristane	Fig. 8a,b
Lupus THX	707	NSGW41 mouse	M	18 weeks	Pristane	Fig. 8a,b
Lupus THX	708	NSGW41 mouse	F	18 weeks	Pristane	Fig. 8a–g
Lupus THX	709	NSGW41 mouse	F	18 weeks	Pristane	Fig. 8a–e
Lupus THX	710	NBSGW mouse	F	18 weeks	Pristane	Fig. 8a
Lupus THX	711	NSGW41 mouse	F	18 weeks	Pristane	Fig. 8a

Lupus THX mice were generated by injecting THX mice (18-week-old, constructed by $huCD34^+$ cell engraftment of 6 NBSGW and 5 NSGW41 mice) once i.p. with pristane (500 μl) and continuing E2 treatment. All Lupus THX mice were non-intentionally immunized.
