# Supplementary material for: A humanized mouse that mounts mature class-switched, hypermutated and neutralizing antibody responses
Source: Nat Immunol. 2024 Jun 25;25(8):1489–506. doi: 10.1038/s41590-024-01880-3 (PMC11291283; doi:10.1038/s41590-024-01880-3)
Supplement: Supplementary file 2 — Reporting Summary [file 41590_2024_1880_MOESM2_ESM.pdf]

Corresponding author(s): Paolo Casali

Last updated by author(s): May 10, 2024

Reporting Summary

Nature Portfolio wishes to improve the reproducibility of the work that we publish. This form provides structure for consistency and transparency in reporting. For further information on Nature Portfolio policies, see our Editorial Policies and the Editorial Policy Checklist.

Statistics

For all statistical analyses, confirm that the following items are present in the figure legend, table legend, main text, or Methods section.

n/a	Confirmed	
☐	☒	The exact sample size (*n*) for each experimental group/condition, given as a discrete number and unit of measurement
☐	☒	A statement on whether measurements were taken from distinct samples or whether the same sample was measured repeatedly
☐	☒	The statistical test(s) used AND whether they are one- or two-sided<br>*Only common tests should be described solely by name; describe more complex techniques in the Methods section.*
☒	☐	A description of all covariates tested
☒	☐	A description of any assumptions or corrections, such as tests of normality and adjustment for multiple comparisons
☐	☒	A full description of the statistical parameters including central tendency (e.g. means) or other basic estimates (e.g. regression coefficient) AND variation (e.g. standard deviation) or associated estimates of uncertainty (e.g. confidence intervals)
☐	☒	For null hypothesis testing, the test statistic (e.g. *F*, *t*, *r*) with confidence intervals, effect sizes, degrees of freedom and *P* value noted<br>*Give P values as exact values whenever suitable.*
☒	☐	For Bayesian analysis, information on the choice of priors and Markov chain Monte Carlo settings
☒	☐	For hierarchical and complex designs, identification of the appropriate level for tests and full reporting of outcomes
☒	☐	Estimates of effect sizes (e.g. Cohen's *d*, Pearson's *r*), indicating how they were calculated

Our web collection on statistics for biologists contains articles on many of the points above.

Software and code

Policy information about availability of computer code

Data collection	BioTek Gen5 Software v2.07 (Agilent), CTL ImmunoCapture Software v6.5.7 (Immunospot), BD FACSDiva Software v9.4 (BD Biosciences), CyTOF Software v6.7 for Maxpar Direct Immune Profiling Assay (Fluidigm), ZEN Microscopy Software v3.9 (ZEISS), Bio-Rad CFX Manager Software v3.1 (Bio-Rad)
Data analysis	GraphPad Prism v10.0.3 (GraphPad Software Inc.), Microsoft Excel v16.83, CTL ImmunoCapture Software v6.5.7 (Immunospot), FlowJo v10.9 (TreeStar), Maxpar Pathsetter software v3.0 (Fluidigm), IMGT/HighV-QUEST v1.9.2 (The International ImMunoGeneTics Information System), PHYLOViZ v2.0 (PHYLOViZ), Ribosomal Database Project Classifier v2.14 (http://rdp.cme.msu.edu/classifier). ClustVis v1.0 (https://biit.cs.ut.ee/clustvis/)

For manuscripts utilizing custom algorithms or software that are central to the research but not yet described in published literature, software must be made available to editors and reviewers. We strongly encourage code deposition in a community repository (e.g. GitHub). See the Nature Portfolio guidelines for submitting code & software for further information.

Data

Policy information about availability of data

All manuscripts must include a data availability statement. This statement should provide the following information, where applicable:

- Accession codes, unique identifiers, or web links for publicly available datasets
- A description of any restrictions on data availability
- For clinical datasets or third party data, please ensure that the statement adheres to our policy

MiSeq amplicon sequencing data has been deposited in NCBI Sequence Read Archive (SRA) under BioProject ID PRJNA 1047643. All other data supporting the findings of this study are present in the paper and/or Supplementary Information.

Research involving human participants, their data, or biological material

Policy information about studies with human participants or human data. See also policy information about sex, gender (identity/presentation), and sexual orientation and race, ethnicity and racism.

Reporting on sex and gender	Human PBMCs were isolated from buffy coats obtained from healthy male and female human donors. Human umbilical cord blood was collected from full-term, normally developed male and female newborns (in approximately equal numbers) from healthy puerperae.
Reporting on race, ethnicity, or other socially relevant groupings	Human PBMCs were isolated from buffy coats obtained from healthy male and female human donors of different ages (18- to 65-year-olds), races and ethnic backgrounds (Supplementary Table 14). Human umbilical cord blood was collected from full-term, normally developed male and female newborns from healthy puerperae (18- to 45-year-olds) of different ages, races and ethnic backgrounds (Supplementary Table 9) immediately after cesarean section.
Population characteristics	Human PBMCs were isolated from buffy coats obtained from healthy male and female human donors of different ages (18- to 65-year-olds), races and ethnic backgrounds (Supplementary Table 14). Human umbilical cord blood was collected from full-term, normally developed male and female newborns (in approximately equal numbers) from healthy puerperae (18- to 45-year-olds with no infectious disease or history of cancer) of different ages, races and ethnic backgrounds (Supplementary Table 9) immediately after cesarean section.
Recruitment	PBMCs of healthy human subjects were isolated from buffy coats obtained from the South Texas Blood and Tissue Center, San Antonio, TX (STBTC), under the Healthy Volunteer Blood Donor Program. The healthy subjects were enrolled and consented by STBTC. Human umbilical cord blood was collected from full-term, normally developed male and female newborns from healthy puerperae (18- to 45-year-olds with no infectious disease or history of cancer) recruited by the Department of Obstetrics and Gynecology, The University of Texas Long School of Medicine, The University of Texas Health Science Center at San Antonio and obtained upon informed consent, as per protocol of The University of Texas Health Science Center at San Antonio Institutional Review Board (IRB Protocol 17-653H). For collection of buffy coats and umbilical cord blood, no self-selection bias or other biases were known to be present as the investigators have no interaction with donors and have not been provided with any personal or medical history of such donors.
Ethics oversight	Peripheral blood was obtained upon informed consent from donors, as per protocol of the South Texas Blood and Tissue Center, San Antonio, TX. Human umbilical cord blood was obtained upon informed consent from puerperae, as per protocol of The University of Texas Health Science Center at San Antonio Institutional Review Board (IRB Protocol 17-653H).

Note that full information on the approval of the study protocol must also be provided in the manuscript.

Field-specific reporting

Please select the one below that is the best fit for your research. If you are not sure, read the appropriate sections before making your selection.

☒ Life sciences ☐ Behavioural & social sciences ☐ Ecological, evolutionary & environmental sciences

For a reference copy of the document with all sections, see nature.com/documents/nr-reporting-summary-flat.pdf

Life sciences study design

All studies must disclose on these points even when the disclosure is negative.

Sample size	The exact sample size of all in vivo and in vitro experiments is reported in Figure Legends. In each in vivo experiment, at least 5 mice per group (with the exception of the experiment of Fig. 1g in which data were from 3 mice) were used to ensure proper biological replicates. Sample size calculations for in vivo and in vitro experiments were performed using power analysis, which accounts for effect size, standard deviation, type 1 error and 80% power in a two-sample t-test with a 5% significance level (two-sided test). G power software version 3.1.9.7 was used for these calculations. To construct humanized mice, immunodeficient mice from one litter were grafted with huCD34+ cells from the same donor. In those cases in which litter sizes were small, multiple litters were combined and grafted with the same donor huCD34+ cells, and pups cross-fostered by a single nursing mother.
Data exclusions	Generally, THX and huNBSGW mice used in all experiments displayed up to 96.1% and 89.3% human CD45+ cells, respectively, in circulating blood. Generally, 2-3% of the constructed THX and huNBSGW mice at age 20-24 weeks displayed less than 90% and 88% human CD45+ cells

in circulating blood and excluded from study. 60% of huNSG and JAX NSG huCD34 mice displayed at peak approximately 45% and 20% human CD45+ cells, respectively, in circulating blood. huNSG and JAX NSG huCD34 mice displaying lower proportions of peak circulating blood human CD45+ cells were excluded from study. No data were excluded from analysis.

Replication	All experimental findings were reproduced with at least 3 biologically independent replicates for all experiments.
Randomization	After matching for sex and age, THX, huNBSGW, huNSG and JAX NSG huCD34 mice were randomly assigned to appropriate groups for both in vivo and in vitro experiments.
Blinding	For in vivo and in vitro experiments, investigators were not blinded to experimental group allocations because the same investigators performed sample collection, data collection and analyses. However, all experiments were performed using age and sex-matched littermates. The analysis in this study is quantitative and not qualitative in nature.

Reporting for specific materials, systems and methods

We require information from authors about some types of materials, experimental systems and methods used in many studies. Here, indicate whether each material, system or method listed is relevant to your study. If you are not sure if a list item applies to your research, read the appropriate section before selecting a response.

Materials & experimental systems

n/a	Involved in the study	
☐	☒	Antibodies
☐	☒	Eukaryotic cell lines
☒	☐	Palaeontology and archaeology
☐	☒	Animals and other organisms
☒	☐	Clinical data
☒	☐	Dual use research of concern
☒	☐	Plants

Methods

n/a	Involved in the study	
☒	☐	ChIP-seq
☐	☒	Flow cytometry
☒	☐	MRI-based neuroimaging

Antibodies

Antibodies used

For ELISA, ELISPOT and/or cell isolation:
anti–huIgM Ab (SouthernBiotech,Cat. # 2020-01)
anti–huIgG Ab (SouthernBiotech, Cat. # 2015-01)
anti-huIgD Ab (SouthernBiotech, Cat. # 2030-01)
anti–huIgA Ab (SouthernBiotech, Cat. # 2050-01)
anti–huIgE Ab (ICL Labs, Cat. # GE-80A)
anti–huIgM Ab-biotin (SouthernBiotech, Cat. # 2020-08)
anti-huIgD Ab-biotin (SouthernBiotech, Cat. # 2030-08)
anti–huIgG Ab-biotin (SouthernBiotech, Cat. # 2015-08)
anti–huIgG1 mAb-biotin (BD Pharmingen, Cat. # 555869, Clone G17-1)
anti–huIgG2 mAb-biotin (BD Pharmingen, Cat. # 555874, Clone G18-21)
anti–huIgG3 mAb-biotin (MABTECH, Cat. # 3853-6-250, Clone MTG34)
anti–huIgG4 mAb-biotin (BD Pharmingen, Cat. # 555882, Clone G17-4)
anti–huIgA Ab-biotin (SouthernBiotech, Cat. # 2050-08)
anti–huIgE mAb-biotin (SouthernBiotech, Cat. # 9250-08, Clone HP6029)
anti–huCD43 mAb-biotin (SouthernBiotech, Cat. # 9620-08, Clone DF-T1)
anti–huCD3 mAb-biotin (BioLegend, Cat. # 300403, Clone UCHT1)
anti-huIgD mAb-biotin (BioLegend, Cat. # 348212, Clone IA6-2)

For flow cytometry and/or fluorescence microscopy:
anti-huCD45-APC mAb (BioLegend, Cat. # 304011, Clone HI30, 1:100)
anti–huCD45-FITC mAb (BioLegend, Cat. # 368507, Clone 30-F11, 1:100)
anti–huCD45-PE mAb (BioLegend, Cat. # 368509, Clone 2D1, 1:100)
anti–moCD45-Pacific Blue™ mAb (BioLegend, Cat. # 103125, Clone 2D1, 1:1000)
anti–huIgM-PE mAb (BioLegend, Cat #314507, Clone MHM-88, 1:100)
anti–huIgM-BV510™ mAb (BioLegend, Cat. # 314521, Clone MHM-88, 1:100)
anti–huIgM-BV650™ mAb (BioLegend, Cat. # 314525, Clone MHM-88, 1:100)
anti–huIgM-APC-Fire™ 750 mAb (BioLegend, Cat. # 314545, Clone MHM-88, 1:100)
anti–huIgD-BV421™mAb (BioLegend, Cat. # 348225, Clone IA6-2, 1:100)
anti–huIgD-BV785™ mAb (BioLegend, Cat. # 348241, Clone IA6-2, 1:100)
anti–huIgD-FITC mAb (BioLegend, Cat. # 348205, Clone IA6-2, 1:100)
anti–huIgG-BV421 mAb (BD BioLegend, Cat. # 410703, Clone M1310G05, 1:100)
anti–huIgG-FITC mAb (BD Pharmingen, Cat. # 555786, Clone G18-145, 1:100)
anti–huIgA-FITC Ab (Invitrogen, Cat. # 31577, 1:100)
anti–huIgA-APC mAb (Miltenyi Biotec, Cat. # 130-113-472, Clone IS11-8E10, 1:100)
anti–huIgE-APC-Fire™ mAb (BioLegend, Cat. # 325515, Clone MHE-18, 1:100)
anti–huCD27-PE mAb (BioLegend, Cat. # 356405, Clone M-T271, 1:100)
anti–huCD27-APC-Cyanine7 mAb (TONBO, Cat. # 25-0279-T100, Clone O323, 1:100)

anti–huCD19-PE mAb (BioLegend, Cat. # 302208, Clone HIB19, 1:100)
anti–huCD19-PE-Cyanine7 mAb (BioLegend, Cat. # 302216, Clone HIB19, 1:100)
anti–huCD20-FITC mAb (BioLegend, Cat. # 302303, Clone 2H7, 1:100)
anti–huCD138-BV510 mAb (BioLegend, Cat. # 356517, Clone MI15, 1:100)
anti–huCD38-BV650 mAb (BioLegend, Cat. # 356619, Clone HB-7, 1:100)
anti–huCD11c- APC-Cyanine7 mAb (BioLegend, Cat. # 337217, Clone Bu15, 1:100)
anti–huCD14-APC mAb (BioLegend, Cat. # 367117, Clone 63D3, 1:100)
anti–huCD56-BV786 mAb (BioLegend, Cat. # 362549, Clone 5.1H11, 1:100)
anti–huCD5-PE-Cyanine7 mAb (BioLegend, Cat. # 300621, Clone UCHT2, 1:100)
anti–huCD3-Super Bright 600 mAb (eBioscience, Cat. # 63003741, Clone OKT3, 1:100)
anti–huCD4-APC mAb (BioLegend, Cat. # 357407, Clone A161A1, 1:100)
anti–huCD4-BV421™ mAb (BioLegend, Cat. # 357423, Clone A161A1, 1:100)
anti–huCD8-PE mAb (BioLegend, Cat. # 344705, Clone SK1, 1:100)
anti–huCD8-Alexa Fluor 700 mAb (BioLegend, Cat. # 344723, Clone SK1, 1:100
anti–huCXCR5-PE mAb (BioLegend, Cat. # 356903, Clone J252D4, 1:100)
anti–huCXCR5-FITC mAb (BioLegend, Cat. # 356913, Clone J252D4, 1:100)
anti–huICOS-Pacific Blue™ mAb (BioLegend, Cat. # 313521, Clone C398.4A, 1:100)
anti–huPD-1-FITC mAb (BioLegend, Cat. # 367411, Clone NAT105, 1:100)
anti–huPD-1-PE-Cyanine7 mAb (BioLegend, Cat. # 621615, Clone A17188B, 1:100)
anti–huHLA-A,B,C (MHC I)-APC mAb (BioLegend, Cat. # 311409, Clone W6/32, 1:100)
anti–huHLA-DR, DP, DQ (MHC II)-FITC mAb (BioLegend, Cat. # 361705, Clone Tü39, 1:100)
anti–moEpCAM-PE-Cyanine7 mAb (BioLegend, Cat. # 118216, Clone G8.8, 1:1000)
anti–huEpCAM-PE mAb (Abcam, Cat. # ab237397, Clone EPR20532-225, 1:100)
anti–moTER-119-APC mAb (BioLegend, Cat. # 116211, Clone TER-119, 1:100)
anti–huCD235a-FITC mAb (BioLegend, Cat. # 349103, Clone HI264, 1:100)
anti–moCD41-PE-Cyanine7 mAb (BioLegend, Cat. # 133915, Clone MWReg30, 1:100)
anti–huCD61-PerCp mAb (BioLegend, Cat. # 336409, Clone VI-PL2, 1:100)
anti–huBLIMP1-Alexa Fluor 488 mAb (R&D Systems, Cat. # IC36081G, Clone 646702, 1:100)
anti–huAID-FITC Ab (Bioss, Cat. # bs-7855R-FITC, 1:100)

Validation

All antibodies used are commercially available and were validated by the respective manufacturer. Validation information of all the antibodies used in this study are available on the provider websites.

Eukaryotic cell lines

Policy information about cell lines and Sex and Gender in Research

Cell line source(s)

ExpiCHO cells (catalog No. A29133, Thermo Fisher Scientific) were utilized by The University of Texas MD Anderson Cancer Center Recombinant Antibody Production Core for construction of RBD-specific human monoclonal antibody-producing cell microcultures.

Authentication

Cell line was authenticated by The University of Texas MD Anderson Cancer Center Recombinant Antibody Production Core.

Mycoplasma contamination

ExpiCHO cells tested negative for mycoplasma contamination at The University of Texas MD Anderson Cancer Center Recombinant Antibody Production Core.

Commonly misidentified lines
(See ICLAC register)

No commonly misidentified cell lines were used.

Animals and other research organisms

Policy information about studies involving animals; ARRIVE guidelines recommended for reporting animal research, and Sex and Gender in Research

Laboratory animals

C57BL/6J (RRID: IMSR_JAX: 000664), NSG (NOD.Cg PrkdcscidIl2rgtm1Wjl/SzJ, RRID: IMSR_JAX:005557)9, NBSGW (NOD.Cg-KitW-41J Tyr + Prkdcscid Il2rgtm1Wjl/ThomJ, RRID: IMSR_JAX:026622)14, NSGW41 (NOD.Cg-KitW-41J Prkdcscid Il2rgtm1Wjl/WaskJ, RRID: IMSR_JAX:026497)13 and JAX NSG huCD34™ (RRID: IMSR_JAX:005557) mice were purchased from The Jackson Laboratory – JAX NSG huCD34™ mice were constructed by grafting γ-irradiated female NSG mice at 3 weeks of age with human cord blood CD34+ cells. We constructed huNSG mice by preconditioning myeloablation of newborn NSG mice (within 48 h of birth) using (1 Gy) γ-radiation, followed by intracardiac injection of purified human cord blood CD34+ cells using a 27-gauge needle. We constructed huNBSGW and huNSGW41 mice by grafting non-γ-irradiated, genetically myeloablated newborn NBSGW and NSGW41 mice (within 48 h of birth) intracardially with human cord blood CD34+ cells. We constructed THX mice by feeding huNBSGW or huNSGW41 mice 17β-estradiol (E2, 3301, Sigma-Aldrich; 1.5 µM in drinking water) ad libitum starting at 14-18 weeks of age (18 weeks in most cases) and continuing thereafter. After 4 weeks of E2-conditioning, huNBSGW or huNSGW41 mice (referred to as THX mice) were ready for experiments or continued on E2 in view of being used at a later time. Most THX mice were constructed using NBSGW mice as only a dozen NSGW41 mice were acquired in 2019 from The Jackson Laboratory before the sale of such mice was discontinued. Such NSGW41 mice were used to construct some Lupus THX mice. Mice used in all experiments were 20 to 24 weeks of age, unless indicated otherwise. Mice used in all experiments were housed in ventilated cage racks with ad libitum access to food and water in a pathogen-free barrier animal vivarium facility at The University of Texas Health Science Center at San Antonio and were free of infection or disease. Housing rooms were maintained at a 14-hour light / 10-hour dark cycle and controlled temperatures of ~22-23 degrees Celsius with 40-60% humidity. Food and water were sterilized.

Wild animals	No wild animals were used in this study.
Reporting on sex	Both male and female mice were used in all experiments and in virtually equal proportions.
Field-collected samples	No field-collected samples were used in this study.
Ethics oversight	All experiments involving mice were performed in compliance with the animal protocol approved by the University of Texas Health Science Center at San Antonio Institutional Animal Care and Use Committee (IACUC Protocol 20200019AR).

Note that full information on the approval of the study protocol must also be provided in the manuscript.

Plants

Seed stocks	No seed stocks were used in this study.
Novel plant genotypes	No plants or plant materials were used in this study.
Authentication	No plants or plant materials were used in this study.

Flow Cytometry

Plots

Confirm that:

☒ The axis labels state the marker and fluorochrome used (e.g. CD4-FITC).

☒ The axis scales are clearly visible. Include numbers along axes only for bottom left plot of group (a 'group' is an analysis of identical markers).

☒ All plots are contour plots with outliers or pseudocolor plots.

☒ A numerical value for number of cells or percentage (with statistics) is provided.

Methodology

Sample preparation	For surface staining, cells from peripheral blood of healthy humans or from peripheral blood, bone marrow, spleen and lymph nodes of humanized mice (THX, huNBSGW, huNSG or JAX NSG huCD34™ mice), were stained with fluorochrome-conjugated mAbs in Hank's Buffered Salt Solution (HBSS, MT21022CM, Fisher Scientific) plus 0.1% bovine serum albumin (BSA, BP1600-100, Fisher Scientific) (BSA-HBSS) for 20 min. After washing, cells were resuspended in BSA-HBSS for flow cytometry. For intracellular staining, B cells and plasmablasts were first surface stained with anti-huCD45 mAb, anti-huCD19 mAb, anti-huCD27mAb, anti-huCD38 and anti-huCD138 mAb, as well as Fixable Viability Dye eFluor™ 780 (65-0865-14, Fisher Scientific). After washing, cells were fixed by resuspension in Cytofix/Cytoperm™ buffer (554655, BD Biosciences, 250 µl) and incubated at 4°C for 45 min. After washing twice in BD Perm/Wash buffer (554723, BD Biosciences) for permeabilization, cells were stained with FITC–anti-huAID pAb (bs-7855R-FITC, Bioss), or Alexa Fluor® 488-anti-huBLIMP1 mAb (clone 646702, IC36081G, R&D Systems) in BD Perm/Wash buffer for 30 min at 4°C. After washing again twice in BD Perm/Wash buffer, cells were resuspended in BSA-HBSS for flow cytometry. All flow cytometry analyses were performed using single cell suspensions.
Instrument	BD FACSCelesta and BD LSRII (BD Biosciences)
Software	FlowJo v10.9
Cell population abundance	The purity of naive huCD19+IgM+IgD+ B cells isolated from healthy human donors and THX mice was at least 98% as verified by flow cytometry.
Gating strategy	FSC-A/SSC-A was used to identify cells of interest based on size and cytoplasmic complexity. FSC-H/FSC-A and SSC-H/SSC-A were used to remove doublets. Cell viability was determined using live/dead stain (e.g., 7-AAD) gating on negative (i.e., live) cells. Human immune cells within the live gate were selected by excluding mouse CD45+ and including human CD45+ cells. B cells were gated based on CD19+ expression within human CD45+ cells. Naive and class-switched B cells were identified based on expression of IgM, IgD, IgG, IgA or IgE within B cells. Class-switched memory B cells were identified based on expression CD27+ and IgD- expression within B cells. Plasmablasts/plasma cells were gated based on CD27+ and CD38+ expression within human CD45+ cells. T cells were gated based on CD3+ expression within human CD45+ cells. Single and double positive CD4 and/or CD8 T cells were identified within CD3+ T cells. T follicular helper cells were identified based on expression of CXCR5+ and PD-1+ within CD4+ T cells. Dendritic cells were gated based on CD11c+ expression and exclusion of CD14 expression within human CD45+ cells. Monocytes/macrophages were gated based on CD14+ expression within

human CD45+ cells. NK cells were gated based on CD56+ expression within human CD45+ cells. Gating strategies for identification of human and mouse CD45+ cells, red blood cells and platelets are provided in Supplementary Figure 1.

☒ Tick this box to confirm that a figure exemplifying the gating strategy is provided in the Supplementary Information.
